# Contrasting the genetic architecture of cardiac glutathione against other organs: unveiling a unique tissue-specific locus

**DOI:** 10.1007/s00335-026-10222-7

**Published:** 2026-05-11

**Authors:** Jessica Strosahl, Susan McClatchy, Gary A. Churchill, Rebecca L. Koch, Steven W. Craig, Robert Pazdro

**Affiliations:** 1 Department of Nutritional Sciences, University of Georgia, 305 Sanford Drive, Athens, GA 30602 USA; 2 The Jackson Laboratory, Bar Harbor, ME 04609 USA

## Abstract

Glutathione is a ubiquitous antioxidant with critical roles in xenobiotic clearance and aerobic metabolism. Reduced glutathione (GSH) is converted to its oxidized counterpart, GSSG, in the process of neutralizing free radicals and maintaining biochemical homeostasis. An imbalance between free radical generation and antioxidant protection leads to oxidative stress and diminished GSH concentrations. Polymorphisms in GSH synthesis and metabolism genes have been associated with cardiovascular disease (CVD) development and progression, highlighting the critical need to explore the link between GSH system genetics and CVD. While those prior studies have focused on canonical GSH genes such as glutathione peroxidases (*GPx*), few studies to this date have investigated whether genes outside of traditional GSH pathways may be impacting the cardiac GSH redox system. Here, we performed high-precision genetic mapping using the Diversity Outbred (DO) mouse stock which revealed novel loci and genes underlying the cardiac GSH system. Mapping results indicated a locus on murine chromosome 14 at 54.240 Mbp associated with cardiac GSH concentrations, and bioinformatics analyses delineated five potential candidate genes: *Slc7a7*, *Myh6*, *Myh7*, *MiR-208a*, and *Nfatc4*. In addition, statistical analyses revealed notable variation in the cardiac GSH redox system under normal physiological conditions and correlations between cardiac and renal GSH phenotypes in the same cohort of mice. Overall, these findings expand our current knowledge of the vital GSH system, reveal novel genetic regulation of cardiac GSH status, and present candidate genes to be tested in future mechanistic studies.

**Supplementary Information:**

The online version contains supplementary material available at 10.1007/s00335-026-10222-7.

## Introduction

Glutathione (GSH) is a tripeptide antioxidant comprised of glutamate, cysteine, and glycine (Lu 2009). Its characteristic antioxidant function stems from the cysteine residue, which participates in oxidation-reduction (redox) reactions through its sulfhydryl group (Forman et al. 2009). Glutathione biosynthesis requires two enzymes – glutamate cysteine ligase (GCL) and glutathione synthetase (GS) – and its intracellular levels are dependent on the availability of its constituent amino acids, the rate of its production and degradation, diet and physical activity, diurnal variations, and other physiological conditions (Espinosa-Díez et al. 2018, Ballatori et al. 2009, Wu et al. 2004). Within the cell, glutathione primarily exists in its thiol-reduced form (GSH) and serves as a cofactor for enzymes such as those in the family of glutathione peroxidases (GPx), which convert GSH to its disulfide-oxidized form (GSSG) while simultaneously neutralizing hydrogen peroxide and other organic hydroperoxides (Kaplowitz and Ookhtens 1985). GSH can also directly scavenge free radicals or form conjugates with xenobiotic compounds to facilitate their excretion (Forman et al. 2009, Lushchak 2012, Boyland and Chasseaud 1969). If the production of reactive oxygen species (ROS) exceeds the ability of GSH and other antioxidant defense systems to counteract them, the cell enters a state of oxidative stress (Pisoschi and Pop 2015). Oxidative stress has been implicated in the development and progression of many chronic diseases, including cardiovascular disease (CVD); in turn, GSH appears to have a protective effect against the same conditions (Andreadou et al. 2020, Griendling et al. 2016, Matuz-Mares et al. 2021, Moris et al. 2017, Shimizu et al. 2004, Damy et al. 2009).

To this point, the majority of research on GSH has focused on tissues with the highest concentrations – notably, the liver, where its role in xenobiotic processing is indispensable. In many other tissues, GSH levels are much lower; for instance, GSH concentrations in the heart are only approximately 12–30% of those in the liver (Zhou et al. 2014, Gould et al. 2018), yet this molecule is nonetheless essential to cardiovascular function, and its redox status has been recognized as a disease biomarker (Matuz-Mares et al. 2021, Angelovski et al. 2023, Masella et al. 2005). For example, lower plasma GSH levels have been identified in CVD patients (Shimizu et al. 2004, Julius et al. 1994), and depleted atrial and blood GSH levels were correlated with New York Heart Association functional class in a cohort of patients undergoing cardiac surgeries (Damy et al. 2009). Furthermore, alterations in GSH enzyme levels and activity have been identified in CVD. Diminished activities of GPx (Espinola-Klein et al. 2007, Schnabel et al. 2005, Cheng et al. 2009, Lapenna et al. 1998, Blankenberg et al. 2003) and glutathione reductase (GR) (Zuzak et al. 2017) have been associated with adverse CVD outcomes such as cardiovascular events in humans, suggesting their potential role in CVD pathogenesis. In addition, elevated levels of serum gamma-glutamyltransferase (GGT), the enzyme responsible for extracellular GSH degradation into its constituent amino acids (Jiang et al. 2013), have been observed in patients with early-stage (Makarewicz-Wujec and Kozlowska-Wojciechowska 2011) and chronic (Ess et al. 2011) heart failure. Even more, longitudinal studies have frequently demonstrated the predictive value of GGT levels for CVD morbidity and mortality (Ruttmann et al. 2005, Pompella et al. 2004). This relationship has been attributed, in part, to the potential pro-oxidant effects of releasing more reactive cysteinyl-glycine residues from extracellular GSH molecules (Ruttmann et al. 2005, Paolicchi et al. 1999). Given the accumulating support for GSH and its core enzymes as biomarkers for heart disease, more studies are needed to better define how cardiac GSH status is regulated.

Altering the expression of core GSH enzymes causes direct effects on cardiovascular health. For instance, deletion of *GCL catalytic subunit (Gclc)* results in embryonic lethality in mice (Shi et al. 2000), yet *Gclc* haploinsufficiency produces viable offspring with impaired endothelium-dependent vasodilation (Espinosa-Díez et al. 2018). This phenotype is a consequence of depleted endothelial GSH, which stimulates the S-glutathionylation of endothelial nitric oxide synthase (eNOS) (Giustarini et al. 2004). Disrupted endothelium-dependent vasodilation contributes to the development of CVD, primarily due to alterations in nitric oxide and superoxide synthesis, both of which are essential vasoactive molecules (Weil et al. 2011, Wang et al. 2022, Chen et al. 2010). In humans, variants in *GCL* have been implicated in CVD risk and severity (Nakamura et al. 2002, Nakamura et al. 2003, Koide et al. 2003), indicating polymorphisms in this gene may serve as genetic risk factors for CVD. Alterations in the *Gpx1* gene also have profound effects on the heart. Following doxorubicin administration, *Gpx1*^−/−^ mice exhibit adverse cardiac effects such as accelerated hypertrophy of the heart (Ardanaz et al. 2010) and impaired contractility and cardiac perfusion (Gao et al. 2008) compared to wild-type controls. In humans, *GPX1* variant alleles have been predictive of cardiovascular disease risk across several studies (Zhang et al. 2014, Hamanishi et al. 2004, Souiden et al. 2016, Tang et al. 2008). Despite these findings, researchers have yet to define the full set of genes and variants that directly affect cardiac GSH and its redox balance. If novel genes were to be associated with cardiac GSH, they would provide key insight into the pathogeneses of heart diseases.

In the present study, we sought to define the genetic regulation of heart GSH and how it relates to that of other organs. Previously, our laboratory performed quantitative trait locus (QTL) mapping of GSH in its most commonly studied organs, the liver and kidney. We discovered novel loci underlying hepatic and renal GSH phenotypes, such as on murine chromosome 16 at 8.998 Mbp for hepatic GSH/GSSG balance (Gould et al. 2021) and on murine chromosome X at 51.602 Mbp for renal GSH concentrations (Gould et al. 2021). Those studies were conducted in the Diversity Outbred (DO) mouse stock, a heterogeneous stock containing over 37.8 million single nucleotide polymorphisms (SNPs), which allows for high-resolution mapping and identification of candidate genes (Keane et al. 2011, Churchill et al. 2012, Schmidt 2015). Here, we outline the genetic architecture of cardiac GSH using the DO stock, and we contrast it against the genetics of GSH homeostasis in the liver and kidney. We explored statistical relationships between cardiac, hepatic, and renal GSH metabolism (Gould et al. 2021, Gould et al. 2021) and identified significant correlations between the cardiac and renal systems. To our knowledge, this is the first study to identify novel QTL regulating cardiac GSH levels – distinct from the loci that underlie GSH status in the liver and kidney – and provides candidate genes to guide future critical studies on the GSH antioxidant system.

## Methods

### Mice

Male and female Diversity Outbred (DO) mice (J: DO; JAX^®^ #009376) from generations 30, 32, and 35 were purchased from The Jackson Laboratory (Bar Harbor, ME USA). The DO population was derived from a genetic cross of eight inbred strains: A/J (AJ), C57BL/6J (B6), 129S1/SvImJ (129), NOD/ShiLtJ (NOD), NZO/HlLtJ (NZO), CAST/EiJ (CAST), PWK/PhJ (PWK), and WSB/EiJ (WSB). Mice were received at 4 weeks of age and provided *ad libitum* access to water and standard chow (LabDiet^®^, St. Louis, MO USA, product 5053) under a 12 h light-dark cycle. These conditions were maintained until the mice were sacrificed at 5–6 months of age. All mice were fasted for 3–4 h during the light cycle and then were humanely euthanized by cervical dislocation. Heart tissue was collected for subsequent analysis (100 males, 108 females). The University of Georgia Institutional Animal Care and Use Committee (IACUC) approved all methods and procedures involving animals in accordance with the ethical standards of the institution (AUP #A2016-07-016), and all methods and procedures were carried out in accordance with the National Institutes of Health guide for the care and use of Laboratory animals (NIH Publications No. 8023, revised 1978).

### Assessment of cardiac GSH, GSSG, total glutathione and redox ratios

Cardiac tissue samples were immediately collected from each mouse following euthanasia. The tissues were rinsed with ice-cold PBS, briefly blotted dry on a paper towel, and rapidly flash-frozen in liquid nitrogen. Within 12 h of collection, samples were homogenized in PBS containing 10 mM diethylenetriaminepentaacetic acid (DTPA) and acidified with an equal volume of ice-cold 10% perchloric acid containing 1 mM DTPA (Gould et al. 2018, Park et al. 2010). The acidified homogenates were centrifuged at 15,000 RPM for 15 min at 4 °C, and the resulting supernatant was filtered. Filtered supernatants were stored at − 80 °C until further analysis. HPLC with electrochemical detection was used to measure GSH and GSSG concentrations in each sample (Dionex Ultimate 3000, Thermo Fisher Scientific, Waltham, MA USA) based on previously published methods (Park et al. 2010). A conditioning cell was set to + 500 mV and placed immediately before the boron-doped diamond cell set at + 1475 mV with a cleaning potential at + 1900 mV between samples. The mobile phase was comprised of 4.0% acetonitrile, 0.1% pentafluoropropionic acid, and 0.02% ammonium hydroxide. The flow rate was set at 0.22 mL/min with an injection volume of 5.0 μL. Peak quantification was performed using external GSH and GSSG standards, external calibration, and the Chromeleon Chromatography Data System Software (Dionex Version 7.2, Thermo Fisher Scientific, Waltham, MA USA). All glutathione concentrations (i.e., GSH and GSSG) were standardized to total protein (Pierce BCA Protein Assay, Thermo Fisher Scientific, Waltham, MA USA) and expressed in nmol/mg protein. Total glutathione concentrations were calculated as [GSH]+[2GSSG]. Next, the ratio of GSH/GSSG was calculated. The redox potential *E*_h_ of the redox couple is frequently used to better understand the redox status of the cell (García-de-la-Asunción et al. 2016, Rebrin et al. 2003, Ziegler et al. 2001, Rost and Rapoport 1964, Wang et al. 1993, Khazim et al. 2013). E_h_ of the GSSG-GSH pair (2GSH→GSSG +2e^−^ + 2H^+^) in each heart sample was calculated using the Nernst equation at 40°C:

$$Eh = E0 + RTnF\ln \left[ {\left( {ox} \right)\left( {red} \right)} \right]$$

*E*ℎ = measured cell potential, *E*0 = standard electrode potential for GSSG/2GSH (− 264 mV at pH 7.4 (Ziegler et al. 2001, Rost and Rapoport 1964, Wang et al. 1993)), *R* = gas constant (8.3145 J x mol^−1^ x K^− 1^), *T* = temperature in Kelvin (313.15 K), *n* = number of electrons transferred (2), *F* = Faraday’s constant (96485 C x mol^− 1^), *ox* = molar concentration of oxidant (GSSG), and *red* = molar concentration of reductant (GSH). Tissue concentrations of GSH and GSSG (nmol/mg protein) were expressed in molar concentrations (García-de-la-Asunción et al. 2016, Rebrin et al. 2003) using a conversion factor of 500 µL/mg of protein. The final equation used to calculate E_h_ (mV) for the GSSG-GSH couple was:

$$Eh\left( {mV} \right) = - 264 + 31\log \left[ {\left( {GSSG} \right)\left( {GSH} \right)2} \right]$$

### Genotyping

DNA was isolated from tail tips collected at sacrifice and genotyped using the third-generation Mouse Universal Genotyping Array (GigaMUGA) (Morgan et al. 2016) performed by GeneSeek (Neogen Genomics, Lincoln, NE USA, 68504). GigaMUGA is a 143 K-probe array built on the Illumina Infinium II platform and has been specifically adapted for use in the DO stock.

### Quantitative trait loci (QTL) mapping

Genome scans were performed using 208 DO mice. All phenotypic data were *z*-score transformed to ensure normality (Svenson et al. 2012). Founder haplotypes were reconstructed using a Hidden Markov Model as implemented in the R/qtl2 package (Broman et al. 2019). Following the default parameters developed for R/qtl2, diplotype probabilities were calculated on a fixed grid of pseudomarkers at 0.2 cM intervals, assuming a genotyping error probability of 0.002 and utilizing the Carter-Falconer map function (Broman et al. 2019).

Next, QTL mapping was conducted in R/qtl2 using a linear mixed model with the *scan1* function. Sex was included as an additive covariate for each phenotype scan. Each scan accounted for kinship among the DO mice using the “leave one chromosome out” (LOCO) method (Broman et al. 2019, Yang et al. 2014). Phenotype-specific significance thresholds were determined by performing 1000 permutations using the *scan1perm* function (*p* ≤ 0.05 and *p* ≤ 0.20) (Broman et al. 2019, Sen and Churchill 2001, Churchill and Doerge 1994). Genome-wide *p*-values for each peak were calculated using the *pull_fwer_pval* function from the musppr package (available at https://github.com/gkeele/musppr). The *find_peaks* and *bayes_int* functions in R/qtl2 were employed to identify 95% Bayesian credible intervals for each QTL peak (Broman et al. 2019, Sen and Churchill 2001). We used Best Linear Unbiased Predictors (BLUPs), representing inferred founder allele effects from the QTL model, to visualize allele effects at each peak using the *scan1blup* function (Robinson 1991).

### Candidate gene analysis

To narrow down potential candidate genes within suggestive loci, we utilized bioinformatics databases containing public expression, phenotypic, and functional annotations based on previous methods (Gould et al. 2021, Recla et al. 2019, Recla et al. 2014) (Supplementary Table S1). All protein-coding genes within suggestive loci ± 1 Mbp were identified through the Mouse Genome Informatics (MGI) database of published mouse gene annotations. First, we filtered out those genes not known to be expressed in adult murine cardiac tissue based on annotations from the Gene eXpression Database (GXD) (Baldarelli et al. 2020) through MGI (Blake et al. 2021) and the European Molecular Biology Laboratory European Bioinformatics Institute (EMBL-EBI) (Thakur et al. 2022). Second, we utilized Gene Ontology (GO) functional annotations (Consortium 2017, Ashburner et al. 2000) through MGI (Blake et al. 2021) to determine which genes were implicated in relevant biological processes. Finally, we compiled phenotypic annotations from Ensembl BioMart (Kinsella et al. 2011) and the MGI database, including mammalian phenotype (MP) (Smith et al. 2004) and disease ontology (DO) (Schriml et al. 2022) terms.

### SNP association

To refine the genomic regions identified in the initial genome scans, we performed SNP association mapping within the 95% Bayesian credible intervals of identified QTL. Using the *scan1snps* function, we performed variant association analysis by projecting the 8-way founder probabilities onto the specific SNPs from the Collaborative Cross variant database (Broman et al. 2019). A query function (*query_variants*) was utilized to retrieve all known variants within ± 1 Mbp of the 95% Bayesian credible intervals for each QTL peak.

To identify potential causal variants, we calculated the LOD scores for all SNPs in these regions and identified the top SNPs using the *top_snps* function. These SNPs were further cross-referenced with Ensembl gene annotations and MGI databases to identify protein-coding genes containing or adjacent to the most significant variants. We visualized the association results alongside MGI gene models using *plot_snpasso* to identify genes within a 1.5-LOD drop of the top-associated SNP.

Finally, we compiled founder allele effects for all variants within the identified candidate genes that contained available expression, functional, and phenotypic annotations using the Founder Variant Portal (https://churchilllab.jax.org/foundersnps/search).

### Statistical analysis

RStudio version 2023.03.1 + 446 (RStudio, PBC., Boston, MA USA) and R version 4.3.0 (R Foundation for Statistical Computing, Vienna, Austria) were used for data normalization and to calculate correlations between variables. Spearman’s rho (ρ) was calculated for each relationship. The relationship between variables was considered statistically significant if the *p*-value was less than 0.05. Next, we performed unsupervised hierarchical clustering on the Spearman’s correlation matrix. Distances were calculated using Spearman’s correlation distance, and clusters were merged using the complete linkage method as implemented in the ComplexHeatmap package. The resulting dendrograms were used to visualize the similarity of redox profiles across phenotypes. All genotype data, genotype probabilities, and marker information are publicly available through figshare (https://figshare.com/collections/Pazdro-DO-Genotypes-R01-Glutathione-Redox-System/5360501/1). All source code, phenotype data, and other files used in QTL analyses are available through a public GitHub repository (https://github.com/smcclatchy/pazdro-glutathione-redox/tree/main).

## Results

### Cardiac GSH concentrations and redox status vary significantly among DO mice

**Fig. 1:**
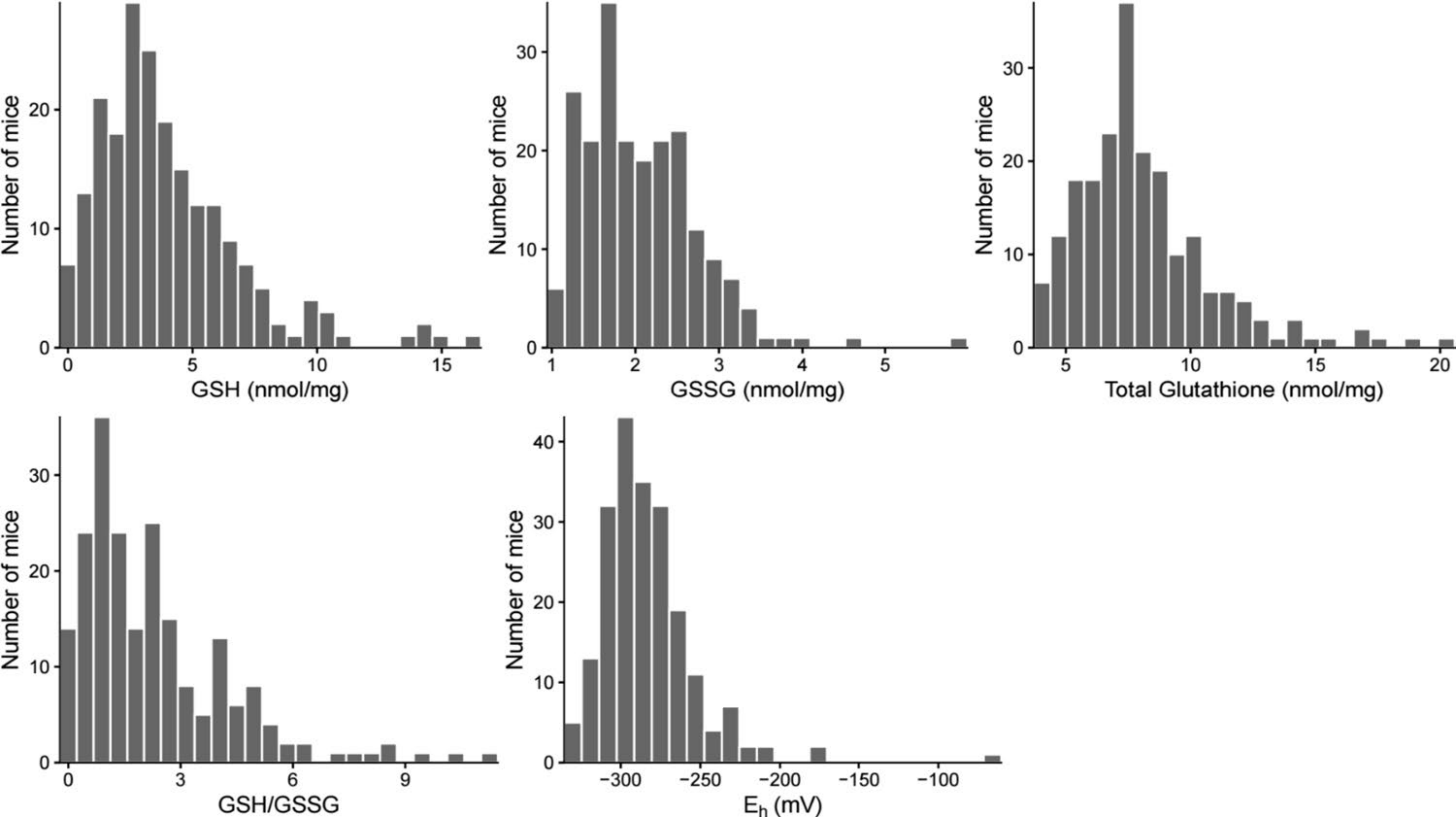
Cardiac GSH concentrations and redox status vary significantly in a genetically diverse mouse population. Cardiac tissue was isolated from Diversity Outbred mice (*N* = 208). Concentrations of GSH and GSSG were quantified, and total glutathione, GSH/GSSG, and the redox potential (E_h_) were calculated. GSH, GSSG, and total glutathione are expressed as nmol/mg protein, and E_h_ is expressed in millivolts (mV). All values are plotted against the number of mice (*N* = 208)

We measured cardiac GSH, GSSG, and derivative redox phenotypes in a cohort of DO mice (*N* = 208; Table 1). Cardiac GSH varied widely (0.000720 to 15.991 nmol/mg), as did the GSH/GSSG ratio (0.000277 to 11.252) (Fig. 1). Cardiac GSSG and total glutathione varied 5.7-fold (1.018 to 5.802 nmol/mg) and 5.5-fold (3.723 to 20.414), respectively. Likewise, the redox potential varied from -333.908 to -65.146 mV. We did not observe sex specific effects for any trait (Supplementary Table S2 and S3).

**Table 1:** Descriptive statistics for cardiac GSH concentrations and redox status in DO mice

Phenotype	*N*	x̄	Median	SD	Min	Max
Total Glutathione (nmol/mg)	208	8.08	7.52	2.80	3.72	20.41
GSH (nmol/mg)	208	3.97	3.25	2.92	0.00072	15.99
GSSG (nmol/mg)	208	2.06	1.93	0.69	1.018	5.80
GSH/GSSG	208	2.29	1.64	2.00	0.00028	11.25
E_h_ (mV)	208	-282.90	-286.56	30.25	-333.91	-65.15

Cardiac concentrations of GSH (nmol/mg protein) and GSSG (nmol/mg protein) were quantified in samples collected from DO mice (100 males; 108 females) by HPLC. Total glutathione concentrations were then calculated (GSH + 2GSSG), as were GSH/GSSG and E_h_ (mV). Basic descriptive statistics were determined in RStudio

**Fig. 2:**
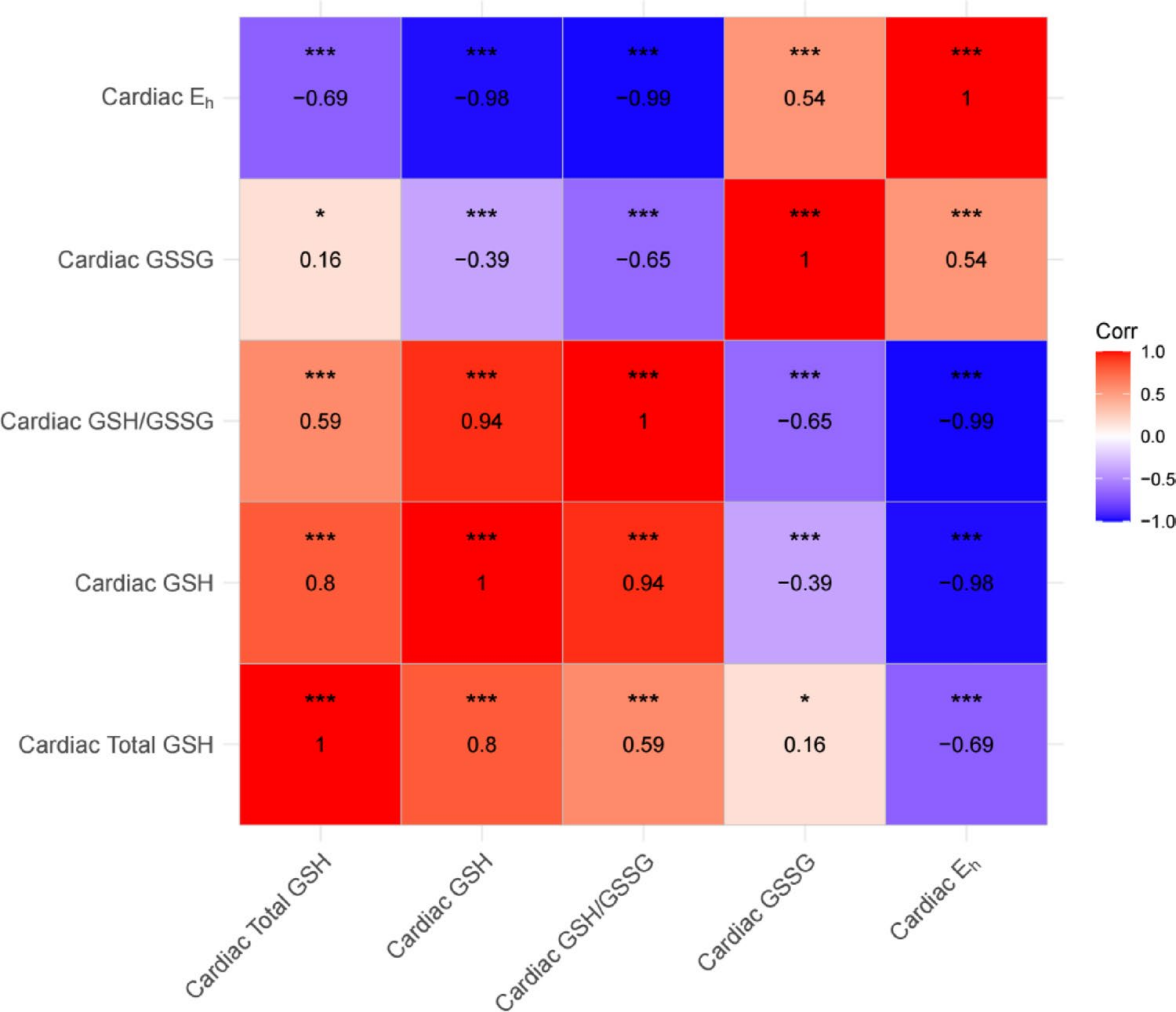
Correlations among cardiac GSH system markers in a large cohort of DO mice. Spearman’s rho (ρ) was calculated for each variable combination and is listed within each corresponding box. GSH, GSSG, and total glutathione concentrations were standardized as nmol/mg protein. E_h_ was expressed as mV. P-values are denoted by the following: *** < 0.001, ** < 0.01, * < 0.05

We explored correlations between cardiac GSH phenotypes and found several significant relationships (Supplementary Table S4; Fig. 2). As expected, cardiac GSH derivatives exhibited positive correlations with cardiac GSH, including GSH/GSSG (ρ = 0.94, *p* < 0.001) and total GSH (ρ = 0.80, *p* < 0.001). Cardiac GSH was negatively correlated with cardiac GSSG (ρ = − 0.39, *p* < 0.001) and redox potential (ρ = − 0.98, *p* < 0.001). Cardiac GSSG was negatively correlated with cardiac GSH/GSSG (ρ = − 0.65, *p* < 0.001), and cardiac GSSG was positively correlated with total GSH (ρ = 0.16, *p* < 0.05) and redox potential (ρ = 0.54, *p* < 0.001). Expectedly, cardiac total GSH was positively correlated with cardiac GSH/GSSG (ρ = 0.59, *p* < 0.001) and negatively correlated with the cardiac redox potential (ρ = − 0.69, *p* < 0.001). Lastly, cardiac redox potential was negatively correlated with cardiac GSH/GSSG (ρ = − 0.99, *p* < 0.001). These data highlight substantial variation in cardiac GSH phenotypes among DO mice and confirm expected correlations between redox markers.

### QTL mapping of cardiac GSH phenotypes

**Fig. 3:**
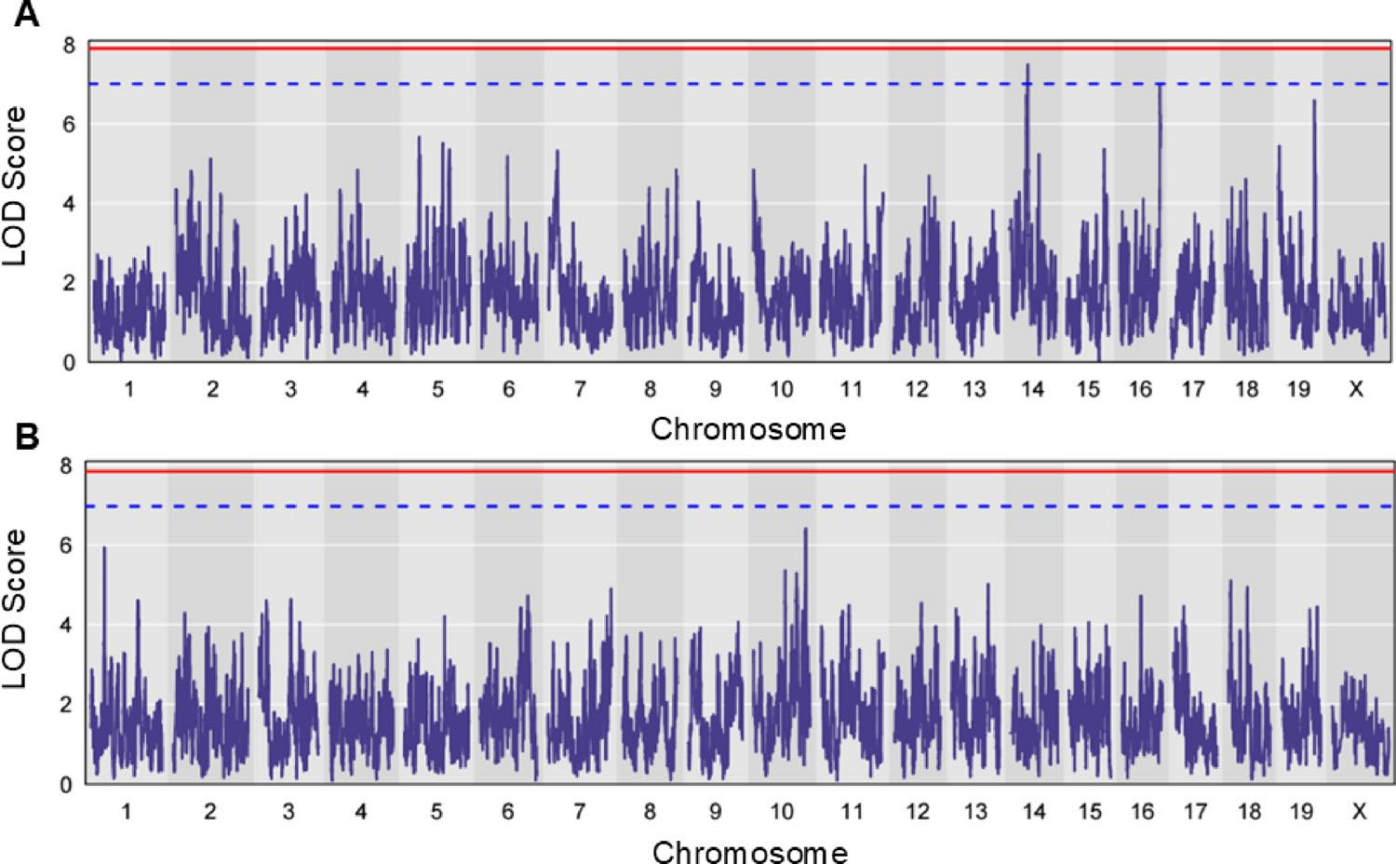
QTL results for reduced and oxidized glutathione. Genome-wide scans of cardiac **A** GSH (nmol/mg) and **B** GSSG (nmol/mg). Chromosomes are plotted on the x-axis. LOD scores are plotted on the y-axis. Red and blue dashed significance lines were calculated by running 1,000 permutations at a significance (α) level of 0.05 and 0.20, respectively

QTL analysis was performed using R/qtl2 on cardiac GSH, GSSG, total GSH, GSH/GSSG ratio, and redox potential (Fig. 3; Supplementary Figures S1–S7). We ran 1000 permutations on all scans to calculate autosomal significance thresholds (*p* ≤ 0.05 and *p* ≤ 0.20). While no peaks surpassed the LOD score thresholds of *p* ≤ 0.05 or *p* ≤ 0.20, several peaks did surpass the LOD score = 6 threshold, a common convention used to approximate suggestive peaks in DO QTL mapping (Supplementary Table S5). The cardiac GSH scan contained three suggestive peaks on mouse chromosome 14 at 54.240 Mbp, chromosome 16 at 96.736 Mbp, and chromosome 19 at 57.209 Mbp (LOD scores of 7.492, 6.969, and 6.596, respectively). The cardiac GSSG scan had one suggestive peak located on mouse chromosome 10 at 124.10385 Mbp (LOD score 6.411). Two suggestive peaks were revealed by the GSH/GSSG ratio scan on mouse chromosome 5 at 38.839 Mbp and chromosome 14 at 54.240 Mbp (LOD scores 6.104 and 6.154, respectively). Three peaks surpassed the LOD score = 6 threshold on the redox potential scan, mirroring the cardiac GSH scan, located on mouse chromosome 14 at 54.239 Mbp, chromosome 16 at 96.736 Mbp, and chromosome 19 at 57.209 Mbp (LOD scores 6.969, 6.574, and 6.147, respectively). Genome-wide *p*-values for each QTL peak were calculated (Supplementary Table S5). Given that total GSH, GSH/GSSG, and redox potential are derivatives of GSH and GSSG, we focused on the genome-wide GSH scan which revealed the QTL with the highest LOD score across all phenotypes. Founder allele effects and bioinformatic analysis of candidate genes from other suggestive peaks can be found in Supplementary Tables S6–S8 and Supplementary Figures S1–S7. We also calculated overall heritability for each phenotype which can be found in Supplementary Table S9 (GSH = 0.29, GSSG = 0.21, total GSH = 0.09, GSH/GSSG = 0.34, redox potential = 0.46).

**Fig. 4:**
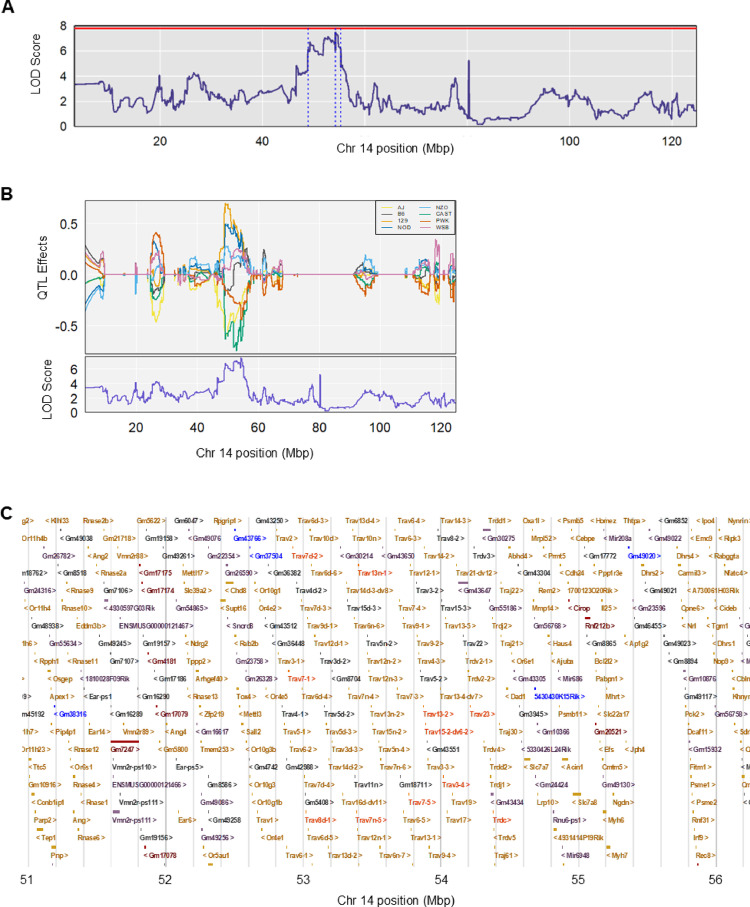
High-resolution QTL mapping of cardiac GSH highlights a suggestive peak on chromosome 14. **A** Chromosome 14 cardiac GSH (nmol/µg) QTL scan. The permutation-derived threshold is indicated by the red colored line at significance (α) level 0.05. The Bayesian credible interval is indicated by the dashed blue lines. Chromosome 14 position (Mbp) is plotted on the x-axis. LOD scores are plotted on the y-axis. **B** Founder allele QTL effects show that the 129 and NOD alleles contribute to a higher cardiac GSH concentration while the PWK, AJ, and CAST alleles contribute to a lower cardiac GSH concentration. Each colored line represents one of eight DO founder alleles as indicated in the legend. The differences between strains are considered significant when the LOD score (bottom plot) crosses the significance thresholds established in panel A. **C** Genes within the chromosome 14 cardiac GSH QTL scan plotted using data from Mouse Genome Informatics. Chromosome 14 position (Mbp) is plotted on the x-axis

The genome-wide cardiac GSH scan contained the QTL with the highest LOD score (7.492) located on mouse chromosome 14 within the QTL interval of 48.909–55.303 Mbp (Fig. 4A). This peak was just short of meeting significance (*p* ≤ 0.05 LOD score threshold = 7.819), and its genome-wide p-value was 0.094. Estimated founder allele effects indicated that the 129 and NOD alleles were associated with higher cardiac GSH concentrations, whereas the AJ, CAST, and PWK alleles were associated with lower cardiac GSH concentrations (Fig. 4B).

Interestingly, using a more sensitive *find_peaks* parameter (peakdrop = 1) revealed that this locus likely represents a complex regulatory region with two distinct signals. In addition to the primary peak, a secondary proximal peak was identified at 49.253 Mbp (LOD score = 6.721; CI: 48.819–51.621 Mbp). Analysis of founder allele effects across the region showed a divergent pattern between these two signals; notably, the PWK-driven low-GSH association observed at the primary peak was absent at the proximal 49.253 Mbp locus, indicating a shift in the allelic series.

To investigate the genes ± 1 Mbp of the primary QTL interval, we utilized data from Mouse Genome Informatics (MGI) and plotted these genes in R/qtl2 (Fig. 4C). Protein-coding genes and functional RNA within the window were screened based on available expression, functional, and phenotypic data (Supplementary Table S10). The QTL interval contained 590 possible candidate genes: 181 protein-coding genes, 105 non-coding RNA genes, 76 pseudogenes, 1 RNase P RNA gene, 16 unclassified genes, and 211 gene segments. 542 of the 590 genes within this interval were excluded due to limited adult cardiac expression annotations from EMBL-EBI and MGI. Of the remaining 48 genes, 38 genes were excluded due to limited functional annotations. Five of the remaining 10 genes contained phenotypic annotations related to cardiac function or redox status: *solute carrier family 7 (cationic amino acid transporter*,* y+ system)*,* member 7 (Slc7a7)*,* myosin heavy polypeptide 6*,* cardiac muscle alpha (Myh6)*,* microRNA 208a (MiR-208a)*,* myosin heavy polypeptide 7*,* cardiac muscle beta (Myh7)*,* and nuclear factor of activated T cells*,* cytoplasmic*,* calcineurin dependent 4 (Nfatc4)*. *Myh6* encodes the alpha heavy chain subunit of cardiac myosin (Granados-Riveron et al. 2010) and is associated with many cardiac-related phenotypes in mice, including abnormal myocardial fiber morphology (MP:0000278), cardiac hypertrophy (MP:0001625), dilated cardiomyopathy (MP:0001625), cardiac fibrosis (MP:0001625), and decreased cardiac stroke volume (MP:0011952). A query for associations with *Myh6* in humans revealed relationships with several cardiac-related phenotypes, such as adult heart development (GO:0007512), cardiac muscle contraction (GO:0060048), cardiac muscle hypertrophy in response to stress (GO:0014898), and regulation of heart contraction (GO:0008016). Variants in this gene have been associated with several cardiovascular diseases, including septal defects (Xia et al. 2019, Huang et al. 2021, Zuo et al. 2022), hypoplastic left heart syndrome (Theis et al. 2015, Tomita-Mitchell et al. 2016), and familial dilated cardiomyopathy (Carniel et al. 2005, Granados-Riveron et al. 2010, Klos et al. 2017). *Myh7* encodes the beta heavy chain subunit of cardiac myosin (Klaassen et al. 2008). A search in Ensembl Biomart revealed associations between this gene and congestive heart failure (MP:0006138) in mice. *Myh7* was associated with adult heart development (GO:0007512), cardiac muscle contraction (GO:0060048), cardiac muscle hypertrophy in response to stress (GO:0014898), regulation of heart rate (GO:0002027), amongst other cardiac-related associations in humans. Variants in *Myh7* have been associated with hypertrophic cardiomyopathy (Velicki et al. 2020, Garfinkel et al. 2018, Kraft et al. 2016) and a form of primary cardiomyopathy termed left ventricular noncompaction (Klaassen et al. 2008). MiR-208a is encoded within an intron of *Myh6* and is a cardiac-specific microRNA (miRNA) that plays a role in cardiac remodeling (Kura et al. 2020). We identified four cardiac-related associations with *MiR-208a* in mice: abnormal cardiovascular system physiology (MP:0001544), decreased cardiac muscle contractility (MP:0005140), abnormal myocardium layer morphology (MP:0005329), and abnormal heart left ventricle morphology (MP:0003921). *MiR-208a* is associated with cardiac conduction *(*GO:0061337), cardiac muscle cell development (GO:0055013), heart growth (GO:0060419), and regulation of heart growth (GO:0060420) in humans. *Slc7a7* encodes a protein that functions as the light subunit of an amino acid transporter that moves cationic and large neutral amino acids from the cell to the extracellular space (Rotoli et al. 2018). A search in Ensembl Biomart revealed that *Slc7a7* is associated with cardiac hypertrophy (MP:0001625) in mice. In humans, *Slc7a7* is associated with several GO terms related to amino acid transport, such as amino acid transmembrane transport (GO:0003333) and glycine transmembrane transport activity (GO:0015187), potentially influencing glutathione metabolism indirectly (Kinsella et al. 2011). Variants in this gene result in lysinuric protein intolerance, a recessively inherited aminoaciduria (Rotoli et al. 2018). We were unable to identify existing literature supporting the relationship between *Slc7a7*, oxidative stress, and the cardiovascular system. Lastly, *Nfatc4*, a member of the nuclear factor of activated T-cell (NFAT) family of transcription factors, encodes a transcription factor with roles in embryonic heart development and cardiac hypertrophy (Molkentin 2000, Graef et al. 2001). A query for associations with mouse phenotypes revealed associations with increased heart weight (MP:0002833) and cardiac hypertrophy (MP:0001625). *Nfatc4* was associated with heart development (GO:0007507), vascular-associated smooth muscle cell development (GO:0097084), and vascular-associated smooth muscle cell differentiation (GO:0035886) in humans. Taken together, these results identify a suggestive QTL on chromosome 14 and define a 6.4 Mbp candidate interval containing five genes with public expression, functional, and phenotypic annotations that warrant further investigation as potential drivers of cardiac glutathione variation.

Of note, no variants within any of the five candidate genes mirrored the founder allele effects pattern observed at the QTL (Supplementary Material 2). In addition, we queried all known variants within ± 1 Mbp of the 95% Bayesian credible interval (Supplementary Figure S8), but the *top_snps* function did not identify any variants within the top five candidate genes (Supplementary Table S11).

### Markers of the cardiac and renal GSH systems are significantly correlated, yet the heart shares no overlapping QTL with other tissues

**Fig. 5:**
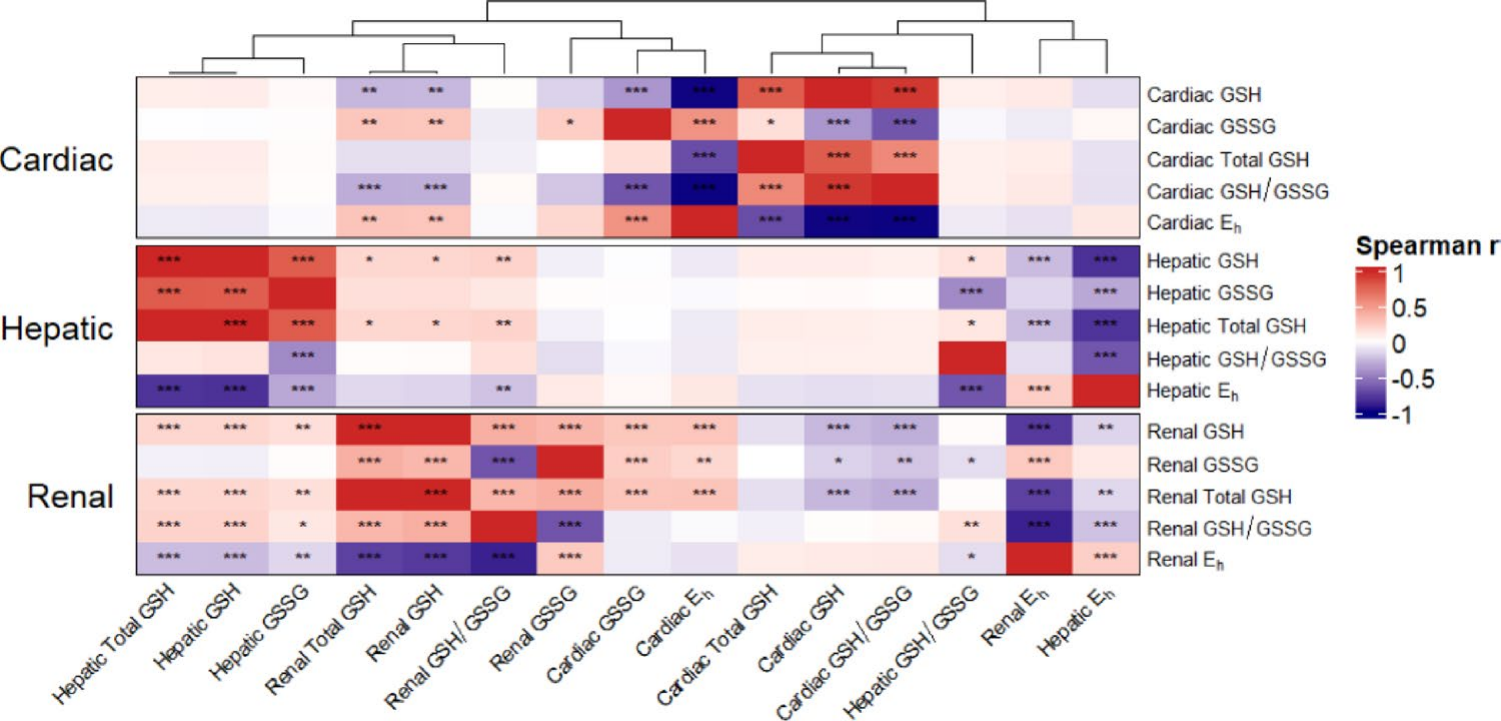
Cardiac GSH markers are correlated with renal GSH markers but not with hepatic GSH markers in the same cohort of DO mice. Spearman’s rho (ρ) was calculated for each variable combination. The heatmap is organized by tissue blocks on the y-axis (cardiac, hepatic, and renal), with the top dendrogram representing unsupervised hierarchical clustering of phenotypes based on their correlation profiles across all tissues. Asterisks indicate statistical significance: *P < 0.05, **P < 0.01, and ***P < 0.001. The color scale represents the strength and direction of the relationship, ranging from navy blue (strong negative correlation, ρ = − 1) to red (strong positive correlation, ρ = 1). GSH, GSSG, and total glutathione concentrations were standardized as nmol/mg protein. E_h_ was expressed as mV

To understand tissue-specific differences in glutathione status, we performed Spearman’s correlation analysis and unsupervised hierarchical clustering of cardiac GSH measurements with those measured in hepatic and renal tissues in the same cohort of mice (Gould et al. 2021, Gould et al. 2021). The resulting dendrogram (Fig. 5; top x-axis) demonstrated a distinct hepato-renal regulatory axis, where hepatic and renal phenotypes clustered together as previously reported by our group (Gould et al. 2021). In contrast, markers of the cardiac GSH system exhibited relative independence from hepatic markers, while showing selective, significant correlations with renal markers. We observed several correlations between cardiac and renal GSH markers, but no significant correlations were found between cardiac and hepatic GSH systems. Specifically, cardiac GSH was negatively correlated with renal GSH (ρ = − 0.26, *p* < 0.001), renal GSSG (ρ = − 0.17, *p* < 0.05) and renal total GSH (ρ = − 0.27, *p* < 0.001). Cardiac GSSG was positively correlated with renal GSSG (ρ = 0.25, *p* < 0.001), renal GSH (ρ = 0.28, *p* < 0.001), and renal total GSH (ρ = 0.28, *p* < 0.001). Cardiac GSH/GSSG was negatively correlated with renal GSH (ρ = − 0.30, *p* < 0.001), renal GSSG (ρ = − 0.21, *p* < 0.01), and renal total GSH (ρ = − 0.30, *p* < 0.001). Finally, cardiac redox potential was positively correlated with renal GSH (ρ = 0.28, *p* < 0.001), renal GSSG (ρ = 0.25, *p* < 0.001), and renal total GSH (ρ = 0.28, *p* < 0.001). We also compiled all significant and suggestive QTL intervals from hepatic and renal GSH systems to screen for overlap with cardiac QTL intervals (Supplementary Table S12). Despite the correlations identified between cardiac and renal GSH phenotypes, we did not identify any overlapping QTL intervals between the cardiac system and either the renal or hepatic systems. Ultimately, the presence of correlations between cardiac and renal GSH markers in the absence of shared QTL suggests that while these systems may be physiologically linked, the genetic architecture regulating cardiac glutathione status is distinct from that of other major metabolic organs.

## Discussion

In the present study, we sought to define the genetic architecture of the cardiac GSH system and contrast it against previously discovered loci in the liver and kidney using QTL mapping. We documented a novel locus underlying cardiac GSH and utilized public expression, functional, and phenotypic annotations to identify candidate genes within this interval. Furthermore, statistical analysis revealed correlations between cardiac GSH measurements and the previously defined renal GSH system. Through our comprehensive QTL mapping approach, we expand the current genetic model of the GSH system and provide evidence for novel regulation of cardiac GSH.

We identified a novel locus on chromosome 14 at 54.240 Mbp underlying cardiac GSH and delineated five potential candidate genes within this locus: *Slc7a7*, *Myh6*, *Myh7*, *MiR-208a*, and *Nfatc4*. All five candidate genes had cardiac-specific expression, functional, and phenotypic annotations. However, a literature search did not reveal a direct relationship between *Slc7a7*, oxidative stress, and the cardiovascular system, while it did support relationships for the remaining four candidate genes. *Myh6* and *Myh7* encode the alpha and beta subunits of myosin heavy chain (α-MHC and β-MHC, respectively) and are critical to cardiac contractility (Warkman et al. 2012). In mammals, these genes are arranged in tandem and are reciprocally regulated throughout development and adulthood in the ventricles (Warkman et al. 2012, Pandya et al. 2010). β-MHC is predominantly expressed in the fetal ventricle, whereas α-MHC is the primary isoform in the adult ventricle (Pandya et al. 2010). However, a change in the expression pattern of these isoforms – termed the “α-MHC to β-MHC switch” – has been associated with cardiac hypertrophy in both rodents (Nadal-Ginard and Mahdavi 1989, Chien et al. 1991) and humans (Nadal-Ginard and Mahdavi 1989, Rawal et al. 2019, Gupta 2007). Furthermore, variants in these genes have been associated with cardiac hypertrophy and other myopathies in humans (Liu and Wang 2014, Ahmad et al. 2005), and both genes are commonly used as markers of cardiac hypertrophy (Morkin 2000, Magrì et al. 2020). Despite the well-documented switch in the expression of these isoforms during cardiac hypertrophy, few studies have reported a direct link to GSH metabolism. Shanmugam et al. observed the α-MHC to β-MHC switch and an increase in the GSH redox potential in a mouse model of pathological cardiac hypertrophy caused by chronic reductive stress (Shanmugam et al. 2020). In addition, GSH treatment was protective against doxorubicin (DOX)-induced cardiotoxicity in mice and decreased cardiac *Myh7* transcript levels compared to non-GSH treated controls (Lee et al. 2023). These data suggest an interplay between *Myh6*,* Myh7*, and cardiac GSH during physiological stress, although the mechanism is not fully understood.

We identified another candidate gene within this locus – *MiR-208a –* located within an intron of *Myh6.* MiRNAs are naturally occurring, noncoding small RNAs ranging from 21 to 25 nucleotides in length that play a role in post-transcription gene expression (O’Brien et al. 2018). Several redox-sensitive miRNAs have been shown to exhibit altered expression signatures in animal models of hypertrophy, including miR-208a (Kura et al. 2020, Liu and Wang 2014, Van Rooij et al. 2006, Ikeda et al. 2009, Zhao et al. 2007). In metabolically challenged cardiomyocytes, *MiR-208a* suppression was protective against cardiac mitochondrial dysfunction (Mekala et al. 2021). Deletion of the gene in mice subjected to hypertrophic stress resulted in resistance to cardiac remodeling and suppression of cardiac *Myh7* (Rooij et al. 2007), while transgenic overexpression of *MiR-208a* was sufficient to induce cardiac hypertrophy (Callis et al. 2009). Furthermore, miR-208a levels were elevated in patients with diabetic cardiomyopathy, and interestingly, the same study revealed that miR-208a upregulation preceded the α-MHC to β-MHC switch in diabetic mouse hearts (Rawal et al. 2019). Although these data clearly indicate the protective effect of miR-208a against hypertrophy, it is not clear how oxidative stress is involved. To better characterize how miR-208a is related to oxidative stress in the cardiovascular system, we searched for miRNA-disease associations in the Human MicroRNA Disease Database (Cui et al. 2024). Several entries revealed associations between miR-208a, CVDs, and oxidative stress. miR-208a down-regulation was found to increase SOD and GPx activity, increase GSH levels, and decrease ROS content using an in vivo model of ketamine-induced cardiotoxicity (Yuan et al. 2019). Additional analyses revealed that miR-208a mediated oxidative stress through Notch/NF-κB signaling pathways by *CDH9* inhibition (Yuan et al. 2019). Moreover, in a study of cardiovascular patients, *MiR-208a* transcript levels and the activity of CAT, SOD, and GPx were significantly increased compared to controls (Mohammadi et al. 2021). Identification of *MiR-208a*, *Myh6*, and *Myh7* as candidate genes within the cardiac GSH locus informs future investigations into the shared mechanisms through which these genes influence redox biology and CVD development.

The last candidate gene identified in this locus, *Nfatc4*, encodes a transcription factor that upon dephosphorylation translocates to the nucleus to regulate the expression of hypertrophic genes (Molkentin et al. 1998, Li et al. 2018, Liu et al. 2015, Li et al. 2016). Overexpression of *Nfatc4* in mice results in severe cardiac hypertrophy (Molkentin 2000, Graef et al. 2001, Bushdid et al. 2003), and along with *Nfatc3*, is required for cardiac development and mitochondrial function (Bushdid et al. 2003). In silico analyses were employed to investigate binding sites of NFATC4 (human genome hg38 assembly). Data mined from ChIP-Atlas, a web service that integrates high-throughput chromatin immunoprecipitation sequencing (ChIP-seq) results archived at the Sequence Read Archive (Zou et al. 2022), indicated glutathione S-transferase alpha 4 (*GSTA4*) as a potential target gene of NFATC4. *GSTA4* plays an essential role in detoxification pathways by conjugating 4-hydroxynonenal and other products of lipid peroxidation to GSH for excretion (Hiratsuka et al. 2001). Using the UCSC Table Browser tool (Karolchik et al. 2004), we searched for the 5’-GGAAAAT-3’ sequence – a probable consensus binding site for NFATC4 (Stelzer et al. 2016, Yang et al. 2002) – in the promoter of *GSTA4*. We identified two instances of this sequence located at chr6:52,994,397 − 52,994,404 bp reverse strand and chr6:52,994,595 − 52,994,602 bp reverse strand in the *GSTA4* promoter. This result closely mirrors findings from a prior study which located two distinct *NFATC4* binding sequences in the PPARγ2 gene promoter (Yang et al. 2002). Overall, these data support *NFATC4* as a candidate gene underlying cardiac GSH, potentially through *GSTA4* regulation, and should be further scrutinized to define the precise role of this gene in cardiac redox function.

Although no other genes had phenotypic and functional annotations related to oxidative stress and the heart, a significant collection of T-cell receptor (TCR) genes was discovered underlying the cardiac GSH locus. TCRs play an important role in the immune system as they are responsible for binding antigens on the surface of T cells, facilitating pathogen clearance (Conley et al. 2016). The level of TCR engagement has been associated with ROS production: when TCRs are repetitively stimulated or presented with high amounts of antigen, ROS concentrations increase (Kesarwani et al. 2013, Hildeman et al. 2003). Given this phenomenon, several studies have investigated the impact of GSH on T-cell function and redox status. *GPx-1* deficient T helper cells exhibit increased intracellular ROS levels compared to wild-type T helper cells (Won et al. 2010). Furthermore, when cardiac endothelial cells from B6 mice are depleted of GSH, decreased T-cell proliferation and adhesion occur (McKenna et al. 2006), indicating that GSH depletion may impede T-cell function. In addition, T-regulatory cells were found to disrupt GSH metabolism in dendritic cells and T cells through multiple mechanisms, including decreasing *GCL* expression and inhibiting GSH redistribution from the nucleus to the cytoplasm (Kesarwani et al. 2013, Yan et al. 2010). Although it has been documented that TCR gene expression is tightly controlled in both mice and humans (Rodríguez-Caparrós et al. 2020), we did not find any prior studies examining the relationship between GSH levels and TCR gene expression. Future studies should explore the intricate relationship between GSH and T cells, specifically how altered TCR gene expression may influence GSH status. Given the compelling evidence for several candidate genes within this locus, additional studies are needed to determine the causative gene underlying cardiac GSH and its specific effects on cardiovascular health.

Notable in this study were the significant correlations identified between cardiac and renal GSH systems but not between cardiac and hepatic systems; initial analyses of the hepatic and renal GSH systems can be found in a separate study (Gould et al. 2021). We identified inverse correlations between cardiac GSH and renal GSH (**ρ** = -0.26, *p* < 0.001), renal GSSG (**ρ** = -0.17, *p* < 0.05) and renal total GSH (**ρ** = -0.27, *p* < 0.001). These findings contrast with a previous study of aged inbred mice, which reported positive correlations between cardiac and renal GSH concentrations (*r* = 0.69, *p* = 0.001) (Gould et al. 2018). Our study, however, captured the redox status of genetically diverse mice earlier in adulthood, which may explain the observed differences. We posit that the correlations identified here may be attributed to the shared physiological response of the heart and kidney under oxidative stress conditions. CVD and chronic kidney disease have been linked by several shared mechanisms, including oxidative stress, inflammation, and endothelial dysfunction (Ravarotto et al. 2022, Ravarotto et al. 2018). In fact, CVD is the leading cause of death in patients with end-stage renal disease (Global 2020). Under this assumption, the cardiac and renal glutathione systems should exhibit shared genetic regulation; however, we did not identify any overlapping loci between these systems. Future work should continue to explore the interplay and regulatory mechanisms governing the GSH system in cardiac and renal tissues.

Despite the strengths of this study, several limitations were present. First, due to the small size of the mouse heart, we needed to use the entire organ for biochemical analyses of GSH. Thus, we could not obtain other detailed heart measurements or perform histopathology. Future studies should separate mice into groups for GSH analysis and histopathological characterization. In addition, we observed low GSH levels in several samples, resulting in GSH/GSSG ratios below the expected values. While artefactual oxidation may have contributed to this effect, the overall impact was mitigated by applying rank-Z transformations in QTL analysis. Furthermore, while many of the QTL reported in this study exceeded the LOD score = 6 threshold convention, none of these peaks reached significance. Although 208 mice provided sufficient power for this investigation, we hypothesize that a greater sample size would result in QTL peaks meeting significance. Founder allele effects from BLUPs are therefore presented as hypothesis-generating. Finally, while this study was conducted in the genetically diverse DO stock containing over 37.8 million SNPs across its genome (Schmidt 2015, Churchill et al. 2012, Gatti et al. 2014), relevance of these findings to the human heart is not immediately certain.

In this study, we provide evidence for a novel QTL underlying cardiac GSH on chromosome 14 in a large cohort of genetically diverse mice. We present several candidate genes within this locus supported by public cardiac-specific expression, functional, and phenotypic annotations. Future studies will narrow down these candidates and delineate the mechanisms influencing GSH concentrations. Secondarily, we report correlations between the cardiac and renal GSH systems, distinct from any relationship with hepatic GSH. Through these efforts, we report novel genetic regulation of cardiac GSH and contribute to the goal of defining the complete genetic architecture of the glutathione redox system.

## Supplementary Information

Below is the link to the electronic supplementary material.

Supplementary Material 1

Supplementary Material 2

## Data Availability

All genotype data, genotype probabilities, and marker information are publicly available through figshare (https://figshare.com/collections/Pazdro-DO-Genotypes-R01-Glutathione-Redox-System/5360501/1). All source code, phenotype data, and other files used in QTL analyses are available through a public GitHub repository (https://github.com/smcclatchy/pazdro-glutathione-redox/tree/main).

## References

[CR1] Ahmad F, Seidman J, Seidman CE (2005) The genetic basis for cardiac remodeling. Annu Rev Genomics Hum Genet 6:185–216 16124859 10.1146/annurev.genom.6.080604.162132

[CR2] Andreadou I et al (2020) The role of mitochondrial reactive oxygen species, NO and H2S in ischaemia/reperfusion injury and cardioprotection. J Cell Mol Med 24(12):6510–6522 32383522 10.1111/jcmm.15279 PMC7299678

[CR3] Angelovski M et al (2023) Myocardial infarction and oxidative damage in animal models: objective and expectations from the application of cysteine derivatives. Toxicol Mech Methods 33(1):1–17 35450505 10.1080/15376516.2022.2069530

[CR4] Ardanaz N et al (2010) Lack of glutathione peroxidase 1 accelerates cardiac-specific hypertrophy and dysfunction in angiotensin II hypertension. Hypertension 55(1):116–123 19917877 10.1161/HYPERTENSIONAHA.109.135715 PMC3061336

[CR5] Ashburner M et al (2000) Gene ontology: tool for the unification of biology. Nat Genet 25(1):25–29 10802651 10.1038/75556 PMC3037419

[CR6] Baldarelli RM et al (2020) The mouse Gene Expression Database (GXD): 2021 update. Nucleic Acids Res 49(D1):D924–D931 10.1093/nar/gkaa914 PMC7778941 33104772

[CR7] Ballatori N et al (2009) Glutathione dysregulation and the etiology and progression of human diseases. Biol Chem 390(3):191–214 19166318 10.1515/BC.2009.033 PMC2756154

[CR8] Bikbov B et al (2020) Global, regional, and national burden of chronic kidney disease, 1990–2017: a systematic analysis for the global burden of disease study 2017. Lancet 395(10225):709–733 32061315 10.1016/S0140-6736(20)30045-3 PMC7049905

[CR9] Blake JA et al (2021) Mouse Genome Database (MGD): Knowledgebase for mouse-human comparative biology. Nucleic Acids Res 49(D1):D981–d987 33231642 10.1093/nar/gkaa1083 PMC7779030

[CR10] Blankenberg S et al (2003) Glutathione peroxidase 1 activity and cardiovascular events in patients with coronary artery disease. N Engl J Med 349(17):1605–1613 14573732 10.1056/NEJMoa030535

[CR11] Boyland E, Chasseaud L (1969) The role of glutathione and glutathione S-transferases in mercapturic acid biosynthesis. Adv Enzymol Relat Areas Mol Biol 32:173–219 4892500 10.1002/9780470122778.ch5

[CR12] Broman KW et al (2019) R/qtl2: software for mapping quantitative trait loci with high-dimensional data and multiparent populations. Genetics 211(2):495–502 30591514 10.1534/genetics.118.301595 PMC6366910

[CR13] Bushdid PB et al (2003) NFATc3 and NFATc4 Are Required for Cardiac Development and Mitochondrial Function. Circul Res 92(12):1305–1313 10.1161/01.RES.0000077045.84609.9F 12750314

[CR14] Callis TE et al (2009) MicroRNA-208a is a regulator of cardiac hypertrophy and conduction in mice. J Clin Investig 119(9):2772–2786 19726871 10.1172/JCI36154 PMC2735902

[CR15] Carniel E et al (2005) Alpha-myosin heavy chain: a sarcomeric gene associated with dilated and hypertrophic phenotypes of cardiomyopathy. Circulation 112(1):54–59 15998695 10.1161/CIRCULATIONAHA.104.507699

[CR16] Chen C-A et al (2010) -glutathionylation uncouples eNOS and regulates its cellular and vascular function. Nature 468(7327):1115–1118 21179168 10.1038/nature09599 PMC3370391

[CR17] Cheng M-l et al (2009) Effect of acute myocardial infarction on erythrocytic glutathione peroxidase 1 activity and plasma vitamin e levels. Am J Cardiol 103(4):471–475 19195504 10.1016/j.amjcard.2008.09.104

[CR18] Chien KR, Knowlton KU, Chien S (1991) Regulation of cardiac gene expression during myocardial growth and hypertrophy: molecular studies of an adaptive physiologic response. FASEB J 5(15):3037–3064 1835945 10.1096/fasebj.5.15.1835945

[CR19] Churchill GA et al (2012) The Diversity Outbred mouse population. Mamm Genome 23(9–10):713–718 22892839 10.1007/s00335-012-9414-2 PMC3524832

[CR20] Churchill GA, Doerge R (1994) Empirical threshold values for quantitative trait mapping. Genetics 138(3):963–971 7851788 10.1093/genetics/138.3.963 PMC1206241

[CR21] Conley JM, Gallagher MP, Berg LJ (2016) T cells and gene regulation: the switching on and turning up of genes after T cell receptor stimulation in CD8 T cells. Front Immunol 7:76 26973653 10.3389/fimmu.2016.00076 PMC4770016

[CR22] Consortium GO (2017) Expansion of the Gene Ontology knowledgebase and resources. Nucleic Acids Res 45(D1):D331–D338 27899567 10.1093/nar/gkw1108 PMC5210579

[CR24] Cui C et al (2024) HMDD v4.0: a database for experimentally supported human microRNA-disease associations. Nucleic Acids Res 52(D1):D1327–d1332 37650649 10.1093/nar/gkad717 PMC10767894

[CR25] Damy T et al (2009) Glutathione deficiency in cardiac patients is related to the functional status and structural cardiac abnormalities. PLoS ONE 4(3):e4871–e4871 19319187 10.1371/journal.pone.0004871 PMC2655715

[CR26] Espinola-Klein C et al (2007) Glutathione peroxidase-1 activity, atherosclerotic burden, and cardiovascular prognosis. Am J Cardiol 99(6):808–812 17350371 10.1016/j.amjcard.2006.10.041

[CR27] Espinosa-Díez C et al (2018) Role of glutathione biosynthesis in endothelial dysfunction and fibrosis. Redox Biol 14:88–99 28888203 10.1016/j.redox.2017.08.019 PMC5596265

[CR28] Ess M et al (2011) Gamma-glutamyltransferase rather than total bilirubin predicts outcome in chronic heart failure. J Card Fail 17(7):577–584 21703530 10.1016/j.cardfail.2011.02.012

[CR29] Forman HJ, Zhang H, Rinna A (2009) Glutathione: overview of its protective roles, measurement, and biosynthesis. Mol Aspects Med 30(1–2):1–12 18796312 10.1016/j.mam.2008.08.006 PMC2696075

[CR30] Gao J et al (2008) Glutathione peroxidase 1-deficient mice are more susceptible to doxorubicin-induced cardiotoxicity. Biochim Biophys Acta (BBA) - Mol Cell Res 1783(10):2020–2029 10.1016/j.bbamcr.2008.05.027 PMC2629733 18602426

[CR31] García-de-la-Asunción J et al (2016) Glutathione oxidation correlates with one-lung ventilation time and PO2/FiO2 ratio during pulmonary lobectomy. Redox Rep 21(5):219–226 26795138 10.1080/13510002.2015.1101890 PMC6837706

[CR32] Garfinkel AC, Seidman JG, Seidman CE (2018) Genetic pathogenesis of hypertrophic and dilated cardiomyopathy. Heart Fail Clin 14(2):139–146 29525643 10.1016/j.hfc.2017.12.004 PMC5851453

[CR33] M Gatti D et al (2014) Quantitative trait locus mapping methods for diversity outbred mice. G3 (Bethesda) 4(9):1623–1633 25237114 10.1534/g3.114.013748 PMC4169154

[CR34] Giustarini D et al (2004) -glutathionylation: from redox regulation of protein functions to human diseases. J Cell Mol Med 8(2):201–212 15256068 10.1111/j.1582-4934.2004.tb00275.x PMC6740303

[CR35] Gould RL et al (2018) Heritability of the aged glutathione phenotype is dependent on tissue of origin. Mamm Genome 29:619–631 30008145 10.1007/s00335-018-9759-2

[CR36] Gould RL et al (2021) Quantitative trait mapping in Diversity Outbred mice identifies novel genomic regions associated with the hepatic glutathione redox system. Redox Biol 46:102093 34418604 10.1016/j.redox.2021.102093 PMC8385155

[CR37] Gould RL et al (2021) Genetic mapping of renal glutathione suggests a novel regulatory locus on the murine X chromosome and overlap with hepatic glutathione regulation. Free Radic Biol Med 174:28–39 34324982 10.1016/j.freeradbiomed.2021.07.035 PMC8597656

[CR38] Graef IA, Chen F, Crabtree GR (2001) NFAT signaling in vertebrate development. Curr Opin Genet Dev 11(5):505–512 11532391 10.1016/s0959-437x(00)00225-2

[CR39] Granados-Riveron JT et al (2010) α-Cardiac myosin heavy chain (MYH6) mutations affecting myofibril formation are associated with congenital heart defects. Hum Mol Genet 19(20):4007–4016 20656787 10.1093/hmg/ddq315

[CR40] Griendling KK et al (2016) Measurement of reactive oxygen species, reactive nitrogen species, and redox-dependent signaling in the cardiovascular system: a scientific statement from the American Heart Association. Circul Res 119(5):e39–e75 10.1161/RES.0000000000000110 PMC5446086 27418630

[CR41] Gupta MP (2007) Factors controlling cardiac myosin-isoform shift during hypertrophy and heart failure. J Mol Cell Cardiol 43(4):388–403 17720186 10.1016/j.yjmcc.2007.07.045 PMC2701247

[CR42] Hamanishi T et al (2004) Functional Variants in the Glutathione Peroxidase-1 (GPx-1) Gene Are Associated With Increased Intima-Media Thickness of Carotid Arteries and Risk of Macrovascular Diseases in Japanese Type 2 Diabetic Patients. Diabetes 53(9):2455–2460 15331559 10.2337/diabetes.53.9.2455

[CR43] Hildeman DA et al (2003) T cell apoptosis and reactive oxygen species. J Clin Investig 111(5):575–581 12618509 10.1172/JCI18007 PMC151907

[CR44] Hiratsuka A et al (2001) (S)-preferential detoxification of 4-hydroxy-2(E)-nonenal enantiomers by hepatic glutathione S-transferase isoforms in guinea-pigs and rats. Biochem J 355(Pt 1):237–244 11256969 10.1042/0264-6021:3550237 PMC1221732

[CR45] Huang S et al (2021) Novel insertion mutation (Arg1822_Glu1823dup) in MYH6 coiled-coil domain causing familial atrial septal defect. Eur J Med Genet 64(11):104314 34481090 10.1016/j.ejmg.2021.104314

[CR46] Ikeda S et al (2009) MicroRNA-1 negatively regulates expression of the hypertrophy-associated calmodulin and Mef2a genes. Mol Cell Biol 29(8):2193–2204 19188439 10.1128/MCB.01222-08 PMC2663304

[CR47] Jiang S, Jiang D, Tao Y (2013) Role of gamma-glutamyltransferase in cardiovascular diseases. Exp Clin Cardiol 18(1):53–56 24294039 PMC3716505

[CR48] Julius M et al (1994) Glutathione and morbidity in a community-based sample of elderly. J Clin Epidemiol 47(9):1021–1026 7730904 10.1016/0895-4356(94)90117-1

[CR49] Kaplowitz N, Ookhtens AT (1985) The regulation of hepatic GSH. Ann Rev Pharm Toxicol 25:714–744 10.1146/annurev.pa.25.040185.003435 3890714

[CR50] Karolchik D (2004) The UCSC Table Browser data retrieval tool. Nucleic Acids Res 32(Database issue):D493-6 14681465 10.1093/nar/gkh103 PMC308837

[CR51] Keane TM et al (2011) Mouse genomic variation and its effect on phenotypes and gene regulation. Nature 477(7364):289–294 21921910 10.1038/nature10413 PMC3276836

[CR52] Kesarwani P et al (2013) Redox regulation of T-cell function: from molecular mechanisms to significance in human health and disease. Antioxid Redox Signal 18(12):1497–1534 22938635 10.1089/ars.2011.4073 PMC3603502

[CR53] Khazim K et al (2013) Glutathione redox potential is low and glutathionylated and cysteinylated hemoglobin levels are elevated in maintenance hemodialysis patients. Translational Res 162(1):16–25 10.1016/j.trsl.2012.12.014 PMC3683567 23333585

[CR54] Kinsella RJ et al (2011) Ensembl BioMarts: a hub for data retrieval across taxonomic space. Database. 10.1093/database/bar030 10.1093/database/bar030 PMC3170168 21785142

[CR55] Kinsella RJ et al (2011) Ensembl BioMarts: a hub for data retrieval across taxonomic space. Database (Oxford) 2011:bar030 21785142 10.1093/database/bar030 PMC3170168

[CR56] Klaassen S et al (2008) Mutations in sarcomere protein genes in left ventricular noncompaction. Circulation 117(22):2893–2901 18506004 10.1161/CIRCULATIONAHA.107.746164

[CR57] Klos M et al (2017) Altered myocyte contractility and calcium homeostasis in alpha-myosin heavy chain point mutations linked to familial dilated cardiomyopathy. Arch Biochem Biophys 615:53–60 28088328 10.1016/j.abb.2016.12.007

[CR58] Koide S-i et al (2003) Association of polymorphism in glutamate-cysteine ligase catalytic subunit gene with coronary vasomotor dysfunction and myocardial infarction. J Am Coll Cardiol 41(4):539–545 12598062 10.1016/s0735-1097(02)02866-8

[CR59] Kraft T et al (2016) Hypertrophic cardiomyopathy: cell-to-cell imbalance in gene expression and contraction force as trigger for disease phenotype development. Circul Res 119(9):992–995 10.1161/CIRCRESAHA.116.309804 27737944

[CR60] Kura B et al (2020) Oxidative Stress-Responsive MicroRNAs in Heart Injury. Int J Mol Sci. 10.3390/ijms21010358 10.3390/ijms21010358 PMC6981696 31948131

[CR61] Lapenna D et al (1998) Glutathione-related antioxidant defenses in human atherosclerotic plaques. Circulation 97(19):1930–1934 9609086 10.1161/01.cir.97.19.1930

[CR62] Lee EJ et al (2023) The Protective Role of Glutathione against Doxorubicin-Induced Cardiotoxicity in Human Cardiac Progenitor Cells. Int J Mol Sci. 10.3390/ijms241512070 10.3390/ijms241512070 PMC10419046 37569446

[CR63] Li Z et al (2018) *SIRT6 suppresses NFATc4 expression and activation in cardiomyocyte hypertrophy.* Front Pharmacol 9:1519 30670969 10.3389/fphar.2018.01519 PMC6331469

[CR64] Li M et al (2016) *NFATc4 and myocardin synergistically up-regulate the expression of LTCC α1C in ET-1-induced cardiomyocyte hypertrophy.* Life Sci 155:11–20 27155398 10.1016/j.lfs.2016.05.007

[CR65] Liu J, Wang DZ (2014) An epigenetic LINK(RNA) to pathological cardiac hypertrophy. Cell Metab 20(4):555–557 25295782 10.1016/j.cmet.2014.09.011 PMC4886471

[CR66] Liu X-P et al (2015) Peroxisome proliferator–activated receptor gamma coactivator 1 alpha protects cardiomyocytes from hypertrophy by suppressing calcineurin-nuclear factor of activated T cells c4 signaling pathway. Translational Res 166(5):459–473e3 10.1016/j.trsl.2015.06.003 26118953

[CR67] Lu SC (2009) Regulation of glutathione synthesis. Mol Aspects Med 30(1–2):42–59 18601945 10.1016/j.mam.2008.05.005 PMC2704241

[CR68] Lushchak VI (2012) Glutathione homeostasis and functions: potential targets for medical interventions. J Amino Acids 2012:p736837 10.1155/2012/736837 PMC3303626 22500213

[CR69] Magrì D et al (2020) Risk stratification in hypertrophic cardiomyopathy. insights from genetic analysis and cardiopulmonary exercise testing. J Clin Med 9(6):1636 32481709 10.3390/jcm9061636 PMC7356142

[CR70] Makarewicz-Wujec M, Kozlowska-Wojciechowska M (2011) Nutrient intake and serum level of gamma-glutamyltransferase, MCP-1 and homocysteine in early stages of heart failure. Clin Nutr 30(1):73–78 20708308 10.1016/j.clnu.2010.07.008

[CR71] Masella R et al (2005) Novel mechanisms of natural antioxidant compounds in biological systems: involvement of glutathione and glutathione-related enzymes. J Nutr Biochem 16(10):577–586 16111877 10.1016/j.jnutbio.2005.05.013

[CR72] Matuz-Mares D et al (2021) Glutathione Participation in the Prevention of Cardiovascular Diseases. Antioxidants. 10.3390/antiox10081220 34439468 10.3390/antiox10081220 PMC8389000

[CR73] McKenna GJ et al (2006) Glutathione depletion of stimulator cells inhibits responder T-cell immunogenicity in vitro and prolongs allograft survival in vivo. Am J Surg 191(5):588–592 16647342 10.1016/j.amjsurg.2006.02.006

[CR74] Mekala N et al (2021) MiR 208a Regulates Mitochondrial Biogenesis in Metabolically Challenged Cardiomyocytes. Cells 10(11):3152 34831374 10.3390/cells10113152 PMC8622724

[CR75] Mohammadi A et al (2021) Evaluation of Oxidative Stress, Apoptosis, and Expression of MicroRNA-208a and MicroRNA-1 in Cardiovascular Patients. Rep Biochem Mol Biol 10(2):183–196 34604408 10.52547/rbmb.10.2.183 PMC8480300

[CR76] Molkentin JD (2000) Calcineurin and beyond: cardiac hypertrophic signaling. Circ Res 87(9):731–738 11055975 10.1161/01.res.87.9.731

[CR77] Molkentin JD et al (1998) A calcineurin-dependent transcriptional pathway for cardiac hypertrophy. Cell 93(2):215–228 9568714 10.1016/s0092-8674(00)81573-1 PMC4459646

[CR78] Morgan AP et al (2016) The mouse universal genotyping array: from substrains to subspecies. G3 Genes, Genomes, Genetics 6(2):263–279 10.1534/g3.115.022087 PMC4751547 26684931

[CR79] Moris D et al (2017) The role of reactive oxygen species in the pathophysiology of cardiovascular diseases and the clinical significance of myocardial redox. Annals of translational medicine. 10.21037/atm.2017.06.27 10.21037/atm.2017.06.27 PMC5566734 28861423

[CR80] Morkin E (2000) Control of cardiac myosin heavy chain gene expression. Microsc Res Tech 50(6):522–531 10998641 10.1002/1097-0029(20000915)50:6<522::AID-JEMT9>3.0.CO;2-U

[CR81] Nadal-Ginard B, Mahdavi V (1989) Molecular basis of cardiac performance. Plasticity of the myocardium generated through protein isoform switches. J Clin Invest 84(6):1693–1700 2687327 10.1172/JCI114351 PMC304044

[CR82] Nakamura S-i et al (2002) Polymorphism in the 5′-Flanking Region of Human Glutamate-Cysteine Ligase Modifier Subunit Gene Is Associated With Myocardial Infarction. Circulation 105(25):2968–2973 12081989 10.1161/01.cir.0000019739.66514.1e

[CR83] Nakamura S-i et al (2003) Polymorphism in Glutamate-Cysteine Ligase Modifier Subunit Gene Is Associated With Impairment of Nitric Oxide–Mediated Coronary Vasomotor Function. Circulation 108(12):1425–1427 12975258 10.1161/01.CIR.0000091255.63645.98

[CR84] O’Brien J et al (2018) Overview of MicroRNA Biogenesis, Mechanisms of Actions, and Circulation. Frontiers in Endocrinology. 10.3389/fendo.2018.00402 10.3389/fendo.2018.00402 PMC6085463 30123182

[CR85] Pandya K et al (2010) Reversible epigenetic modifications of the two cardiac myosin heavy chain genes during changes in expression. Gene Expr 15(2):51–59 21526716 10.3727/105221611x12973615737505 PMC3243912

[CR86] Paolicchi A et al (1999) Gamma-glutamyl transpeptidase-dependent iron reduction and LDL oxidation–a potential mechanism in atherosclerosis. J Invest medicine: official publication Am Federation Clin Res 47(3):151–160 10198571

[CR87] Park HJ, Mah E, Bruno RS (2010) Validation of high-performance liquid chromatography–boron-doped diamond detection for assessing hepatic glutathione redox status. Anal Biochem 407(2):151–159 20705049 10.1016/j.ab.2010.08.012

[CR88] Pisoschi AM, Pop A (2015) The role of antioxidants in the chemistry of oxidative stress: a review. Eur J Med Chem 97:55–74 25942353 10.1016/j.ejmech.2015.04.040

[CR89] Pompella A et al (2004) The significance of serum γ-glutamyltransferase in cardiovascular diseases. Clin Chem Lab Med (CCLM) 42(10):1085–1091 15552264 10.1515/CCLM.2004.224

[CR90] Ravarotto V et al (2022) Pathomechanism of oxidative stress in cardiovascularrenal remodeling and therapeutic strategies. Kidney Res Clin Pract 41(5):533–544 36239057 10.23876/j.krcp.22.069 PMC9576462

[CR91] Ravarotto V et al (2018) Oxidative stress - chronic kidney disease - cardiovascular disease: A vicious circle. Life Sci 210:125–131 30172705 10.1016/j.lfs.2018.08.067

[CR92] Rawal S et al (2019) Early dysregulation of cardiac-specific microRNA-208a is linked to maladaptive cardiac remodelling in diabetic myocardium. Cardiovasc Diabetol 18(1):13 30696455 10.1186/s12933-019-0814-4 PMC6352455

[CR93] Rebrin I, Kamzalov S, Sohal RS (2003) Effects of age and caloric restriction on glutathione redox state in mice. Free Radic Biol Med 35(6):626–635 12957655 10.1016/s0891-5849(03)00388-5 PMC2837076

[CR94] Recla JM et al (2019) Genetic mapping in Diversity Outbred mice identifies a Trpa1 variant influencing late-phase formalin response. Pain 160(8):1740 31335644 10.1097/j.pain.0000000000001571 PMC6668363

[CR95] Recla JM et al (2014) Precise genetic mapping and integrative bioinformatics in Diversity Outbred mice reveals Hydin as a novel pain gene. Mamm Genome 25:211–222 24700285 10.1007/s00335-014-9508-0 PMC4032469

[CR96] Robinson GK (1991) That BLUP is a good thing: the estimation of random effects. Statistical science. 10.1214/ss/1177011926

[CR97] Rodríguez-Caparrós A et al (2020) Regulation of T-cell Receptor Gene Expression by Three-Dimensional Locus Conformation and Enhancer Function. Int J Mol Sci. 10.3390/ijms21228478 10.3390/ijms21228478 PMC7696796 33187197

[CR98] Rost J, Rapoport S (1964) Reduction-potential of glutathione. Nature 201:185–185 10.1038/201185a0 14118271

[CR99] Rotoli BM et al (2018) Downregulation of SLC7A7 Triggers an Inflammatory Phenotype in Human Macrophages and Airway Epithelial Cells. Front Immunol 9:508 29616026 10.3389/fimmu.2018.00508 PMC5868322

[CR100] Ruttmann E et al (2005) γ-Glutamyltransferase as a Risk Factor for Cardiovascular Disease Mortality. Circulation 112(14):2130–2137 16186419 10.1161/CIRCULATIONAHA.105.552547

[CR102] Schmidt CW (2015) Diversity outbred: a new generation of mouse model. Environ Health Perspect 123(3):A64–A67 25730842 10.1289/ehp.123-A64 PMC4348734

[CR103] Schnabel R et al (2005) Glutathione peroxidase-1 and homocysteine for cardiovascular risk prediction: results from the athero gene study. J Am Coll Cardiol 45(10):1631–1637 15893179 10.1016/j.jacc.2005.02.053

[CR104] Schriml LM et al (2022) The Human Disease Ontology 2022 update. Nucleic Acids Res 50(D1):D1255–d1261 34755882 10.1093/nar/gkab1063 PMC8728220

[CR105] Sen Ś, Churchill GA (2001) A statistical framework for quantitative trait mapping. Genetics 159(1):371–387 11560912 10.1093/genetics/159.1.371 PMC1461799

[CR106] Shanmugam G et al (2020) Reductive Stress Causes Pathological Cardiac Remodeling and Diastolic Dysfunction. Antioxid Redox Signal 32(18):1293–1312 32064894 10.1089/ars.2019.7808 PMC7247052

[CR107] Shi Z-Z et al (2000) Glutathione synthesis is essential for mouse development but not for cell growth in culture. Proc Nat Acad Sci 97(10):5101–5106 10805773 10.1073/pnas.97.10.5101 PMC25788

[CR108] Shimizu H et al (2004) Relationship between plasma glutathione levels and cardiovascular disease in a defined population: the Hisayama study. Stroke 35(9):2072–2077 15256685 10.1161/01.STR.0000138022.86509.2d

[CR110] Smith CL, Goldsmith C-AW, Eppig JT (2004) The Mammalian Phenotype Ontology as a tool for annotating, analyzing and comparing phenotypic information. Genome Biol 6(1):R7 15642099 10.1186/gb-2004-6-1-r7 PMC549068

[CR111] Souiden Y et al (2016) MnSOD and GPx1 polymorphism relationship with coronary heart disease risk and severity. Biol Res 49:22 27067415 10.1186/s40659-016-0083-6 PMC4828869

[CR113] Stelzer G et al (2016) The GeneCards Suite: From Gene Data Mining to Disease Genome Sequence Analyses. Curr Protoc Bioinf 54:p1301–p13033 10.1002/cpbi.5 27322403

[CR114] Svenson KL et al (2012) High-resolution genetic mapping using the Mouse Diversity outbred population. Genetics 190(2):437–447 22345611 10.1534/genetics.111.132597 PMC3276626

[CR115] Tang N-P et al (2008) Genetic variant in glutathione peroxidase 1 gene is associated with an increased risk of coronary artery disease in a Chinese population. Clin Chim Acta 395(1–2):89–93 18541150 10.1016/j.cca.2008.05.013

[CR116] Thakur M et al (2022) EMBL’s European Bioinformatics Institute (EMBL-EBI) in 2022. Nucleic Acids Res 51(D1):D9–D17 10.1093/nar/gkac1098 PMC9825486 36477213

[CR117] Theis JL et al (2015) Recessive MYH6 Mutations in Hypoplastic Left Heart With Reduced Ejection Fraction. Circ Cardiovasc Genet 8(4):564–571 26085007 10.1161/CIRCGENETICS.115.001070

[CR118] Tomita-Mitchell A et al (2016) Impact of MYH6 variants in hypoplastic left heart syndrome. Physiol Genomics 48(12):912–921 27789736 10.1152/physiolgenomics.00091.2016 PMC5206387

[CR119] Van Rooij E et al (2006) A signature pattern of stress-responsive microRNAs that can evoke cardiac hypertrophy and heart failure. Proc Natl Acad Sci 103(48):18255–18260 17108080 10.1073/pnas.0608791103 PMC1838739

[CR120] van Rooij E et al (2007) Control of Stress-Dependent Cardiac Growth and Gene Expression by a MicroRNA. Science 316(5824):575–579 17379774 10.1126/science.1139089

[CR121] Velicki L et al (2020) Genetic determinants of clinical phenotype in hypertrophic cardiomyopathy. BMC Cardiovasc Disord 20(1):516 33297970 10.1186/s12872-020-01807-4 PMC7727200

[CR122] Wang L et al (2022) Targeting endothelial dysfunction and inflammation. J Mol Cell Cardiol 168:58–67 35460762 10.1016/j.yjmcc.2022.04.011

[CR123] Wang X et al (1993) Intramucosal pH and oxygen extraction in the gastrointestinal tract after major liver resection in rats. Eur J Surgery= Acta Chir 159(2):81–87 8098631

[CR124] Warkman AS et al (2012) Developmental expression and cardiac transcriptional regulation of Myh7b, a third myosin heavy chain in the vertebrate heart. Cytoskeleton (Hoboken) 69(5):324–335 22422726 10.1002/cm.21029 PMC4734749

[CR125] Weil BR et al (2011) Prehypertension Is Associated With Impaired Nitric Oxide-Mediated Endothelium-Dependent Vasodilation in Sedentary Adults. Am J Hypertens 24(9):976–981 21633396 10.1038/ajh.2011.88

[CR126] Won HY et al (2010) Glutathione peroxidase 1 deficiency attenuates allergen-induced airway inflammation by suppressing Th2 and Th17 cell development. Antioxid redox signal 13(5):575–587 20367278 10.1089/ars.2009.2989

[CR127] Wu G et al (2004) Glutathione metabolism and its implications for health. J Nutr 134(3):489–492 14988435 10.1093/jn/134.3.489

[CR128] Xia Y et al (2019) Novel Mutation in MYH6 in 2 Unrelated Chinese Han Families With Familial Atrial Septal Defect. Circ Genom Precis Med 12(11):e002732 31638415 10.1161/CIRCGEN.119.002732

[CR129] Yan Z, Garg SK, Banerjee R (2010) Regulatory T cells interfere with glutathione metabolism in dendritic cells and T cells. J Biol Chem 285(53):41525–41532 21037289 10.1074/jbc.M110.189944 PMC3009879

[CR130] Yang J et al (2014) Advantages and pitfalls in the application of mixed-model association methods. Nat Genet 46(2):100–106 24473328 10.1038/ng.2876 PMC3989144

[CR131] Yang TT et al (2002) Phosphorylation of NFATc4 by p38 mitogen-activated protein kinases. Mol Cell Biol 22(11):3892–3904 11997522 10.1128/MCB.22.11.3892-3904.2002 PMC133816

[CR132] Yuan H et al (2019) Effects of microRNA-208a on inflammation and oxidative stress in ketamine-induced cardiotoxicity through Notch/NF-κB signal pathways by CHD9. Biosci Rep. 10.1042/BSR20182381 10.1042/BSR20182381 PMC6522736 30923228

[CR133] Zhang JX et al (2014) Association of glutathione peroxidase-1 (GPx-1) rs1050450 Pro198Leu and Pro197Leu polymorphisms with cardiovascular risk: a meta-analysis of observational studies. J Geriatr Cardiol 11(2):141–150 25009565 10.3969/j.issn.1671-5411.2014.02.003 PMC4076455

[CR134] Zhao Y et al (2007) Dysregulation of cardiogenesis, cardiac conduction, and cell cycle in mice lacking miRNA-1-2. Cell 129(2):303–317 17397913 10.1016/j.cell.2007.03.030

[CR135] Zhou Y et al (2014) Genetic analysis of tissue glutathione concentrations and redox balance. Free Radic Biol Med 71:157–164 24613380 10.1016/j.freeradbiomed.2014.02.027 PMC4043295

[CR136] Ziegler TR et al (2001) Regulation of glutathione redox status in lung and liver by conditioning regimens and keratinocyte growth factor in murine allogeneic bone marrow transplantation1. Transplantation 72(8):1354–1362 11685103 10.1097/00007890-200110270-00004

[CR137] Zou Z et al (2022) ChIP-Atlas 2021 update: a data-mining suite for exploring epigenomic landscapes by fully integrating ChIP-seq, ATAC-seq and Bisulfite-seq data. Nucleic Acids Res 50(W1):W175–W182 35325188 10.1093/nar/gkac199 PMC9252733

[CR138] Zuo JY et al (2022) Identification and functional analysis of variants of MYH6 gene promoter in isolated ventricular septal defects. BMC Med Genomics 15(1):213 36209093 10.1186/s12920-022-01365-y PMC9548206

[CR139] Zuzak E et al (2017) Glutathione level and glutathione reductase activity in serum of coronary heart disease patients. J Pre-Clinical Clin Res 11(2):103–105

