# Supplementary material for: Contrasting the genetic architecture of cardiac glutathione against other organs: unveiling a unique tissue-specific locus
Source: Mamm Genome. 2026 May 11;37(1):67. doi: 10.1007/s00335-026-10222-7 (PMC13160963; doi:10.1007/s00335-026-10222-7)
Supplement: Supplementary file 1 — Supplementary Material 1 [file 335_2026_10222_MOESM1_ESM.docx]

**SUPPLEMENTARY FILE**

**Supplementary Table S1.** **Databases queried for biological annotations for candidate gene prioritization.**

| **Database (Abbreviation)** | **Annotation Type** | **URL** | **Reference** |
| --- | --- | --- | --- |
| EBI Expression Atlas (EEA) | Expression | <https://www.ebi.ac.uk/gxa/home/> | [1] |
| Gene eXpression Database (GXD) | Expression | <http://www.informatics.jax.org/expression.shtml> | [2] |
| Gene Ontology (GO) | Functional | <http://www.geneontology.org/> | [3] |
| Mammalian Phenotype | Phenotypic | <https://www.informatics.jax.org/vocab/mp_ontology> | [4] |
| Disease Ontology (DO) | Phenotypic | <https://disease-ontology.org/do> | [5] |

**Supplementary Table S2. Descriptive statistics for cardiac GSH concentrations and redox status in male mice.**

Cardiac concentrations of GSH (nmol/mg protein) and GSSG (nmol/mg protein) were quantified in samples collected from DO mice (100 males) by HPLC. Total glutathione concentrations were then calculated (GSH + 2GSSG), as were GSH/GSSG and E_h_ (mV).

| **Phenotype** | **N** | **x̄** | **Median** | **SD** | **Min** | **Max** |
| --- | --- | --- | --- | --- | --- | --- |
| Total Glutathione (nmol/mg) | 100 | 8.49 | 7.71 | 3.27 | 3.72 | 20.41 |
| GSH (nmol/mg) | 100 | 4.32 | 3.31 | 3.49 | 0.0007 | 15.99 |
| GSSG (nmol/mg) | 100 | 2.08 | 1.98 | 0.78 | 1.02 | 5.80 |
| GSH/GSSG | 100 | 2.54 | 1.84 | 2.30 | 0.002 | 11.25 |
| E_h_ (mV) | 100 | -282.41 | -289.45 | 35.96 | -333.91 | -65.15 |

**Supplementary Table S3. Descriptive statistics for cardiac GSH concentrations and redox status in female mice.**

Cardiac concentrations of GSH (nmol/mg protein) and GSSG (nmol/mg protein) were quantified in samples collected from DO mice (108 females) by HPLC. Total glutathione concentrations were then calculated (GSH + 2GSSG), as were GSH/GSSG and E_h_ (mV).

| **Phenotype** | **N** | **x̄** | **Median** | **SD** | **Min** | **Max** |
| --- | --- | --- | --- | --- | --- | --- |
| Total Glutathione (nmol/mg) | 108 | 7.71 | 7.48 | 2.24 | 3.78 | 14.01 |
| GSH (nmol/mg) | 108 | 3.64 | 3.17 | 2.22 | 0.06 | 10.92 |
| GSSG (nmol/mg) | 108 | 2.03 | 1.92 | 0.60 | 1.02 | 3.48 |
| GSH/GSSG | 108 | 2.05 | 1.60 | 1.66 | 0.02 | 9.54 |
| E_h_ (mV) | 108 | -283.37 | -285.16 | 23.95 | -326.55 | -172.10 |

**Supplementary Table S4. Statistical correlations between markers of the cardiac GSH system.**

Spearman’s rho (ρ) was calculated to understand relationships between cardiac GSH system markers. Cardiac GSH and GSSG concentrations were calculated using HPLC and standardized as nmol/mg protein. Concentrations were then used to calculate total glutathione (GSH + 2GSSG), GSH/GSSG, and E_h_ (expressed as mV).

| **Phenotype** | | **ρ** | **p-value** |
| --- | --- | --- | --- |
| GSH | GSSG | -0.39 | p < 0.001 |
| GSH | Total Glutathione | 0.88 | p < 0.001 |
| GSSG | Total Glutathione | 0.16 | p < 0.05 |
| GSH | GSH/GSSG | 0.94 | p < 0.001 |
| GSSG | GSH/GSSG | -0.65 | p < 0.001 |
| Total Glutathione | GSH/GSSG | 0.59 | p < 0.001 |
| GSH | E_h_ | -0.98 | p < 0.001 |
| GSSG | E_h_ | 0.54 | p < 0.001 |
| Total Glutathione | E_h_ | -0.69 | p < 0.001 |
| GSH/GSSG | E_h_ | -0.99 | p < 0.001 |

**Supplementary Table S5. Results of high-resolution association mapping of cardiac GSH redox system.**

QTL peaks with LOD scores > 6 included within the table. Significant QTL position and respective Bayesian credible interval included in parentheses in Mbp. Total glutathione, GSH, and GSSG standardized to nmol/mg. E_h_ expressed in mV.

| **Phenotype** | **Chr** | **QTL Position (Mbp)** | **LOD** | **Genome-wide p-value** |
| --- | --- | --- | --- | --- |
| GSH | 14 | 54.240 (48.909 - 55.303) | 7.492 | 0.0905 |
| E_h_ | 14 | 54.239 (48.905 - 55.226) | 6.969 | 0.2072 |
| GSH | 16 | 96.736 (96.670 - 98.097) | 6.969 | 0.2076 |
| GSH | 19 | 57.209 (57.981 - 57.523) | 6.596 | 0.3455 |
| E_h_ | 16 | 96.736 (96.670 - 98.097) | 6.574 | 0.3555 |
| GSSG | 10 | 124.104 (87.902 - 125.347) | 6.411 | 0.4318 |
| GSH/GSSG | 14 | 54.239 (26.536 - 80.310) | 6.154 | 0.5654 |
| E_h_ | 19 | 57.209 (3.2638 - 57.978) | 6.147 | 0.5689 |
| GSH/GSSG | 5 | 38.839 (37.152 - 122.875) | 6.104 | 0.5923 |

**Supplementary Table S6. Chr 16 GSH candidate genes and their relevant annotations (Chr16:96.670-98.097±1 Mbp; GRCm38/mm10)^a^.**

^a^Resource abbreviations: **EEA,** EBI Expression Atlas; **DO,** Disease Ontology; **GO,** Gene Ontology; **GXD,** Gene eXpression Database; **MGI,** Mouse Genome Informatics; **MP,** Mammalian Phenotype.

|  |  |  |  | **Expression** | | **Functional** | **Phenotypic (cardiac-related)** |  |
| --- | --- | --- | --- | --- | --- | --- | --- | --- |
| **MGI Gene/Marker ID** | **Symbol** | **Feature Type** | **Ensembl ID** | **MGI** | **EEA** | **GO** | **MP** | **Human** |
| MGI:1310000 | Kcnj15 | protein coding gene | ENSMUSG00000062609 | x |  |  |  |  |
| MGI:95415 | Erg | protein coding gene | ENSMUSG00000040732 | x |  | x | x |  |
| MGI:95456 | Ets2 | protein coding gene | ENSMUSG00000022895 | x |  |  |  |  |
| MGI:1860263 | Psmg1 | protein coding gene | ENSMUSG00000022913 | x |  |  |  |  |
| MGI:1890651 | Brwd1 | protein coding gene | ENSMUSG00000022914 | x |  |  |  |  |
| MGI:96120 | Hmgn1 | protein coding gene | ENSMUSG00000040681 | x |  | x |  |  |
| MGI:2136882 | Get1 | protein coding gene | ENSMUSG00000023147 | x |  |  |  |  |
| MGI:1354740 | Sh3bgr | protein coding gene | ENSMUSG00000040666 | x |  |  |  |  |
| MGI:1354740 | Sh3bgr | protein coding gene | ENSMUSG00000040666 | x |  |  |  |  |
| MGI:97509 | Pcp4 | protein coding gene | ENSMUSG00000090223 | x |  |  |  |  |
| MGI:1860440 | Bace2 | protein coding gene | ENSMUSG00000040605 | x | x |  |  |  |
| MGI:1930121 | Prdm15 | protein coding gene | ENSMUSG00000014039 | x |  |  |  |  |
| MGI:1891883 | C2cd2 | protein coding gene | ENSMUSG00000045975 | x |  |  |  |  |
| MGI:1196281 | Dscam | protein coding gene | ENSMUSG00000050272 |  | x |  |  |  |
| MGI:97243 | Mx1 | polymorphic pseudogene | ENSMUSG00000000386 |  | x |  |  |  |

|  |  |  |  |  | Expression | | Functional | Phenotypic (cardiac-related) | |
| --- | --- | --- | --- | --- | --- | --- | --- | --- | --- |
| MGI Gene/Marker ID | **Symbol** | **Name** | **Feature Type** | **Ensembl ID** | **MGI** | **EEA** | **GO** | **MP** | **Human** |
| MGI:1098765 | Nrap | nebulin-related anchoring protein | protein coding gene | ENSMUSG00000049134 | x |  | x |  |  |
| MGI:87937 | Adrb1 | adrenergic receptor, beta 1 | protein coding gene | ENSMUSG00000035283 | x |  | x | x |  |
| MGI:1194500 | Ablim1 | actin-binding LIM protein 1 | protein coding gene | ENSMUSG00000025085 | x | x |  |  |  |
| MGI:2147749 | Atrnl1 | attractin like 1 | protein coding gene | ENSMUSG00000054843 | x |  |  |  |  |

**Supplementary Table S7. Chr 19 GSH candidate genes and their relevant annotations (Chr19:3.403-57.881±1 Mbp; GRCm38/mm10)^a^.**

^a^Resource abbreviations: **EEA,** EBI Expression Atlas; **DO,** Disease Ontology; **GO,** Gene Ontology; **GXD,** Gene eXpression Database; **MGI,** Mouse Genome Informatics; **MP,** Mammalian Phenotype.

**Supplementary Table S8. Chr 10 GSSG candidate genes and their relevant annotations (Chr10:87.902-125.347±1 Mbp; GRCm38/mm10)^a^.**

^a^Resource abbreviations: **EEA,** EBI Expression Atlas; **DO,** Disease Ontology; **GO,** Gene Ontology; **GXD,** Gene eXpression Database; **MGI,** Mouse Genome Informatics; **MP,** Mammalian Phenotype.

|  |  |  |  | **Expression** | | **Functional** | **Phenotypic (cardiac-related)** | |
| --- | --- | --- | --- | --- | --- | --- | --- | --- |
| **MGI Gene/Marker ID** | **Symbol** | **Feature Type** | **Ensembl ID** | **MGI** | **EEA** | **GO** | **MP** | **Human** |
| MGI:1922567 | Parpbp | protein coding gene | ENSMUSG00000035365 | **x** | **x** |  |  |  |
| MGI:1919964 | Nup37 | protein coding gene | ENSMUSG00000035351 | **x** | **x** |  |  |  |
| MGI:2384841 | Chpt1 | protein coding gene | ENSMUSG00000060002 | **x** | **x** |  |  |  |
| MGI:1918780 | Gas2l3 | protein coding gene | ENSMUSG00000074802 | **x** | **x** |  |  |  |
| MGI:1916252 | 1500026H17Rik | lncRNA gene | ENSMUSG00000097383 | **x** | **x** |  |  |  |
| MGI:1306796 | Apaf1 | protein coding gene | ENSMUSG00000019979 | **x** | **x** | **x** |  |  |
| MGI:1353498 | Slc25a3 | protein coding gene | ENSMUSG00000061904 | **x** | **x** |  |  |  |
| MGI:106920 | Tmpo | protein coding gene | ENSMUSG00000019961 | **x** | **x** |  |  |  |
| MGI:97293 | Nedd1 | protein coding gene | ENSMUSG00000019988 | **x** | **x** |  |  |  |
| MGI:96836 | Lta4h | protein coding gene | ENSMUSG00000015889 | **x** | **x** |  |  |  |
| MGI:1888978 | Ntn4 | protein coding gene | ENSMUSG00000020019 | **x** | **x** |  |  |  |
| MGI:2143698 | Vezt | protein coding gene | ENSMUSG00000036099 | **x** | **x** |  |  |  |
| MGI:1336168 | Cradd | protein coding gene | ENSMUSG00000045867 | **x** | **x** |  |  |  |
| MGI:1333774 | Mrpl42 | protein coding gene | ENSMUSG00000062981 | **x** | **x** |  |  |  |
| MGI:3041208 | 4732465J04Rik | lncRNA gene | ENSMUSG00000101517 | **x** | **x** |  |  |  |
| MGI:2442192 | Eea1 | protein coding gene | ENSMUSG00000036499 | **x** | **x** |  |  |  |
| MGI:94872 | Dcn | protein coding gene | ENSMUSG00000019929 | **x** | **x** | **x** |  |  |
| MGI:109347 | Lum | protein coding gene | ENSMUSG00000036446 | **x** | **x** |  |  |  |
| MGI:104653 | Atp2b1 | protein coding gene | ENSMUSG00000019943 | **x** | **x** | **x** |  |  |
| MGI:1914853 | Dusp6 | protein coding gene | ENSMUSG00000019960 | **x** | **x** | **x** | **x** |  |
| MGI:3036255 | Tmtc3 | protein coding gene | ENSMUSG00000036676 | **x** | **x** |  |  |  |
| MGI:1202907 | Csrp2 | protein coding gene | ENSMUSG00000020186 | **x** | **x** |  |  |  |
| MGI:2443807 | Osbpl8 | protein coding gene | ENSMUSG00000020189 | **x** | **x** |  |  |  |
| MGI:1919019 | Bbs10 | protein coding gene | ENSMUSG00000035759 | **x** | **x** |  |  |  |
| MGI:1855693 | Nap1l1 | protein coding gene | ENSMUSG00000058799 | **x** | **x** |  |  |  |
| MGI:1096880 | Phlda1 | protein coding gene | ENSMUSG00000020205 | **x** | **x** |  |  |  |
| MGI:96668 | Kcnc2 | protein coding gene | ENSMUSG00000035681 | **x** | **x** | **x** |  |  |
| MGI:97809 | Ptprb | protein coding gene | ENSMUSG00000020154 | **x** | **x** |  |  |  |
| MGI:105933 | Rab3ip | protein coding gene | ENSMUSG00000064181 | **x** | **x** |  |  |  |
| MGI:2143561 | D630029K05Rik | lncRNA gene | ENSMUSG00000031294 | **x** | **x** |  |  |  |
| MGI:2448063 | Lrrc10 | protein coding gene | ENSMUSG00000060187 | **x** | **x** | **x** | **x** | **x** |
| MGI:1100860 | Frs2 | protein coding gene | ENSMUSG00000020170 | **x** | **x** | **x** | **x** |  |
| MGI:96952 | Mdm2 | protein coding gene | ENSMUSG00000020184 | **x** | **x** | **x** | **x** |  |
| MGI:1330301 | Dyrk2 | protein coding gene | ENSMUSG00000028630 | **x** | **x** |  |  |  |
| MGI:2443538 | Msrb3 | protein coding gene | ENSMUSG00000051236 | **x** | **x** | **x** |  |  |
| MGI:1859216 | Avpr1a | protein coding gene | ENSMUSG00000020123 | **x** | **x** | **x** | **x** |  |
| MGI:1914532 | Washc3 | protein coding gene | ENSMUSG00000020056 | **x** |  |  |  |  |
| MGI:1918962 | Dram1 | protein coding gene | ENSMUSG00000020057 |  | **x** |  |  |  |
| MGI:3643902 | Gnptab | protein coding gene | ENSMUSG00000035311 |  | **x** |  |  |  |
| MGI:3801878 | Gm16235 | lncRNA gene | ENSMUSG00000087429 |  | **x** |  |  |  |
| MGI:109542 | Sycp3 | protein coding gene | ENSMUSG00000020059 |  | **x** |  |  |  |
| MGI:1336213 | Mybpc1 | protein coding gene | ENSMUSG00000020061 |  | **x** |  |  |  |
| MGI:1341168 | Spic | protein coding gene | ENSMUSG00000004359 |  | **x** |  |  |  |
| MGI:3643371 | Gm4925 | pseudogene | ENSMUSG00000069540 |  | **x** |  |  |  |
| MGI:99436 | Arl1 | protein coding gene | ENSMUSG00000060904 |  | **x** |  |  |  |
| MGI:1917933 | Utp20 | protein coding gene | ENSMUSG00000004356 |  | **x** |  |  |  |
| MGI:2384916 | Slc5a8 | protein coding gene | ENSMUSG00000020062 |  | **x** |  |  |  |
| MGI:2443344 | Ano4 | protein coding gene | ENSMUSG00000035189 |  | **x** |  |  |  |
| MGI:1352464 | Nr1h4 | protein coding gene | ENSMUSG00000047638 |  | **x** |  |  |  |
| MGI:3039629 | Slc17a8 | protein coding gene | ENSMUSG00000019935 |  | **x** | **x** |  |  |
| MGI:1289172 | Scyl2 | protein coding gene | ENSMUSG00000069539 |  | **x** |  |  |  |
| MGI:1914269 | Actr6 | protein coding gene | ENSMUSG00000019948 |  | **x** |  |  |  |
| MGI:2442888 | Bltp3b | protein coding gene | ENSMUSG00000019951 |  | **x** |  |  |  |
| MGI:1924781 | Anks1b | protein coding gene | ENSMUSG00000058589 |  | **x** |  |  |  |
| MGI:1914704 | Ikbip | protein coding gene | ENSMUSG00000019975 |  | **x** |  |  |  |
| MGI:5453896 | Gm24119 | snoRNA gene | ENSMUSG00000077167 |  | **x** |  |  |  |
| MGI:5434113 | Gm20757 | lncRNA gene | ENSMUSG00000098040 |  | **x** |  |  |  |
| MGI:1922208 | Cfap54 | protein coding gene | ENSMUSG00000020014 |  | **x** |  |  |  |
| MGI:97517 | Cdk17 | protein coding gene | ENSMUSG00000020015 |  | **x** |  |  |  |
| MGI:101762 | Elk3 | protein coding gene | ENSMUSG00000008398 |  | **x** |  |  |  |
| MGI:96010 | Hal | protein coding gene | ENSMUSG00000020017 |  | **x** |  |  |  |
| MGI:1919011 | Amdhd1 | protein coding gene | ENSMUSG00000015890 |  | **x** |  |  |  |
| MGI:2444738 | Ccdc38 | protein coding gene | ENSMUSG00000036168 |  | **x** |  |  |  |
| MGI:1917128 | Snrpf | protein coding gene | ENSMUSG00000020018 |  | **x** |  |  |  |
| MGI:3801763 | Gm15915 | lncRNA gene | ENSMUSG00000085723 |  | **x** |  |  |  |
| MGI:3781748 | Gm3571 | pseudogene | ENSMUSG00000090610 |  | **x** |  |  |  |
| MGI:1929701 | Metap2 | protein coding gene | ENSMUSG00000036112 |  | **x** |  |  |  |
| MGI:1261419 | Fgd6 | protein coding gene | ENSMUSG00000020021 |  | **x** |  |  |  |
| MGI:1352465 | Nr2c1 | protein coding gene | ENSMUSG00000005897 |  | **x** |  |  |  |
| MGI:1913664 | Ndufa12 | protein coding gene | ENSMUSG00000020022 |  | **x** | **x** |  |  |
| MGI:2442900 | Tmcc3 | protein coding gene | ENSMUSG00000020023 |  | **x** |  |  |  |
| MGI:3801736 | Tmcc3os | antisense lncRNA gene | ENSMUSG00000086269 |  | **x** |  |  |  |
| MGI:5530918 | Mir7211 | miRNA gene | ENSMUSG00000098544 |  | **x** |  |  |  |
| MGI:1914973 | Cep83os | bidirectional promoter lncRNA gene | ENSMUSG00000097164 |  | **x** |  |  |  |
| MGI:1924298 | Cep83 | protein coding gene | ENSMUSG00000020024 |  | **x** |  |  |  |
| MGI:1890127 | Plxnc1 | protein coding gene | ENSMUSG00000074785 |  | **x** |  |  |  |
| MGI:1916879 | 2310039L15Rik | lncRNA gene | ENSMUSG00000100550 |  | **x** |  |  |  |
| MGI:1201787 | Socs2 | protein coding gene | ENSMUSG00000020027 |  | **x** |  |  |  |
| MGI:1917773 | 5730420D15Rik | lncRNA gene | ENSMUSG00000097766 |  | **x** |  |  |  |
| MGI:1934835 | Ube2n | protein coding gene | ENSMUSG00000074781 |  | **x** |  |  |  |
| MGI:1918457 | Nudt4 | protein coding gene | ENSMUSG00000020029 |  | **x** |  |  |  |
| MGI:4834226 | Mir3058 | miRNA gene | ENSMUSG00000092867 |  | **x** |  |  |  |
| MGI:3646302 | Anapc15-ps | pseudogene | ENSMUSG00000074780 |  | **x** |  |  |  |
| MGI:88215 | Btg1 | protein coding gene | ENSMUSG00000036478 |  | **x** |  |  |  |
| MGI:107942 | Epyc | protein coding gene | ENSMUSG00000019936 |  | **x** |  |  |  |
| MGI:1918511 | Poc1b | protein coding gene | ENSMUSG00000019952 |  | **x** |  |  |  |
| MGI:894692 | Galnt4 | protein coding gene | ENSMUSG00000090035 |  | **x** |  |  |  |
| MGI:96974 | Kitl | protein coding gene | ENSMUSG00000019966 |  | **x** |  |  |  |
| MGI:2384917 | Cep290 | protein coding gene | ENSMUSG00000019971 |  | **x** | **x** | **x** |  |
| MGI:1921197 | Rlig1 | protein coding gene | ENSMUSG00000046567 |  | **x** | **x** |  |  |
| MGI:1328351 | Nts | protein coding gene | ENSMUSG00000019890 |  | **x** |  |  |  |
| MGI:2384307 | Rassf9 | protein coding gene | ENSMUSG00000044921 |  | **x** |  |  |  |
| MGI:2143484 | Slc6a15 | protein coding gene | ENSMUSG00000019894 |  | **x** |  |  |  |
| MGI:1914057 | Tmtc2 | protein coding gene | ENSMUSG00000036019 |  | **x** |  |  |  |
| MGI:3783108 | Gm15666 | pseudogene | ENSMUSG00000084399 |  | **x** |  |  |  |
| MGI:3783105 | Gm15663 | lncRNA gene | ENSMUSG00000085282 |  | **x** |  |  |  |
| MGI:3041259 | Mettl25 | protein coding gene | ENSMUSG00000036009 |  | **x** |  |  |  |
| MGI:1289302 | Ccdc59 | protein coding gene | ENSMUSG00000019897 |  | **x** |  |  |  |
| MGI:2443834 | Ppfia2 | protein coding gene | ENSMUSG00000053825 |  | **x** |  |  |  |
| MGI:2685720 | Acss3 | protein coding gene | ENSMUSG00000035948 |  | **x** |  |  |  |
| MGI:2135609 | Lin7a | protein coding gene | ENSMUSG00000019906 |  | **x** |  |  |  |
| MGI:97253 | Myf6 | protein coding gene | ENSMUSG00000035923 |  | **x** |  |  |  |
| MGI:1096349 | Ptprq | protein coding gene | ENSMUSG00000035916 |  | **x** |  |  |  |
| MGI:3647600 | Otogl | protein coding gene | ENSMUSG00000091455 |  | **x** |  |  |  |
| MGI:1309528 | Ppp1r12a | protein coding gene | ENSMUSG00000019907 |  | **x** |  |  |  |
| MGI:2149961 | Pawr | protein coding gene | ENSMUSG00000035873 |  | **x** |  |  |  |
| MGI:99667 | Syt1 | protein coding gene | ENSMUSG00000035864 |  | **x** |  |  |  |
| MGI:2183703 | Nav3 | protein coding gene | ENSMUSG00000020181 |  | **x** |  |  |  |
| MGI:1289147 | E2f7 | protein coding gene | ENSMUSG00000020185 |  | **x** |  |  |  |
| MGI:2445110 | Zdhhc17 | protein coding gene | ENSMUSG00000035798 |  | **x** |  |  |  |
| MGI:5454894 | Gm25117 | snoRNA gene | ENSMUSG00000087819 |  | **x** |  |  |  |
| MGI:1918571 | 4933440J02Rik | lncRNA gene | ENSMUSG00000097082 |  | **x** |  |  |  |
| MGI:1289274 | Krr1 | protein coding gene | ENSMUSG00000063334 |  | **x** |  |  |  |
| MGI:1920940 | Glipr1 | protein coding gene | ENSMUSG00000056888 |  | **x** |  |  |  |
| MGI:3801764 | Gm15914 | pseudogene | ENSMUSG00000089667 |  | **x** |  |  |  |
| MGI:1914971 | Atxn7l3b | protein coding gene | ENSMUSG00000074748 |  | **x** |  |  |  |
| MGI:2384311 | Trhde | protein coding gene | ENSMUSG00000050663 |  | **x** |  |  |  |
| MGI:1913937 | Tbc1d15 | protein coding gene | ENSMUSG00000020130 |  | **x** |  |  |  |
| MGI:894308 | Rab21 | protein coding gene | ENSMUSG00000020132 |  | **x** |  |  |  |
| MGI:1914476 | Tmem19 | protein coding gene | ENSMUSG00000069520 |  | **x** |  |  |  |
| MGI:1914066 | Thap2 | protein coding gene | ENSMUSG00000020137 |  | **x** |  |  |  |
| MGI:2446143 | Zfc3h1 | protein coding gene | ENSMUSG00000034163 |  | **x** |  |  |  |
| MGI:1341817 | Lgr5 | protein coding gene | ENSMUSG00000020140 |  | **x** |  |  |  |
| MGI:2384918 | Tspan8 | protein coding gene | ENSMUSG00000034127 |  | **x** |  |  |  |
| MGI:109559 | Ptprr | protein coding gene | ENSMUSG00000020151 |  | **x** |  |  |  |
| MGI:1919318 | Cnot2 | protein coding gene | ENSMUSG00000020166 |  | **x** |  |  |  |
| MGI:3026931 | 5330438D12Rik | lncRNA gene | ENSMUSG00000052291 |  | **x** |  |  |  |
| MGI:2685085 | Myrfl | protein coding gene | ENSMUSG00000034057 |  | **x** |  |  |  |
| MGI:3580298 | Best3 | protein coding gene | ENSMUSG00000020169 |  | **x** |  |  |  |
| MGI:107186 | Cct2 | protein coding gene | ENSMUSG00000034024 |  | **x** |  |  |  |
| MGI:1927224 | Yeats4 | protein coding gene | ENSMUSG00000020171 |  | **x** |  |  |  |
| MGI:96897 | Lyz2 | protein coding gene | ENSMUSG00000069516 |  | **x** |  |  |  |
| MGI:96902 | Lyz1 | protein coding gene | ENSMUSG00000069515 |  | **x** |  |  |  |
| MGI:1913948 | Cpsf6 | protein coding gene | ENSMUSG00000055531 |  | **x** |  |  |  |
| MGI:1917824 | Cpm | protein coding gene | ENSMUSG00000020183 |  | **x** |  |  |  |
| MGI:2448489 | Slc35e3 | protein coding gene | ENSMUSG00000060181 |  | **x** |  |  |  |
| MGI:2143854 | Nup107 | protein coding gene | ENSMUSG00000052798 |  | **x** |  |  |  |
| MGI:894315 | Rap1b | protein coding gene | ENSMUSG00000052681 |  | **x** |  |  |  |
| MGI:96951 | Mdm1 | protein coding gene | ENSMUSG00000020212 |  | **x** |  |  |  |
| MGI:1921665 | 4932442E05Rik | protein coding gene | ENSMUSG00000104443 |  | **x** |  |  |  |
| MGI:1261820 | Cand1 | protein coding gene | ENSMUSG00000020114 |  | **x** |  |  |  |
| MGI:1921303 | Grip1 | protein coding gene | ENSMUSG00000034813 |  | **x** |  |  |  |
| MGI:1922226 | Grip1os1 | antisense lncRNA gene | ENSMUSG00000085418 |  | **x** |  |  |  |
| MGI:2152895 | Helb | protein coding gene | ENSMUSG00000020228 |  | **x** |  |  |  |
| MGI:1921164 | Irak3 | protein coding gene | ENSMUSG00000020227 |  | **x** |  |  |  |
| MGI:1915462 | Tmbim4 | protein coding gene | ENSMUSG00000020225 |  | **x** |  |  |  |
| MGI:1913475 | Llph | protein coding gene | ENSMUSG00000020224 |  | **x** |  |  |  |
| MGI:101761 | Hmga2 | protein coding gene | ENSMUSG00000056758 |  | **x** |  |  |  |
| MGI:2444388 | 9230105E05Rik | lncRNA gene | ENSMUSG00000090191 |  | **x** |  |  |  |
| MGI:1918124 | 4921513I03Rik | lncRNA gene | ENSMUSG00000044544 |  | **x** |  |  |  |
| MGI:3802142 | Gm15961 | lncRNA gene | ENSMUSG00000086444 |  | **x** |  |  |  |
| MGI:3802158 | Gm15910 | lncRNA gene | ENSMUSG00000087480 |  | **x** |  |  |  |
| MGI:3782657 | Gm4473 | lncRNA gene | ENSMUSG00000086522 |  | **x** |  |  |  |
| MGI:3580376 | Lemd3 | protein coding gene | ENSMUSG00000048661 |  | **x** |  |  |  |
| MGI:3801807 | Gm16166 | pseudogene | ENSMUSG00000082936 |  | **x** |  |  |  |
| MGI:1344332 | Wif1 | protein coding gene | ENSMUSG00000020218 |  | **x** | **x** |  |  |
| MGI:1921944 | Tbc1d30 | protein coding gene | ENSMUSG00000052302 |  | **x** |  |  |  |
| MGI:1922862 | Gns | protein coding gene | ENSMUSG00000034707 |  | **x** |  |  |  |
| MGI:2179722 | Rassf3 | protein coding gene | ENSMUSG00000025795 |  | **x** |  |  |  |
| MGI:1929658 | Tbk1 | protein coding gene | ENSMUSG00000020115 |  | **x** |  |  |  |
| MGI:1920442 | Xpot | protein coding gene | ENSMUSG00000034667 |  | **x** |  |  |  |
| MGI:2670984 | Kics2 | protein coding gene | ENSMUSG00000053684 |  | **x** |  |  |  |
| MGI:2152936 | Srgap1 | protein coding gene | ENSMUSG00000020121 |  | **x** |  |  |  |
| MGI:2384919 | Rxylt1 | protein coding gene | ENSMUSG00000034620 |  | **x** |  |  |  |
| MGI:3644454 | Gm9081 | pseudogene | ENSMUSG00000098138 |  | **x** |  |  |  |
| MGI:2442087 | Ppm1h | protein coding gene | ENSMUSG00000034613 |  | **x** |  |  |  |
| MGI:1914324 | Mon2 | protein coding gene | ENSMUSG00000034602 |  | **x** |  |  |  |
| MGI:101857 | Usp15 | protein coding gene | ENSMUSG00000020124 |  | **x** |  |  |  |
| MGI:2143691 | Tafa2 | protein coding gene | ENSMUSG00000044071 |  | **x** |  |  |  |
| MGI:1330284 | Slc16a7 | protein coding gene | ENSMUSG00000020102 |  | **x** |  |  |  |
| MGI:1913855 | 1700017N19Rik | protein coding gene | ENSMUSG00000056912 |  | **x** |  |  |  |
| MGI:1913272 | Kcnmb4 | protein coding gene | ENSMUSG00000054934 |  | **x** |  |  |  |
| MGI:6097756 | Gm48308 | unclassified gene | ENSMUSG00000112932 |  | **x** |  |  |  |
| MGI:5010165 | Gm17980 | pseudogene | ENSMUSG00000112259 |  | **x** |  |  |  |
| MGI:6097458 | Gm48106 | pseudogene | ENSMUSG00000112428 |  | **x** |  |  |  |
| MGI:5825829 | Gm46192 | pseudogene | ENSMUSG00000111919 |  | **x** |  |  |  |
| MGI:3645288 | Gm5780 | pseudogene | ENSMUSG00000112140 |  | **x** |  |  |  |
| MGI:1099806 | Rmst | lncRNA gene | ENSMUSG00000112117 |  | **x** |  |  |  |
| MGI:3648240 | Gm8512 | pseudogene | ENSMUSG00000111104 |  | **x** |  |  |  |
| MGI:3646193 | Gm4800 | pseudogene | ENSMUSG00000111759 |  | **x** |  |  |  |
| MGI:5454018 | Gm24241 | snoRNA gene | ENSMUSG00000088412 |  | **x** |  |  |  |
| MGI:6215303 | Gm49783 | unclassified gene | ENSMUSG00000111740 |  | **x** |  |  |  |
| MGI:3836967 | Mir1931 | miRNA gene | ENSMUSG00000089214 |  | **x** |  |  |  |
| MGI:5591847 | Gm32688 | lncRNA gene | ENSMUSG00000111531 |  | **x** |  |  |  |
| MGI:5009823 | Gm17745 | lncRNA gene | ENSMUSG00000110647 |  | **x** |  |  |  |
| MGI:1921704 | 4933408J17Rik | lncRNA gene | ENSMUSG00000097407 |  | **x** |  |  |  |
| MGI:3647055 | Gm8580 | pseudogene | ENSMUSG00000090159 |  | **x** |  |  |  |
| MGI:3802140 | Gm15963 | lncRNA gene | ENSMUSG00000086891 |  | **x** |  |  |  |
| MGI:3045318 | Usp44 | protein coding gene | ENSMUSG00000020020 |  | **x** |  |  |  |
| MGI:6215053 | Gm49628 | unclassified gene | ENSMUSG00000110869 |  | **x** |  |  |  |
| MGI:3801971 | Gm16155 | lncRNA gene | ENSMUSG00000085409 |  | **x** |  |  |  |
| MGI:6118596 | Gm49167 | unclassified gene | ENSMUSG00000112203 |  | **x** |  |  |  |
| MGI:6098317 | Gm48689 | lncRNA gene | ENSMUSG00000112669 |  | **x** |  |  |  |
| MGI:6098613 | Gm48868 | unclassified gene | ENSMUSG00000112677 |  | **x** |  |  |  |
| MGI:5588843 | Gm29684 | lncRNA gene | ENSMUSG00000112481 |  | **x** |  |  |  |
| MGI:6098634 | Gm48880 | unclassified gene | ENSMUSG00000112744 |  | **x** |  |  |  |
| MGI:6098637 | Gm48882 | lncRNA gene | ENSMUSG00000112593 |  | **x** |  |  |  |
| MGI:6096651 | Gm47599 | lncRNA gene | ENSMUSG00000112261 |  | **x** |  |  |  |
| MGI:6096827 | Gm47708 | lncRNA gene | ENSMUSG00000112188 |  | **x** |  |  |  |
| MGI:5592702 | Gm33543 | protein coding gene | ENSMUSG00000110353 |  | **x** |  |  |  |
| MGI:6097926 | Gm48427 | lncRNA gene | ENSMUSG00000112234 |  | **x** |  |  |  |
| MGI:6098057 | Gm48521 | lncRNA gene | ENSMUSG00000111912 |  | **x** |  |  |  |
| MGI:3648168 | Gm8613 | pseudogene | ENSMUSG00000112892 |  | **x** |  |  |  |
| MGI:4950405 | Mir3966 | miRNA gene | ENSMUSG00000105557 |  | **x** |  |  |  |
| MGI:5593456 | Gm34297 | lncRNA gene | ENSMUSG00000112874 |  | **x** |  |  |  |
| MGI:6098457 | Gm48768 | lncRNA gene | ENSMUSG00000112346 |  | **x** |  |  |  |
| MGI:3801746 | Gm16239 | lncRNA gene | ENSMUSG00000089994 |  | **x** |  |  |  |
| MGI:5593733 | Gm34574 | lncRNA gene | ENSMUSG00000112805 |  | **x** |  |  |  |
| MGI:5593936 | Gm34777 | lncRNA gene | ENSMUSG00000111923 |  | **x** |  |  |  |
| MGI:5594080 | Gm34921 | lncRNA gene | ENSMUSG00000112384 |  | **x** |  |  |  |
| MGI:6097430 | Gm48089 | lncRNA gene | ENSMUSG00000112544 |  | **x** |  |  |  |
| MGI:2445145 | B530045E10Rik | lncRNA gene | ENSMUSG00000044633 |  | **x** |  |  |  |
| MGI:95633 | Gad1-ps | pseudogene | ENSMUSG00000090665 |  | **x** |  |  |  |
| MGI:5594194 | Gm35035 | lncRNA gene | ENSMUSG00000112082 |  | **x** |  |  |  |
| MGI:1919082 | Csl | protein coding gene | ENSMUSG00000046934 |  | **x** |  |  |  |
| MGI:5594365 | Gm35206 | lncRNA gene | ENSMUSG00000112304 |  | **x** |  |  |  |
| MGI:5594692 | Gm35533 | lncRNA gene | ENSMUSG00000112831 |  | **x** |  |  |  |
| MGI:6096768 | Gm47673 | unclassified gene | ENSMUSG00000112580 |  | **x** |  |  |  |
| MGI:6097228 | Gm47956 | lncRNA gene | ENSMUSG00000112000 |  | **x** |  |  |  |
| MGI:6097597 | Gm48206 | unclassified gene | ENSMUSG00000112193 |  | **x** |  |  |  |
| MGI:6097591 | Gm48203 | unclassified gene | ENSMUSG00000112512 |  | **x** |  |  |  |
| MGI:6097599 | Gm48207 | unclassified gene | ENSMUSG00000112571 |  | **x** |  |  |  |
| MGI:6097740 | Gm48298 | unclassified gene | ENSMUSG00000112820 |  | **x** |  |  |  |
| MGI:3643034 | Rps15a-ps1 | pseudogene | ENSMUSG00000056877 |  | **x** |  |  |  |
| MGI:1923083 | 4930532I03Rik | lncRNA gene | ENSMUSG00000112201 |  | **x** |  |  |  |
| MGI:5595442 | Gm36283 | lncRNA gene | ENSMUSG00000112532 |  | **x** |  |  |  |
| MGI:6096432 | Gm47466 | pseudogene | ENSMUSG00000112065 |  | **x** |  |  |  |
| MGI:1925931 | 9230102K24Rik | lncRNA gene | ENSMUSG00000112398 |  | **x** |  |  |  |
| MGI:6098588 | Gm48851 | pseudogene | ENSMUSG00000112165 |  | **x** |  |  |  |
| MGI:3647789 | Rpl6l | pseudogene | ENSMUSG00000091086 |  | **x** |  |  |  |
| MGI:5010034 | Gm17849 | pseudogene | ENSMUSG00000112642 |  | **x** |  |  |  |
| MGI:3644450 | Gm5176 | pseudogene | ENSMUSG00000111997 |  | **x** |  |  |  |
| MGI:5589421 | Gm30262 | lncRNA gene | ENSMUSG00000112249 |  | **x** |  |  |  |
| MGI:5589783 | Gm30624 | lncRNA gene | ENSMUSG00000112430 |  | **x** |  |  |  |
| MGI:5011190 | Gm19005 | pseudogene | ENSMUSG00000112802 |  | **x** |  |  |  |
| MGI:5623650 | Gm40765 | lncRNA gene | ENSMUSG00000112087 |  | **x** |  |  |  |
| MGI:3783167 | Gm15723 | lncRNA gene | ENSMUSG00000084865 |  | **x** |  |  |  |
| MGI:2651811 | Tph2 | protein coding gene | ENSMUSG00000006764 |  | **x** | **x** |  |  |
| MGI:3645705 | Gm8942 | pseudogene | ENSMUSG00000111964 |  | **x** |  |  |  |
| MGI:3642645 | Gm10752 | unclassified gene | ENSMUSG00000112301 |  | **x** |  |  |  |
| MGI:2443640 | A130012E19Rik | lncRNA gene | ENSMUSG00000112822 |  | **x** |  |  |  |
| MGI:3642929 | Gm8960 | pseudogene | ENSMUSG00000112099 |  | **x** |  |  |  |
| MGI:6096692 | Gm47625 | unclassified gene | ENSMUSG00000112461 |  | **x** |  |  |  |
| MGI:6121539 | Gm49344 | unclassified gene | ENSMUSG00000112881 |  | **x** |  |  |  |
| MGI:5825840 | Gm46203 | pseudogene | ENSMUSG00000111937 |  | **x** |  |  |  |
| MGI:1918336 | 4933412E12Rik | lncRNA gene | ENSMUSG00000112627 |  | **x** |  |  |  |
| MGI:3646721 | Gm5781 | pseudogene | ENSMUSG00000111966 |  | **x** |  |  |  |
| MGI:3641810 | Gm10747 | lncRNA gene | ENSMUSG00000112242 |  | **x** |  |  |  |
| MGI:5591300 | Gm32141 | lncRNA gene | ENSMUSG00000112344 |  | **x** |  |  |  |
| MGI:1918317 | 4933411E08Rik | lncRNA gene | ENSMUSG00000112500 |  | **x** |  |  |  |
| MGI:5611080 | Gm37852 | unclassified gene | ENSMUSG00000102896 |  | **x** |  |  |  |
| MGI:6097232 | Gm47958 | lncRNA gene | ENSMUSG00000112684 |  | **x** |  |  |  |
| MGI:3646677 | Cdc5lrt3-ps | pseudogene | ENSMUSG00000112614 |  | **x** |  |  |  |
| MGI:5591961 | Cdc5lrt4 | protein coding gene | ENSMUSG00000112252 |  | **x** |  |  |  |
| MGI:3782240 | Gm4065 | lncRNA gene | ENSMUSG00000112198 |  | **x** |  |  |  |
| MGI:5621288 | Gm38403 | lncRNA gene | ENSMUSG00000112854 |  | **x** |  |  |  |
| MGI:5592836 | Gm33677 | lncRNA gene | ENSMUSG00000112778 |  | **x** |  |  |  |
| MGI:6096454 | Gm47480 | lncRNA gene | ENSMUSG00000112609 |  | **x** |  |  |  |
| MGI:6095713 | Gm47024 | unclassified gene | ENSMUSG00000112287 |  | **x** |  |  |  |
| MGI:6096236 | Gm47342 | lncRNA gene | ENSMUSG00000112243 |  | **x** |  |  |  |
| MGI:6095688 | Gm47009 | lncRNA gene | ENSMUSG00000112894 |  | **x** |  |  |  |
| MGI:6097469 | Gm48115 | unclassified gene | ENSMUSG00000112322 |  | **x** |  |  |  |
| MGI:2447818 | 1700006J14Rik | lncRNA gene | ENSMUSG00000034764 |  | **x** |  |  |  |
| MGI:3642299 | Gm10741 | lncRNA gene | ENSMUSG00000112431 |  | **x** |  |  |  |
| MGI:6098163 | Gm48591 | lncRNA gene | ENSMUSG00000112009 |  | **x** |  |  |  |
| MGI:5623672 | Gm40787 | lncRNA gene | ENSMUSG00000112539 |  | **x** |  |  |  |
| MGI:6098514 | Gm48804 | unclassified gene | ENSMUSG00000112596 |  | **x** |  |  |  |
| MGI:6098630 | Gm48878 | unclassified gene | ENSMUSG00000112794 |  | **x** |  |  |  |
| MGI:2441782 | Sros1 | lncRNA gene | ENSMUSG00000112095 |  | **x** |  |  |  |
| MGI:2444187 | A430028G04Rik | lncRNA gene | ENSMUSG00000112417 |  | **x** |  |  |  |
| MGI:5595367 | Gm36208 | lncRNA gene | ENSMUSG00000112220 |  | **x** |  |  |  |
| MGI:3028060 | D630033A02Rik | lncRNA gene | ENSMUSG00000112237 |  | **x** |  |  |  |
| MGI:3782305 | Gm4129 | lncRNA gene | ENSMUSG00000112294 |  | **x** |  |  |  |
| MGI:6097078 | Gm47862 | unclassified gene | ENSMUSG00000111943 |  | **x** |  |  |  |

**Supplementary Table S9. Cardiac GSH phenotypes exhibit mild to moderate heritability.**

Heritability (H^2^) was estimated using R/qtl2 and kinship was accounted for using a linear mixed model.

| Phenotype | Heritability |
| --- | --- |
| Cardiac GSH | 0.285 |
| Cardiac GSSG | 0.205 |
| Cardiac Total GSH | 0.094 |
| Cardiac GSH/GSSG | 0.342 |
| Cardiac redox potential | 0.464 |

**Supplementary Table S10. Chr 14 GSH candidate genes and their relevant annotations (Chr14:48.909-55.303±1 Mbp; GRCm38/mm10)^a^.**

^a^Resource abbreviations: **EEA,** EBI Expression Atlas; **DO,** Disease Ontology; **GO,** Gene Ontology; **GXD,** Gene eXpression Database; **MGI,** Mouse Genome Informatics; **MP,** Mammalian Phenotype.

|  |  |  |  | **Expression** | | **Functional** | **Phenotypic (cardiac-related)** | |
| --- | --- | --- | --- | --- | --- | --- | --- | --- |
| **MGI Gene/Marker ID** | **Symbol** | **Feature Type** | **Ensembl ID** | **GXD** | **EEA** | **GO** | **MP** | **DO** |
| MGI:1914669 | Armh4 | protein coding gene | ENSMUSG00000036242 | **x** |  |  |  |  |
| MGI:2685134 | Ccnb1ip1 | protein coding gene | ENSMUSG00000071470 | **x** |  |  |  |  |
| MGI:1341112 | Parp2 | protein coding gene | ENSMUSG00000036023 | **x** |  | **x** |  |  |
| MGI:97919 | Rnase1 | protein coding gene | ENSMUSG00000035896 | **x** |  |  |  |  |
| MGI:1352498 | Ndrg2 | protein coding gene | ENSMUSG00000004558 | **x** |  | **x** |  |  |
| MGI:1917140 | Zfp219 | protein coding gene | ENSMUSG00000049295 | **x** |  |  |  |  |
| MGI:107795 | Hnrnpc | protein coding gene | ENSMUSG00000060373 | **x** |  |  |  |  |
| MGI:1915022 | Chd8 | protein coding gene | ENSMUSG00000053754 | **x** |  |  |  |  |
| MGI:1915389 | Tox4 | protein coding gene | ENSMUSG00000016831 | **x** |  |  |  |  |
| MGI:4439838 | Trac | gene segment | ENSMUSG00000076928 | **x** |  |  |  |  |
| MGI:101912 | Dad1 | protein coding gene | ENSMUSG00000022174 | **x** |  |  |  |  |
| MGI:1337120 | Slc7a7 | protein coding gene | ENSMUSG00000000958 | **x** |  | **x** | **x** |  |
| MGI:1916086 | Mrpl52 | protein coding gene | ENSMUSG00000010406 | **x** |  |  |  |  |
| MGI:101900 | Mmp14 | protein coding gene | ENSMUSG00000000957 | **x** |  |  |  |  |
| MGI:1929480 | Lrp10 | protein coding gene | ENSMUSG00000022175 | **x** |  |  |  |  |
| MGI:1355323 | Slc7a8 | protein coding gene | ENSMUSG00000022180 | **x** |  | **x** |  |  |
| MGI:105311 | Efs | protein coding gene | ENSMUSG00000022203 | **x** |  |  |  |  |
| MGI:97255 | Myh6 | protein coding gene | ENSMUSG00000040752 | **x** |  | **x** | **x** | **x** |
| MGI:2676883 | Mir208a | miRNA gene | ENSMUSG00000065432 | **x** |  | **x** | **x** |  |
| MGI:3642848 | Mhrt | lncRNA gene | ENSMUSG00000097652 | **x** |  |  |  |  |
| MGI:2155600 | Myh7 | protein coding gene | ENSMUSG00000053093 | **x** |  | **x** | **x** |  |
| MGI:3718461 | Mir208b | miRNA gene | ENSMUSG00000077928 | **x** |  | **x** |  |  |
| MGI:2686934 | Zfhx2 | protein coding gene | ENSMUSG00000040721 | **x** |  |  |  |  |
| MGI:1934682 | Emc9 | protein coding gene | ENSMUSG00000022217 | **x** |  |  |  |  |
| MGI:1096365 | Psme2 | protein coding gene | ENSMUSG00000079197 | **x** |  |  |  |  |
| MGI:1934704 | Rnf31 | protein coding gene | ENSMUSG00000047098 | **x** |  |  |  |  |
| MGI:107587 | Irf9 | protein coding gene | ENSMUSG00000002325 | **x** |  |  |  |  |
| MGI:1915131 | Mdp1 | protein coding gene | ENSMUSG00000002329 | **x** |  |  |  |  |
| MGI:97301 | Nedd8 | protein coding gene | ENSMUSG00000010376 | **x** |  |  |  |  |
| MGI:107246 | Tinf2 | protein coding gene | ENSMUSG00000007589 | **x** |  |  |  |  |
| MGI:99674 | Adcy4 | protein coding gene | ENSMUSG00000022220 | **x** |  |  |  |  |
| MGI:2154952 | Ripk3 | protein coding gene | ENSMUSG00000022221 | **x** |  | **x** |  |  |
| MGI:1920431 | Nfatc4 | protein coding gene | ENSMUSG00000023411 | **x** |  | **x** | **x** |  |
| MGI:3583292 | Otx2os1 | lincRNA | ENSMUSG00000098682 |  | **x** |  |  |  |
| MGI:1922538 | Slc35f4 | protein coding | ENSMUSG00000021852 |  | **x** |  |  |  |
| MGI:3650121 | Trav8d-1 | TR V gene | ENSMUSG00000076770 |  | **x** |  |  |  |
| MGI:3650631 | Trav9d-1 | TR V gene | ENSMUSG00000096478 |  | **x** |  |  |  |
| MGI:3649416 | Trav6d-5 | TR V gene | ENSMUSG00000095426 |  | **x** |  |  |  |
| MGI:3782037 | Trav4d-3 | TR V gene | ENSMUSG00000093939 |  | **x** |  |  |  |
| MGI:3647073 | Rps19-ps2 | Processed pseudogene | ENSMUSG00000094974 |  | **x** |  |  |  |
| MGI:2686335 | Trav6d-7 | TR V gene | ENSMUSG00000095756 |  | **x** |  |  |  |
| MGI:5293419 | Trav13d-3 | TR V gene | ENSMUSG00000095643 |  | **x** |  |  |  |
| MGI:3704436 | Trav16d-dv11 | TR V gene | ENSMUSG00000076796 |  | **x** |  |  |  |
| MGI:3647760 | Trav8d-2 | TR V gene | ENSMUSG00000076795 |  | **x** |  |  |  |
| MGI:4440515 | Trav13d-4 | TR V gene | ENSMUSG00000096329 |  | **x** |  |  |  |
| MGI:3822547 | Trav14d-3-dv8 | TR V gene | ENSMUSG00000094619 |  | **x** |  |  |  |
| MGI:3809200 | Trav9n-1 | TR V pseudogene | ENSMUSG00000092251 |  | **x** |  |  |  |
| MGI:3704437 | Trav6n-5 | TR V gene | ENSMUSG00000076800 |  | **x** |  |  |  |

**Supplementary Table S11. Top variants within +/- 1 Mbp of the 95% Bayesian credible interval for the Chr 14 GSH QTL.**

| snp | chr | pos | lod | ensembl_gene_id | consequence | description | chromosome_name | start_position | end_position | strand |
| --- | --- | --- | --- | --- | --- | --- | --- | --- | --- | --- |
| rs51357179 | 14 | 48.02473 | 1.807696 |  | intergenic_variant | NA | NA | NA | NA | NA |
| rs31523260 | 14 | 48.55554 | 1.835033 |  | intergenic_variant | NA | NA | NA | NA | NA |
| rs50150098 | 14 | 48.77541 | 1.832831 | ENSMUSG00000098682 | ENSMUSG00000098682:intron_variant,ENSMUSG00000098682:non_coding_transcript_variant | orthodenticle homeobox 2 opposite strand 1 [Source:MGI Symbol;Acc:MGI:3583292] | 14 | 48906165 | 49109111 | 1 |
| SV_14_48779257_48779275 | 14 | 48.77927 | 1.832831 | NA | NA | NA | NA | NA | NA | NA |
| SV_14_48828391_48828548 | 14 | 48.82847 | 1.79911 | NA | NA | NA | NA | NA | NA | NA |
| 14:48881954_G/A | 14 | 48.88195 | 1.670297 |  | intergenic_variant | NA | NA | NA | NA | NA |
| SV_14_48956723_48956986 | 14 | 48.95685 | 1.737438 | NA | NA | NA | NA | NA | NA | NA |
| rs233172300 | 14 | 49.29198 | 1.676231 |  | intergenic_variant | NA | NA | NA | NA | NA |
| rs587249600 | 14 | 49.38534 | 3.154105 | ENSMUSG00000021852 | ENSMUSG00000021852:intron_variant,ENSMUSG00000021852:non_coding_transcript_variant | solute carrier family 35, member F4 [Source:MGI Symbol;Acc:MGI:1922538] | 14 | 49535976 | 49763503 | -1 |
| SV_14_49396550_49396687 | 14 | 49.39662 | 2.277656 | NA | NA | NA | NA | NA | NA | NA |
| SV_14_49878403_49885148 | 14 | 49.88178 | 1.815075 | NA | NA | NA | NA | NA | NA | NA |
| SV_14_50306834_50307152 | 14 | 50.30699 | 2.270554 | NA | NA | NA | NA | NA | NA | NA |
| SV_14_52001626_52001848 | 14 | 52.00174 | 1.866519 | NA | NA | NA | NA | NA | NA | NA |
| 14:52783834_C/T | 14 | 52.78383 | 1.824103 | ENSMUSG00000076770 | ENSMUSG00000076770:downstream_gene_variant | T cell receptor alpha variable 8D-1 [Source:MGI Symbol;Acc:MGI:3650121] | 14 | 53015904 | 53016452 | 1 |
| 14:52784086_T/A | 14 | 52.78409 | 1.824103 |  | intergenic_variant | NA | NA | NA | NA | NA |
| 14:52784512_C/T | 14 | 52.78451 | 1.824103 |  | intergenic_variant | NA | NA | NA | NA | NA |
| 14:52784536_T/A | 14 | 52.78454 | 1.824103 |  | intergenic_variant | NA | NA | NA | NA | NA |
| 14:52785442_C/T | 14 | 52.78544 | 1.824103 |  | intergenic_variant | NA | NA | NA | NA | NA |
| 14:52785448_T/C | 14 | 52.78545 | 1.824103 |  | intergenic_variant | NA | NA | NA | NA | NA |
| 14:52785482_T/A | 14 | 52.78548 | 1.824103 |  | intergenic_variant | NA | NA | NA | NA | NA |
| 14:52785490_A/C | 14 | 52.78549 | 1.824103 |  | intergenic_variant | NA | NA | NA | NA | NA |
| 14:52785499_A/T | 14 | 52.7855 | 1.824103 |  | intergenic_variant | NA | NA | NA | NA | NA |
| 14:52785638_A/G | 14 | 52.78564 | 1.824103 |  | intergenic_variant | NA | NA | NA | NA | NA |
| 14:52785691_A/G | 14 | 52.78569 | 1.824103 |  | intergenic_variant | NA | NA | NA | NA | NA |
| 14:52785714_T/C | 14 | 52.78571 | 1.824103 |  | intergenic_variant | NA | NA | NA | NA | NA |
| 14:52785760_C/T | 14 | 52.78576 | 1.824103 |  | intergenic_variant | NA | NA | NA | NA | NA |
| 14:52785892_A/T | 14 | 52.78589 | 1.824103 |  | intergenic_variant | NA | NA | NA | NA | NA |
| 14:52785910_C/T | 14 | 52.78591 | 1.824103 |  | intergenic_variant | NA | NA | NA | NA | NA |
| 14:52785977_G/A | 14 | 52.78598 | 1.824103 |  | intergenic_variant | NA | NA | NA | NA | NA |
| 14:52786067_G/C | 14 | 52.78607 | 1.824103 |  | intergenic_variant | NA | NA | NA | NA | NA |
| 14:52786112_G/C | 14 | 52.78611 | 1.824103 |  | intergenic_variant | NA | NA | NA | NA | NA |
| 14:52786178_A/G | 14 | 52.78618 | 1.824103 |  | intergenic_variant | NA | NA | NA | NA | NA |
| 14:52786288_G/A | 14 | 52.78629 | 1.824103 |  | intergenic_variant | NA | NA | NA | NA | NA |
| 14:52786302_A/G | 14 | 52.7863 | 1.824103 |  | intergenic_variant | NA | NA | NA | NA | NA |
| 14:52786513_G/A | 14 | 52.78651 | 1.824103 |  | intergenic_variant | NA | NA | NA | NA | NA |
| 14:52786522_T/C | 14 | 52.78652 | 1.824103 |  | intergenic_variant | NA | NA | NA | NA | NA |
| 14:52786724_C/T | 14 | 52.78672 | 1.824103 |  | intergenic_variant | NA | NA | NA | NA | NA |
| 14:52786725_A/G | 14 | 52.78673 | 1.824103 |  | intergenic_variant | NA | NA | NA | NA | NA |
| 14:52786739_G/C | 14 | 52.78674 | 1.824103 |  | intergenic_variant | NA | NA | NA | NA | NA |
| 14:52786785_A/G | 14 | 52.78679 | 1.824103 |  | intergenic_variant | NA | NA | NA | NA | NA |
| 14:52786820_G/A | 14 | 52.78682 | 1.824103 |  | intergenic_variant | NA | NA | NA | NA | NA |
| 14:52786876_C/G | 14 | 52.78688 | 1.824103 |  | intergenic_variant | NA | NA | NA | NA | NA |
| 14:52786966_A/G | 14 | 52.78697 | 1.824103 |  | intergenic_variant | NA | NA | NA | NA | NA |
| 14:52787157_T/C | 14 | 52.78716 | 1.824103 |  | intergenic_variant | NA | NA | NA | NA | NA |
| 14:52787176_C/T | 14 | 52.78718 | 1.824103 |  | intergenic_variant | NA | NA | NA | NA | NA |
| 14:52787216_A/G | 14 | 52.78722 | 1.824103 | ENSMUSG00000096478 | ENSMUSG00000096478:upstream_gene_variant | T cell receptor alpha variable 9D-1 [Source:MGI Symbol;Acc:MGI:3650631] | 14 | 53029773 | 53030234 | 1 |
| 14:52787237_T/G | 14 | 52.78724 | 1.824103 | ENSMUSG00000096478 | ENSMUSG00000096478:upstream_gene_variant | T cell receptor alpha variable 9D-1 [Source:MGI Symbol;Acc:MGI:3650631] | 14 | 53029773 | 53030234 | 1 |
| 14:52787239_G/T | 14 | 52.78724 | 1.824103 | ENSMUSG00000096478 | ENSMUSG00000096478:upstream_gene_variant | T cell receptor alpha variable 9D-1 [Source:MGI Symbol;Acc:MGI:3650631] | 14 | 53029773 | 53030234 | 1 |
| 14:52787266_A/G | 14 | 52.78727 | 1.824103 | ENSMUSG00000096478 | ENSMUSG00000096478:upstream_gene_variant | T cell receptor alpha variable 9D-1 [Source:MGI Symbol;Acc:MGI:3650631] | 14 | 53029773 | 53030234 | 1 |
| 14:52787273_G/A | 14 | 52.78727 | 1.824103 | ENSMUSG00000096478 | ENSMUSG00000096478:upstream_gene_variant | T cell receptor alpha variable 9D-1 [Source:MGI Symbol;Acc:MGI:3650631] | 14 | 53029773 | 53030234 | 1 |
| 14:52787286_G/T | 14 | 52.78729 | 1.824103 | ENSMUSG00000096478 | ENSMUSG00000096478:upstream_gene_variant | T cell receptor alpha variable 9D-1 [Source:MGI Symbol;Acc:MGI:3650631] | 14 | 53029773 | 53030234 | 1 |
| 14:52788101_A/C | 14 | 52.7881 | 1.824103 | ENSMUSG00000096478 | ENSMUSG00000096478:upstream_gene_variant | T cell receptor alpha variable 9D-1 [Source:MGI Symbol;Acc:MGI:3650631] | 14 | 53029773 | 53030234 | 1 |
| 14:52788109_C/G | 14 | 52.78811 | 1.824103 | ENSMUSG00000096478 | ENSMUSG00000096478:upstream_gene_variant | T cell receptor alpha variable 9D-1 [Source:MGI Symbol;Acc:MGI:3650631] | 14 | 53029773 | 53030234 | 1 |
| 14:52789208_C/G | 14 | 52.78921 | 1.824103 | ENSMUSG00000096478 | ENSMUSG00000096478:upstream_gene_variant | T cell receptor alpha variable 9D-1 [Source:MGI Symbol;Acc:MGI:3650631] | 14 | 53029773 | 53030234 | 1 |
| 14:52789212_G/C | 14 | 52.78921 | 1.824103 | ENSMUSG00000096478 | ENSMUSG00000096478:upstream_gene_variant | T cell receptor alpha variable 9D-1 [Source:MGI Symbol;Acc:MGI:3650631] | 14 | 53029773 | 53030234 | 1 |
| 14:52789261_T/C | 14 | 52.78926 | 1.824103 | ENSMUSG00000096478 | ENSMUSG00000096478:upstream_gene_variant | T cell receptor alpha variable 9D-1 [Source:MGI Symbol;Acc:MGI:3650631] | 14 | 53029773 | 53030234 | 1 |
| 14:52789262_G/A | 14 | 52.78926 | 1.824103 | ENSMUSG00000096478 | ENSMUSG00000096478:upstream_gene_variant | T cell receptor alpha variable 9D-1 [Source:MGI Symbol;Acc:MGI:3650631] | 14 | 53029773 | 53030234 | 1 |
| 14:52789328_A/G | 14 | 52.78933 | 1.824103 | ENSMUSG00000096478 | ENSMUSG00000096478:upstream_gene_variant | T cell receptor alpha variable 9D-1 [Source:MGI Symbol;Acc:MGI:3650631] | 14 | 53029773 | 53030234 | 1 |
| 14:52790819_C/A | 14 | 52.79082 | 1.824103 | ENSMUSG00000095426,ENSMUSG00000096478 | ENSMUSG00000095426:upstream_gene_variant,ENSMUSG00000096478:upstream_gene_variant | NA | NA | NA | NA | NA |
| 14:52790876_A/G | 14 | 52.79088 | 1.824103 | ENSMUSG00000095426,ENSMUSG00000096478 | ENSMUSG00000095426:upstream_gene_variant,ENSMUSG00000096478:upstream_gene_variant | NA | NA | NA | NA | NA |
| 14:52790883_G/T | 14 | 52.79088 | 1.824103 | ENSMUSG00000095426,ENSMUSG00000096478 | ENSMUSG00000095426:upstream_gene_variant,ENSMUSG00000096478:upstream_gene_variant | NA | NA | NA | NA | NA |
| 14:52790895_T/C | 14 | 52.7909 | 1.824103 | ENSMUSG00000095426,ENSMUSG00000096478 | ENSMUSG00000095426:upstream_gene_variant,ENSMUSG00000096478:upstream_gene_variant | NA | NA | NA | NA | NA |
| 14:52790896_G/A | 14 | 52.7909 | 1.824103 | ENSMUSG00000095426,ENSMUSG00000096478 | ENSMUSG00000095426:upstream_gene_variant,ENSMUSG00000096478:upstream_gene_variant | NA | NA | NA | NA | NA |
| 14:52790914_C/T | 14 | 52.79091 | 1.824103 | ENSMUSG00000095426,ENSMUSG00000096478 | ENSMUSG00000095426:upstream_gene_variant,ENSMUSG00000096478:upstream_gene_variant | NA | NA | NA | NA | NA |
| 14:52790958_G/A | 14 | 52.79096 | 1.824103 | ENSMUSG00000095426,ENSMUSG00000096478 | ENSMUSG00000095426:upstream_gene_variant,ENSMUSG00000096478:upstream_gene_variant | NA | NA | NA | NA | NA |
| 14:52791168_A/G | 14 | 52.79117 | 1.824103 | ENSMUSG00000095426,ENSMUSG00000096478 | ENSMUSG00000095426:upstream_gene_variant,ENSMUSG00000096478:upstream_gene_variant | NA | NA | NA | NA | NA |
| 14:52791172_A/G | 14 | 52.79117 | 1.824103 | ENSMUSG00000095426,ENSMUSG00000096478 | ENSMUSG00000095426:upstream_gene_variant,ENSMUSG00000096478:upstream_gene_variant | NA | NA | NA | NA | NA |
| 14:52791220_G/A | 14 | 52.79122 | 1.824103 | ENSMUSG00000095426,ENSMUSG00000096478 | ENSMUSG00000095426:upstream_gene_variant,ENSMUSG00000096478:upstream_gene_variant | NA | NA | NA | NA | NA |
| 14:52791406_T/C | 14 | 52.79141 | 1.824103 | ENSMUSG00000095426,ENSMUSG00000096478 | ENSMUSG00000095426:upstream_gene_variant,ENSMUSG00000096478:upstream_gene_variant | NA | NA | NA | NA | NA |
| 14:52791637_G/A | 14 | 52.79164 | 1.824103 | ENSMUSG00000095426,ENSMUSG00000096478 | ENSMUSG00000095426:upstream_gene_variant,ENSMUSG00000096478:upstream_gene_variant | NA | NA | NA | NA | NA |
| 14:52791700_G/A | 14 | 52.7917 | 1.824103 | ENSMUSG00000095426,ENSMUSG00000096478 | ENSMUSG00000095426:upstream_gene_variant,ENSMUSG00000096478:upstream_gene_variant | NA | NA | NA | NA | NA |
| 14:52791711_C/T | 14 | 52.79171 | 1.824103 | ENSMUSG00000095426,ENSMUSG00000096478 | ENSMUSG00000095426:upstream_gene_variant,ENSMUSG00000096478:upstream_gene_variant | NA | NA | NA | NA | NA |
| 14:52791966_A/G | 14 | 52.79197 | 1.880665 | ENSMUSG00000095426,ENSMUSG00000096478 | ENSMUSG00000095426:upstream_gene_variant,ENSMUSG00000096478:upstream_gene_variant | NA | NA | NA | NA | NA |
| 14:52792164_G/T | 14 | 52.79216 | 1.811398 | ENSMUSG00000095426,ENSMUSG00000096478 | ENSMUSG00000095426:upstream_gene_variant,ENSMUSG00000096478:upstream_gene_variant | NA | NA | NA | NA | NA |
| 14:52793524_C/T | 14 | 52.79352 | 1.811398 | ENSMUSG00000095426,ENSMUSG00000096478 | ENSMUSG00000095426:upstream_gene_variant,ENSMUSG00000096478:downstream_gene_variant | NA | NA | NA | NA | NA |
| SV_14_52794325_52799325 | 14 | 52.79683 | 1.811398 | NA | NA | NA | NA | NA | NA | NA |
| 14:52802543_C/T | 14 | 52.80254 | 1.811398 |  | intergenic_variant | NA | NA | NA | NA | NA |
| 14:52802563_G/A | 14 | 52.80256 | 1.811398 |  | intergenic_variant | NA | NA | NA | NA | NA |
| 14:52802736_C/T | 14 | 52.80274 | 1.811398 |  | intergenic_variant | NA | NA | NA | NA | NA |
| 14:52803018_A/G | 14 | 52.80302 | 1.811398 |  | intergenic_variant | NA | NA | NA | NA | NA |
| 14:52803166_G/A | 14 | 52.80317 | 1.811398 |  | intergenic_variant | NA | NA | NA | NA | NA |
| SV_14_52801325_52806325 | 14 | 52.80383 | 1.811398 | NA | NA | NA | NA | NA | NA | NA |
| SV_14_52816304_52818661 | 14 | 52.81748 | 1.811398 | NA | NA | NA | NA | NA | NA | NA |
| SV_14_52832325_52835325 | 14 | 52.83383 | 1.811398 | NA | NA | NA | NA | NA | NA | NA |
| 14:52905435_T/C | 14 | 52.90544 | 1.811398 | ENSMUSG00000093939,ENSMUSG00000094974 | ENSMUSG00000093939:downstream_gene_variant,ENSMUSG00000094974:downstream_gene_variant | NA | NA | NA | NA | NA |
| SV_14_52913325_52930325 | 14 | 52.92183 | 1.811398 | NA | NA | NA | NA | NA | NA | NA |
| SV_14_52932325_52950325 | 14 | 52.94133 | 1.811398 | NA | NA | NA | NA | NA | NA | NA |
| SV_14_52947325_52950325 | 14 | 52.94883 | 1.811398 | NA | NA | NA | NA | NA | NA | NA |
| 14:52959387_T/C | 14 | 52.95939 | 1.811398 | ENSMUSG00000096610 | ENSMUSG00000096610:downstream_gene_variant | NA | NA | NA | NA | NA |
| rs580173868 | 14 | 53.0116 | 1.811398 | ENSMUSG00000095756 | ENSMUSG00000095756:downstream_gene_variant | T cell receptor alpha variable 6D-7 [Source:MGI Symbol;Acc:MGI:2686335] | 14 | 53245069 | 53245555 | 1 |
| 14:53036863_A/C | 14 | 53.03686 | 1.811398 | ENSMUSG00000095643 | ENSMUSG00000095643:downstream_gene_variant | T cell receptor alpha variable 13D-3 [Source:MGI Symbol;Acc:MGI:5293419] | 14 | 53270305 | 53270876 | 1 |
| 14:53041864_G/A | 14 | 53.04186 | 1.728944 | ENSMUSG00000076795 | ENSMUSG00000076795:upstream_gene_variant | T cell receptor alpha variable 8D-2 [Source:MGI Symbol;Acc:MGI:3647760] | 14 | 53279885 | 53280348 | 1 |
| 14:53041929_A/T | 14 | 53.04193 | 1.728944 | ENSMUSG00000076795 | ENSMUSG00000076795:upstream_gene_variant | T cell receptor alpha variable 8D-2 [Source:MGI Symbol;Acc:MGI:3647760] | 14 | 53279885 | 53280348 | 1 |
| 14:53041942_A/G | 14 | 53.04194 | 1.728944 | ENSMUSG00000076795 | ENSMUSG00000076795:upstream_gene_variant | T cell receptor alpha variable 8D-2 [Source:MGI Symbol;Acc:MGI:3647760] | 14 | 53279885 | 53280348 | 1 |
| 14:53041947_C/T | 14 | 53.04195 | 1.728944 | ENSMUSG00000076795 | ENSMUSG00000076795:upstream_gene_variant | T cell receptor alpha variable 8D-2 [Source:MGI Symbol;Acc:MGI:3647760] | 14 | 53279885 | 53280348 | 1 |
| 14:53042136_A/C | 14 | 53.04214 | 1.728944 | ENSMUSG00000076795 | ENSMUSG00000076795:upstream_gene_variant | T cell receptor alpha variable 8D-2 [Source:MGI Symbol;Acc:MGI:3647760] | 14 | 53279885 | 53280348 | 1 |
| 14:53042862_A/T | 14 | 53.04286 | 1.728944 | ENSMUSG00000076796,ENSMUSG00000076795 | ENSMUSG00000076796:upstream_gene_variant,ENSMUSG00000076795:missense_variant | NA | NA | NA | NA | NA |
| 14:53044919_G/A | 14 | 53.04492 | 1.728944 | ENSMUSG00000076796,ENSMUSG00000076795 | ENSMUSG00000076796:upstream_gene_variant,ENSMUSG00000076795:downstream_gene_variant | NA | NA | NA | NA | NA |
| 14:53045000_A/G | 14 | 53.045 | 1.728944 | ENSMUSG00000076796,ENSMUSG00000076795 | ENSMUSG00000076796:upstream_gene_variant,ENSMUSG00000076795:downstream_gene_variant | NA | NA | NA | NA | NA |
| 14:53045097_A/C | 14 | 53.0451 | 1.728944 | ENSMUSG00000076796,ENSMUSG00000076795 | ENSMUSG00000076796:upstream_gene_variant,ENSMUSG00000076795:downstream_gene_variant | NA | NA | NA | NA | NA |
| 14:53045159_A/G | 14 | 53.04516 | 1.728944 | ENSMUSG00000076796,ENSMUSG00000076795 | ENSMUSG00000076796:upstream_gene_variant,ENSMUSG00000076795:downstream_gene_variant | NA | NA | NA | NA | NA |
| 14:53045161_C/A | 14 | 53.04516 | 1.728944 | ENSMUSG00000076796,ENSMUSG00000076795 | ENSMUSG00000076796:upstream_gene_variant,ENSMUSG00000076795:downstream_gene_variant | NA | NA | NA | NA | NA |
| 14:53045182_T/G | 14 | 53.04518 | 1.728944 | ENSMUSG00000076796,ENSMUSG00000076795 | ENSMUSG00000076796:upstream_gene_variant,ENSMUSG00000076795:downstream_gene_variant | NA | NA | NA | NA | NA |
| 14:53045192_C/T | 14 | 53.04519 | 1.728944 | ENSMUSG00000076796,ENSMUSG00000076795 | ENSMUSG00000076796:upstream_gene_variant,ENSMUSG00000076795:downstream_gene_variant | NA | NA | NA | NA | NA |
| 14:53045411_C/G | 14 | 53.04541 | 1.728944 | ENSMUSG00000076796,ENSMUSG00000076795 | ENSMUSG00000076796:upstream_gene_variant,ENSMUSG00000076795:downstream_gene_variant | NA | NA | NA | NA | NA |
| 14:53073124_G/A | 14 | 53.07312 | 1.728944 | ENSMUSG00000096329 | ENSMUSG00000096329:missense_variant | T cell receptor alpha variable 13D-4 [Source:MGI Symbol;Acc:MGI:4440515] | 14 | 53310220 | 53310731 | 1 |
| 14:53073138_C/A | 14 | 53.07314 | 1.728944 | ENSMUSG00000096329 | ENSMUSG00000096329:missense_variant | T cell receptor alpha variable 13D-4 [Source:MGI Symbol;Acc:MGI:4440515] | 14 | 53310220 | 53310731 | 1 |
| 14:53073141_G/A | 14 | 53.07314 | 1.728944 | ENSMUSG00000096329 | ENSMUSG00000096329:synonymous_variant | T cell receptor alpha variable 13D-4 [Source:MGI Symbol;Acc:MGI:4440515] | 14 | 53310220 | 53310731 | 1 |
| 14:53073144_G/C | 14 | 53.07314 | 1.728944 | ENSMUSG00000096329 | ENSMUSG00000096329:missense_variant | T cell receptor alpha variable 13D-4 [Source:MGI Symbol;Acc:MGI:4440515] | 14 | 53310220 | 53310731 | 1 |
| 14:53073499_T/G | 14 | 53.0735 | 1.728944 | ENSMUSG00000096329 | ENSMUSG00000096329:downstream_gene_variant | T cell receptor alpha variable 13D-4 [Source:MGI Symbol;Acc:MGI:4440515] | 14 | 53310220 | 53310731 | 1 |
| 14:53079883_A/C | 14 | 53.07988 | 1.728944 | ENSMUSG00000094619 | ENSMUSG00000094619:downstream_gene_variant | T cell receptor alpha variable 14D-3-DV8 [Source:MGI Symbol;Acc:MGI:3822547] | 14 | 53315909 | 53316502 | 1 |
| 14:53080779_C/T | 14 | 53.08078 | 1.728944 | ENSMUSG00000094619 | ENSMUSG00000094619:downstream_gene_variant | T cell receptor alpha variable 14D-3-DV8 [Source:MGI Symbol;Acc:MGI:3822547] | 14 | 53315909 | 53316502 | 1 |
| rs581643538 | 14 | 53.086 | 1.712135 |  | intergenic_variant | NA | NA | NA | NA | NA |
| rs583568686 | 14 | 53.0861 | 1.712135 |  | intergenic_variant | NA | NA | NA | NA | NA |
| rs581595096 | 14 | 53.10345 | 1.704822 | ENSMUSG00000092251,ENSMUSG00000076800 | ENSMUSG00000092251:downstream_gene_variant,ENSMUSG00000076800:upstream_gene_variant | NA | NA | NA | NA | NA |
| rs579329102 | 14 | 53.1035 | 1.704822 | ENSMUSG00000092251,ENSMUSG00000076800 | ENSMUSG00000092251:downstream_gene_variant,ENSMUSG00000076800:upstream_gene_variant | NA | NA | NA | NA | NA |
| rs581826557 | 14 | 53.10395 | 1.704822 | ENSMUSG00000092251,ENSMUSG00000076800 | ENSMUSG00000092251:downstream_gene_variant,ENSMUSG00000076800:upstream_gene_variant | NA | NA | NA | NA | NA |
| SV_14_53475156_53479681 | 14 | 53.47742 | 2.602466 | NA | NA | NA | NA | NA | NA | NA |
| SV_14_54091931_54091933 | 14 | 54.09193 | 2.232443 | NA | NA | NA | NA | NA | NA | NA |
| SV_14_54279758_54279776 | 14 | 54.27977 | 2.491346 | NA | NA | NA | NA | NA | NA | NA |
| SV_14_54342903_54343261 | 14 | 54.34308 | 2.523437 | NA | NA | NA | NA | NA | NA | NA |
| SV_14_54437885_54438132 | 14 | 54.43801 | 1.828878 | NA | NA | NA | NA | NA | NA | NA |
| SV_14_54500801_54501021 | 14 | 54.50091 | 1.827889 | NA | NA | NA | NA | NA | NA | NA |
| SV_14_54676272_54676274 | 14 | 54.67627 | 1.665028 | NA | NA | NA | NA | NA | NA | NA |

**Supplementary Table S12**. **Compilation of renal, hepatic, and cardiac GSH system QTL intervals.**

|  | Phenotype | LOD | Chr | QTL Position (Mbp) | QTL Position (cM) |  |
| --- | --- | --- | --- | --- | --- | --- |
| Renal | Total Glutathione | 6.298 | 2 | 52.657 (19.817 – 61.128) | 30.193 (14.085 – 35.317) |  |
|  | Total Glutathione | 6.428 | 11 | 100.804 (100.059 – 101.369) | 63.671 (63.395 – 64.832) |  |
|  | Total Glutathione | 7.105 | X | 51.602 (49.234 – 51.892) | 28.205 (25.870 – 28.316) |  |
|  | GSH | 6.249 | 2 | 52.657 (19.817 – 64.003) | 30.193 (14.085 – 37.364) |  |
|  | GSH | 6.664 | 11 | 100.810 (100.059 – 101.369) | 63.682 (63.395 – 64.832) |  |
|  | GSH | 6.962 | X | 51.602 (49.234 – 51.892) | 28.205 (25.870 – 28.316) |  |
|  | GSSG | 6.044 | 1 | 144.970 (143.603 – 148.590) | 63.069 (62.513 – 63.371) |  |
|  | GSSG | 6.048 | 16 | 62.938 (61.291 – 72.202) | 36.457 (36.121 – 40.793) |  |
|  | GSH/GSSG | 6.032 | 13 | 71.522 (71.458 – 72.209) | 38.009 (37.975 – 38.731) |  |
|  | E_h_ | 6.258 | 14 | 22.959 (22.359 – 23.926) | 12.791 (12.204 – 14.152) |  |
|  | BUN | 6.06 | 6 | 127.602 (125.386 – 128.618) | 62.438 (59.395 – 63.065) |  |
| Hepatic | Total Glutathione | 6.748 | 14 | 22.506 (22.058 – 22.528) | 12.355 (12.035 – 12.377) |  |
|  |  |  |  |  |  |  |
|  | Total Glutathione | 6.011 | 18 | 51.537 (35.120 – 76.532) | 28.097 (18.887 – 51.726) |  |
|  | GSH | 6.755 | 14 | 22.506 (22.058 – 22.528) | 12.355 (12.035 – 12.377) |  |
|  | GSSG | 6.283 | 1 | 21.043 (18.793 – 22.050) | 6.502 (5.847 – 8.186) |  |
|  | GSSG | 6.178 | 2 | 91.523 (78.897 – 91.656) | 50.598 (47.166 – 50.641) |  |
|  | GSSG | 6.149 | 18 | 53.075 (50.199 – 82.487) | 28.328 (27.234 – 55.913) |  |
|  | GSH/GSSG | 6.407 | 11 | 6.017 (5.568 – 7.583) | 3.906 (3.664 – 4.782) |  |
|  | GSH/GSSG | 8.224 | 16 | 8.998 (8.865 – 10.077) | 4.779 (4.433 – 5.470) |  |
|  | E_h_ | 6.48 | 16 | 6.997 (3.526 – 8.225) | 3.373 (2.074 – 3.621) |  |
|  | E_h_ | 8.598 | 16 | 8.998 (8.865 – 10.324) | 4.779 (4.433 – 5.579) |  |
|  | NADPH | 6.352 | 2 | 173.408 (173.133 – 174.508) | 96.424 (95.769 – 97.976) |  |
|  | NADPH | 6.612 | 12 | 28.626 (28.578 – 29.450) | 10.987 (10.975 – 11.927) |  |
|  | NADPH | 6.287 | 17 | 51.366 (50.164 – 52.542) | 26.684 (25.861 – 27.425) |  |
|  | NADP^+^ | 7.032 | 3 | 110.517 (109.677 – 115.729) | 48.547 (48.380 – 50.024) |  |
|  | NADP^+^ | 6.59 | 8 | 61.237 (60.722 – 65.378) | 30.937 (30.901 – 32.757) |  |
|  | NADP^+^/NADPH | 7.637 | 12 | 28.626 (28.562 – 29.394) | 10.987 (10.972 – 11.864) |  |
|  | NADH | 6.062 | 14 | 100.731 (99.320 – 102.809) | 50.508 (49.350 – 52.844) |  |

| Cardiac | GSH | 7.4922555 | 14 | 54.23953 (48.909 - 55.303) | 27.551 (25.407 - 27.966) |  |
| --- | --- | --- | --- | --- | --- | --- |
|  | E_h_ | 6.96993055 | 14 | 54.23953 (48.905271 - 55.226142) | 27.551 (25.405 - 27.936) |  |
|  | GSH | 6.96857156 | 16 | 96.735776 (96.670 - 98.097) | 57.035 (57.016 - 57.841) |  |
|  | GSH | 6.59622423 | 19 | 57.209308 (57.981 - 57.523) | 52.157 (3.158 - 53.747) |  |
|  | E_h_ | 6.57350903 | 16 | 96.735776 (96.670035 - 98.096875) | 57.035 (57.016 - 57.841) |  |
|  | GSSG | 6.41088918 | 10 | 124.103853 (87.902 - 125.347) | 72.222 (43.709 - 72.548) |  |
|  | GSH/GSSG | 6.15378124 | 14 | 54.23953 (26.536 - 80.310) | 27.551 (11.224 - 42.809) |  |
|  | E_h_ | 6.14726648 | 19 | 57.209308 (3.263752 - 57.978364) | 52.157 (3.097 - 53.882) |  |
|  | GSH/GSSG | 6.10393796 | 5 | 38.838612 (37.152 - 122.875) | 20.787 (17.501 - 82.49) |  |


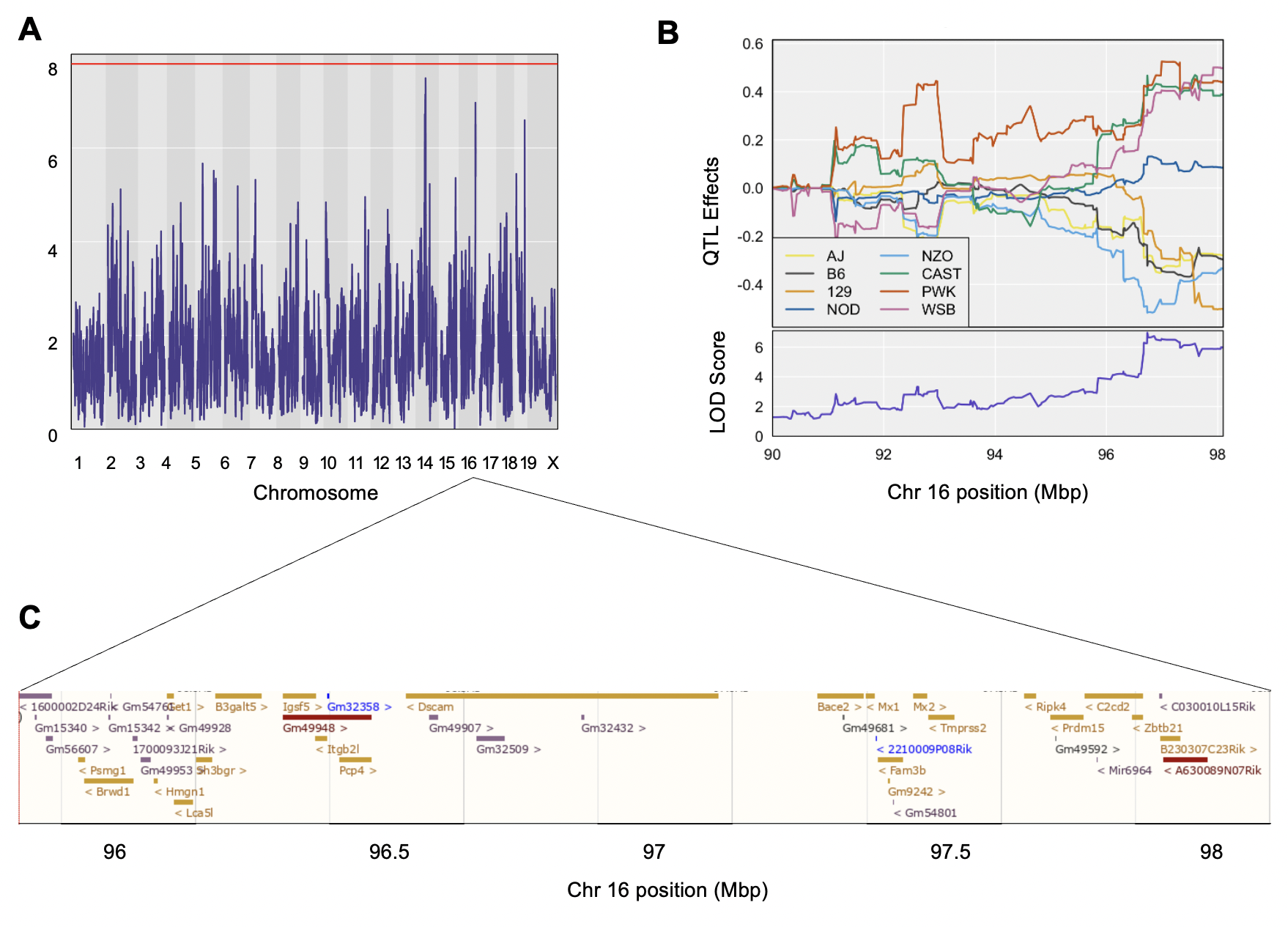
**Supplementary Figure S1. High-resolution QTL mapping of cardiac GSH reveals a suggestive peak on murine chromosome 16.** A. Genome-wide scan of cardiac GSH (nmol/μg) contains a QTL with LOD score 6.969 located at 96.735776 Mbp. The permutation-derived threshold is indicated by the red colored line at significance (α) level 0.05. B. Founder allele QTL effects for this locus. Each colored line represents one of eight DO founder alleles as indicated in the legend. The differences between strains are considered significant when the LOD score (bottom plot) crosses the significance thresholds established in panel A. **C.** Candidate genes found within the QTL interval extracted from the Ensembl genome browser.


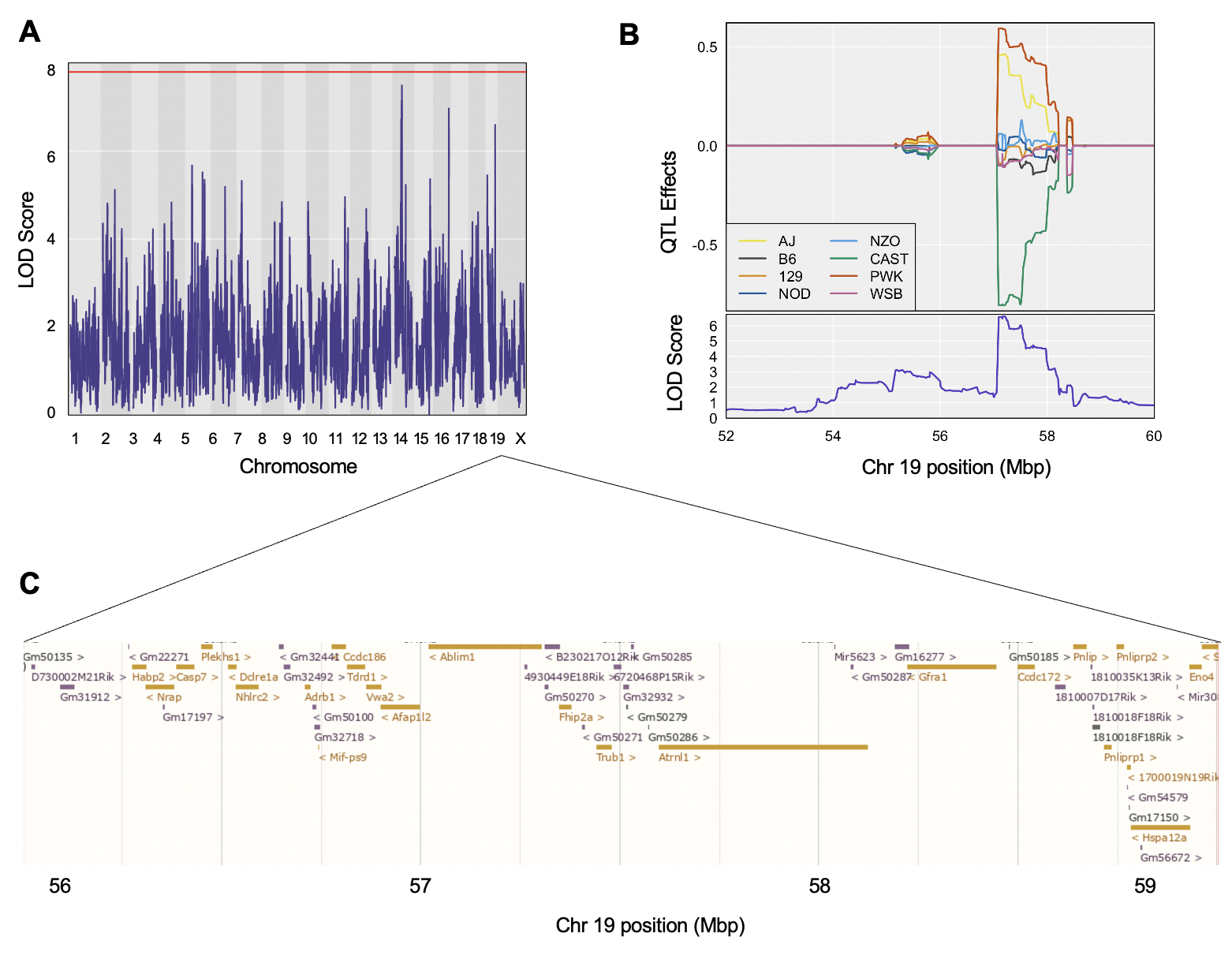


**Supplementary Figure S2.** **High-resolution QTL mapping of cardiac GSH reveals a suggestive peak on murine chromosome 19.** A. Genome-wide scan of cardiac GSH (nmol/μg) contains a QTL with LOD score 6.596 located at 57.209 Mbp. The permutation-derived threshold is indicated by the red colored line at significance (α) level 0.05. B. Founder allele QTL effects for this locus. Each colored line represents one of eight DO founder alleles as indicated in the legend. The differences between strains are considered significant when the LOD score (bottom plot) crosses the significance thresholds established in panel A. **C.** Candidate genes found within the QTL interval extracted from tbe Ensembl genome browser.

**
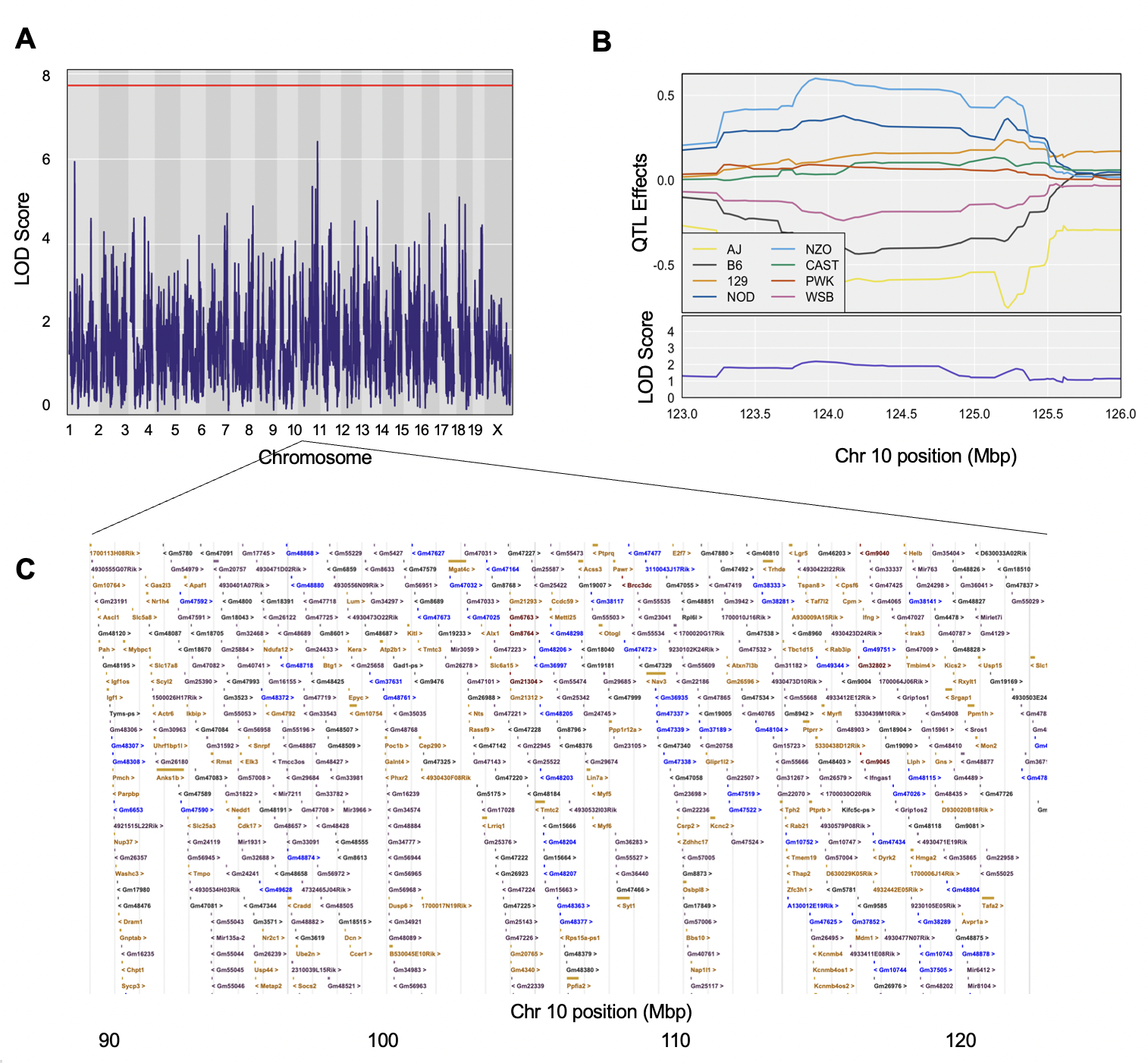
**

**Supplementary Figure S3. High-resolution QTL mapping of cardiac GSSG reveals a suggestive peak on murine chromosome 10.** A. Genome-wide scan of cardiac GSSG (nmol/μg) contains a QTL with LOD score 6.411 located at 124.11 Mbp. The permutation-derived threshold is indicated by the red colored line at significance (α) level 0.05. B. Founder allele QTL effects for this locus. Each colored line represents one of eight DO founder alleles as indicated in the legend. The differences between strains are considered significant when the LOD score (bottom plot) crosses the significance thresholds established in panel A. **C.** Candidate genes found within the QTL interval extracted from tbe Ensembl genome browser.


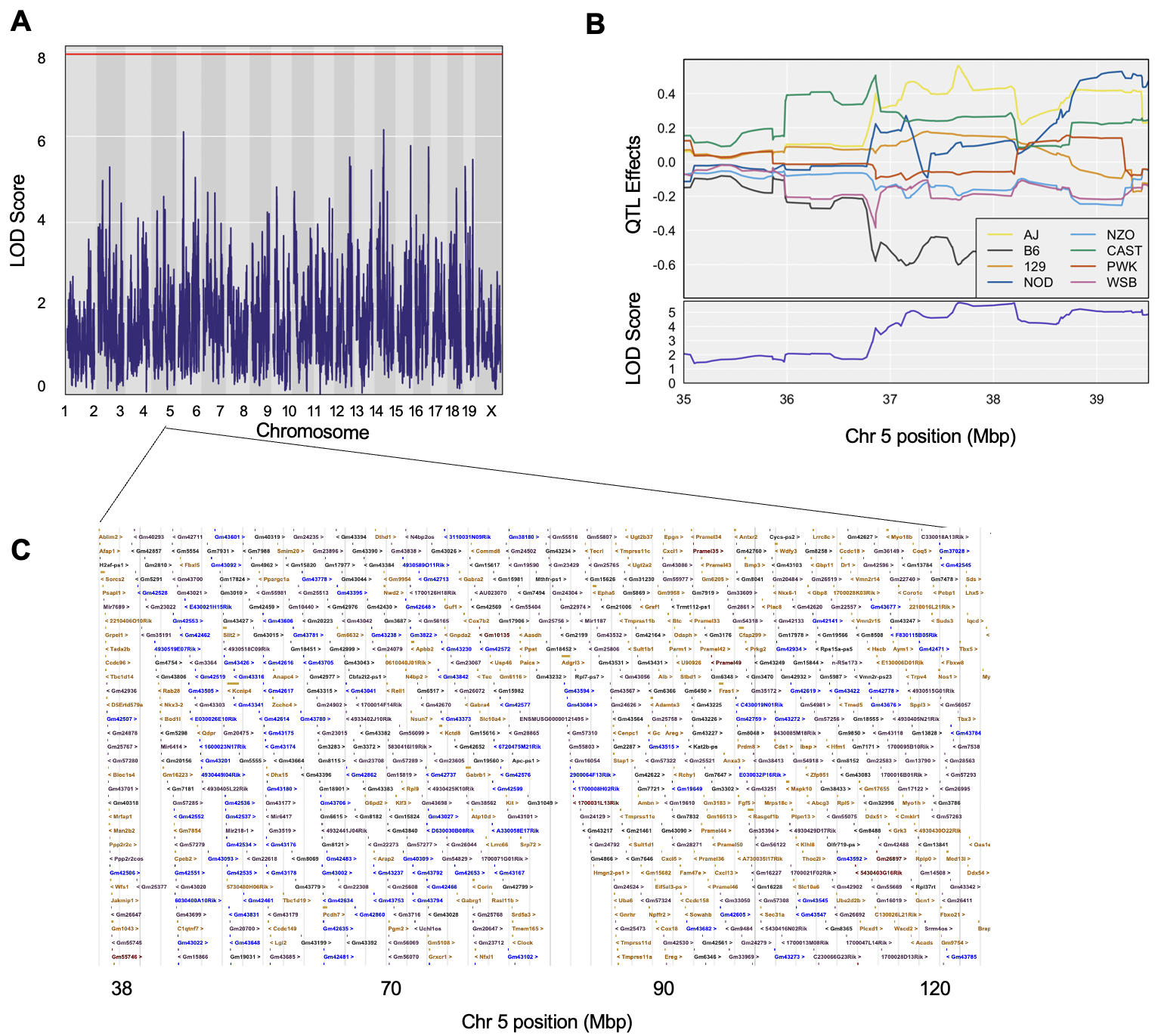


**Supplementary Figure S4.** **High-resolution QTL mapping of cardiac GSH/GSSG reveals a suggestive peak on murine chromosome 5**. A. Genome-wide scan of cardiac GSSG/GSSG contains a QTL with LOD score 6.103 located at 38.839 Mbp. The permutation-derived threshold is indicated by the red colored line at significance (α) level 0.05. B. Founder allele QTL effects for this locus. Each colored line represents one of eight DO founder alleles as indicated in the legend. The differences between strains are considered significant when the LOD score (bottom plot) crosses the significance thresholds established in panel A. **C.** Candidate genes found within the QTL interval extracted from tbe Ensembl genome browser.


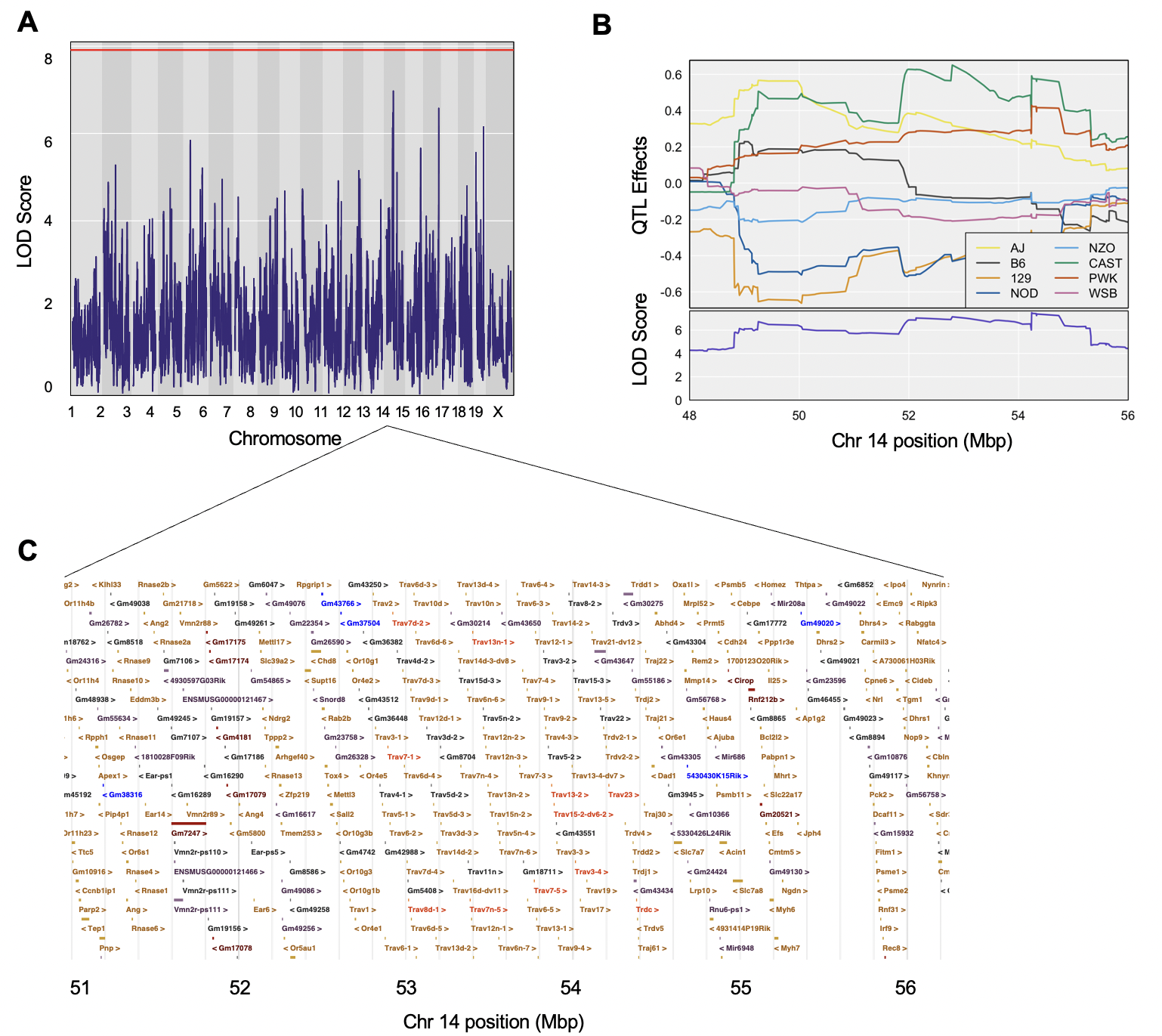


**Supplementary Figure S5.** **High-resolution QTL mapping of cardiac redox potential reveals a suggestive peak on murine chromosome 14.** A. Genome-wide scan of cardiac redox potential contains a QTL with LOD score 6.969 located at 54.240 Mbp. The permutation-derived threshold is indicated by the red colored line at significance (α) level 0.05. B. Founder allele QTL effects for this locus. Each colored line represents one of eight DO founder alleles as indicated in the legend. The differences between strains are considered significant when the LOD score (bottom plot) crosses the significance thresholds established in panel A. **C.** Candidate genes found within the QTL interval extracted from tbe Ensembl genome browser.


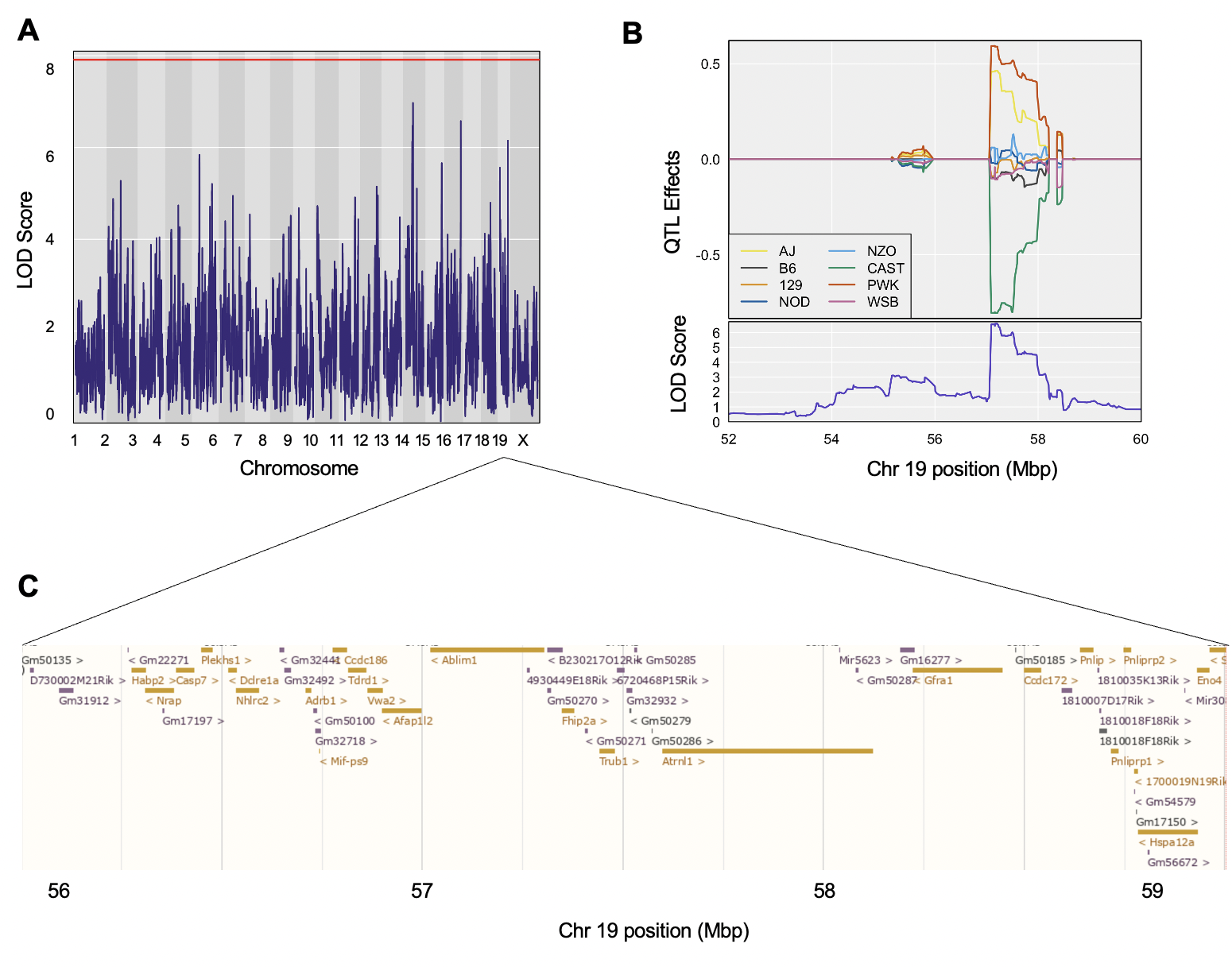


**Supplementary Figure S6.** **High-resolution QTL mapping of cardiac redox potential reveals a suggestive peak on murine chromosome 19**. A. Genome-wide scan of cardiac redox potential contains a QTL with LOD score 6.147 located at 57.209 Mbp. The permutation-derived threshold is indicated by the red colored line at significance (α) level 0.05. B. Founder allele QTL effects for this locus. Each colored line represents one of eight DO founder alleles as indicated in the legend. The differences between strains are considered significant when the LOD score (bottom plot) crosses the significance thresholds established in panel A. **C.** Candidate genes found within the QTL interval extracted from tbe Ensembl genome browser.

**Supplementary Figure S7. High-resolution QTL mapping of cardiac redox potential reveals a suggestive peak on murine chromosome 16**. A. Genome-wide scan of cardiac redox potential contains a QTL with
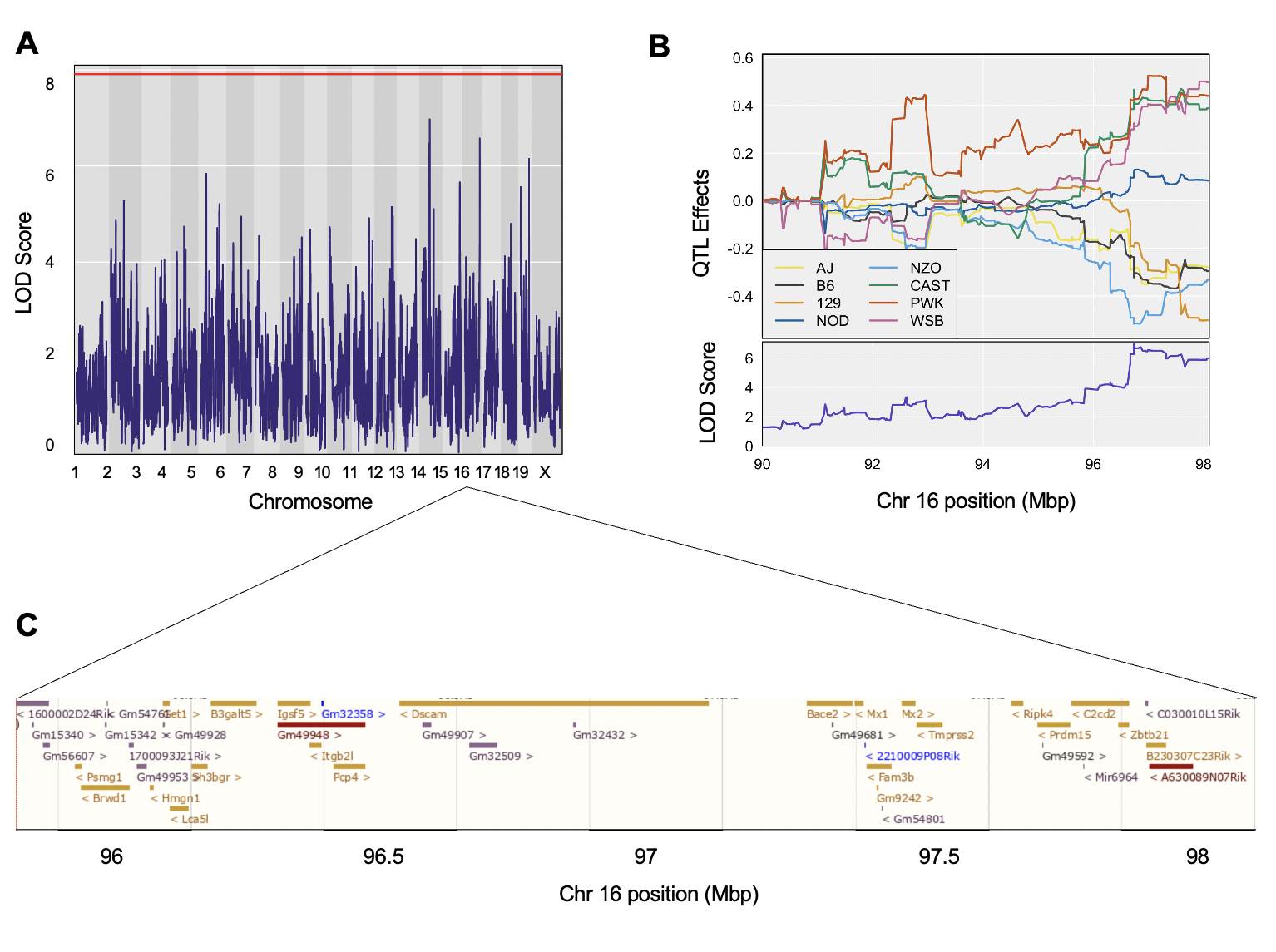
LOD score 6.574 located at 96.736 Mbp. The permutation-derived threshold is indicated by the red colored line at significance (α) level 0.05. B. Founder allele QTL effects for this locus. Each colored line represents one of eight DO founder alleles as indicated in the legend. The differences between strains are considered significant when the LOD score (bottom plot) crosses the significance thresholds established in panel A. **C.** Candidate genes found within the QTL interval extracted from tbe Ensembl genome browser.


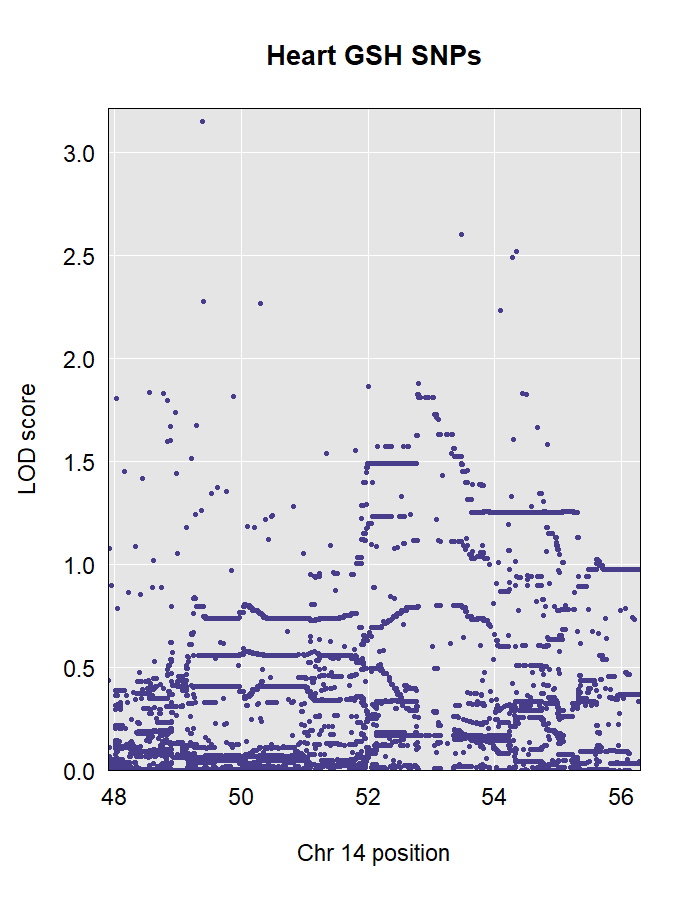


**Supplementary Figure S8. All known variants within +/- 1 Mbp of the 95% Bayesian credible interval for the Chr 14 GSH QTL.**

1. Petryszak, R., et al., *Expression Atlas update--an integrated database of gene and protein expression in humans, animals and plants.* Nucleic Acids Res, 2016. **44**(D1): p. D746-52.

2. Baldarelli, R.M., et al., *The mouse Gene Expression Database (GXD): 2021 update.* Nucleic Acids Research, 2020. **49**(D1): p. D924-D931.

3. Ashburner, M., et al., *Gene ontology: tool for the unification of biology. The Gene Ontology Consortium.* Nat Genet, 2000. **25**(1): p. 25-9.

4. Smith, C.L., C.-A.W. Goldsmith, and J.T. Eppig, *The Mammalian Phenotype Ontology as a tool for annotating, analyzing and comparing phenotypic information.* Genome Biology, 2004. **6**(1): p. R7.

5. Schriml, L.M., et al., *The Human Disease Ontology 2022 update.* Nucleic Acids Res, 2022. **50**(D1): p. D1255-d1261.
